# PI3K/AKT inhibition reverses R-CHOP resistance by destabilizing SOX2 in diffuse large B cell lymphoma

**DOI:** 10.7150/thno.41362

**Published:** 2020-02-10

**Authors:** Jianfeng Chen, Xiaowen Ge, Wei Zhang, Peipei Ding, Yiqun Du, Qi Wang, Ling Li, Lan Fang, Yujing Sun, Pingzhao Zhang, Yuzhen Zhou, Long Zhang, Xinyue Lv, Luying Li, Xin Zhang, Qunling Zhang, Kai Xue, Hongyu Gu, Qunying Lei, Jiemin Wong, Weiguo Hu

**Affiliations:** 1 Fudan University Shanghai Cancer Center and Institutes of Biomedical Sciences, Shanghai Medical College, Fudan University, Shanghai 200032, China.; 2 Department of Pathology, Zhongshan Hospital, Shanghai Medical College, Fudan University, Shanghai 200032, China.; 3 Department of Oncology, Shanghai Medical College, Fudan University, Shanghai 200032, China.; 4 Shanghai Key Laboratory of Regulatory Biology, Institute of Biomedical Sciences and School of Life Sciences, East China Normal University, Shanghai, China.

**Keywords:** DLBCL, drug resistance, PI3K/AKT, SOX2, cancer stem-like cells

## Abstract

Up to one-third of diffuse large B cell lymphoma (DLBCL) patients eventually develop resistance to R-CHOP regimen, while the remaining therapeutic options are limited. Thus, understanding the underlying mechanisms and developing therapeutic approaches are urgently needed.

**Methods**: We generated two germinal center B cell-like (GCB) and activated B cell-like (ABC) subtype R-CHO resistant DLBCL cell lines, of which the tumor-initiating capacity was evaluated by serial-transplantation and stemness-associated features including CD34 and CD133 expression, side population and ALDH1 activity were detected by flow cytometry or immunoblotting. Expression profiles of these resistant cells were characterized by RNA sequencing. The susceptibility of resistant cells to different treatments was evaluated by *in vitro* CytoTox-glo assay and in tumor-bearing mice. The expression levels of SOX2, phos-AKT, CDK6 and FGFR1/2 were detected in 12 R-CHOP-resistant DLBCL clinical specimens by IHC.

**Results**: The stem-like CSC proportion significantly increased in both resistant DLBCL subtypes. SOX2 expression level remarkably elevated in both resistant cell lines due to its phosphorylation by activated PI3K/AKT signaling, thus preventing ubiquitin-mediated degradation. Further, multiple factors, including BCR, integrins, chemokines and FGFR1/2 signaling, regulated PI3K/AKT activation. CDK6 in GCB subtype and FGFR1/2 in ABC subtype were SOX2 targets, whose inhibition potently re-sensitized resistant cells to R-CHOP treatment. More importantly, addition of PI3K inhibitor to R-CHOP completely suppressed the tumor growth of R-CHO-resistant DLBCL cells, most likely by converting CSCs to chemo-sensitive differentiated cells.

**Conclusions**: The PI3K/AKT/SOX2 axis plays a critical role in R-CHOP resistance development and the pro-differentiation therapy against CSCs proposed in this study warrants further study in clinical trials for the treatment of resistant DLBCL.

## Introduction

The success of conventional anticancer therapy is frequently hindered by drug resistance. Such acquired resistance results at least partly from intratumoral heterogeneity that is accounted for the predominant differentiated cancer cells and the subpopulation of cancer stem-like cells (CSCs). Although most differentiated cells are eliminated by conventional therapy, CSCs survive, which may drive drug resistance development 1, 2. CSCs are characterized by distinct surface proteins, self-renewal, differentiation, quiescence, and a high association with drug resistance, recurrence and metastasis 2, 3. Therefore, a combination of conventional therapy and CSC-specific therapy is strongly indicated and being pursued 4.

SOX2, a pluripotency-associated transcription factor, is critical for embryonic and induced pluripotent stem cells 5; however, its expression is highly detrimental in at least 25 different cancers 6. In contrast with numerous studies focusing on *SOX2* regulation by non-coding RNAs, there have been limited reports concerning transcriptional regulation and post-translational modifications^7^. PI3K/AKT1 signaling is a master regulator not only in tumorigenesis, tumor progression, and drug resistance 8, 9 but also in CSC biology 10. Interestingly, PI3K/AKT1 may suppress SOX2 ubiquitination via a methylation (K119)-phosphorylation (T118) switch in SOX2, thus stabilizing SOX2 11.

Non-Hodgkin lymphoma ranks in the top 10 causes of cancer mortality, and diffuse large B cell lymphoma (DLBCL) is the most common subtype 12. DLBCL can be subdivided into three distinct cell-of-origin subtypes: germinal center B cell-like (GCB), activated B cell-like (ABC), and 10-20% primary mediastinal B cell lymphoma (PMBL) subtypes 13. Although more than half of DLBCL patients can be cured, mainly by R-CHOP (rituximab/R, cyclophosphamide/C, doxorubicin/H, vincristine/O, and prednisone/P) regimens 14, up to one-third of patients will eventually develop relapsed/refractory disease 15. Our growing understanding of the molecular basis of resistance has led to the development of a large number of novel interventions, however, they are only being tested in phase I or II trials, and no single agent or regimen provides long-term disease control 16. Thus, novel therapeutic approaches for relapsed/refractory DLBCL are urgently needed.

Here we found a remarkably elevated proportion of CSCs in resistant DLBCL cells, whose stemness was regulated by the activated PI3K/AKT1/SOX2 axis. Further, PI3K/AKT inhibitor converted CSCs to differentiated tumor cells by reducing SOX2 level, thus preventing the growth of implanted resistant cells when combined with the R-CHOP regimen.

## Materials and Methods

A complete description of the methods is provided in the supplemental material.

### DLBCL tissue samples, cell lines and reagents

We examined the medical history of all DLBCL patients from 2008 to 2015 at Fudan University Shanghai Cancer Center and found a total of 12 patients who simultaneously had both paraffin-embedded tissue samples from the initial visit and from relapse. DLBCL cases were subgrouped into GCB (6 cases) or ABC (6 cases) molecular subtypes based on the Hans immunohistochemistry algorithm. Additional information is provided in the supplemental material.

### Aldefluor Assay

ALDH1 is a selectable marker for multiple kinds of normal and cancer stem cells, including hematopoietic stem cells 17, 18. Thus, we evaluated cancer stem-like cell numbers in hematopoietic malignancies using an ALDEFLUOR™ kit (StemCell Technologies, Vancouver, BC, CA) to detect ALDH1^+^ cells. Details are described in the supplemental material.

### FACS Analysis

Flow cytometric analysis was performed on a Cytomics FC500 MPL instrument (Beckman Coulter, Brea, CA) and analyzed with FlowJo software (Ashland, OR). We performed cell sorting with a MoFlo XDP instrument (Beckman Coulter, Brea, CA). Details are described in the supplemental material.

### Xenograft Model

All the animal experiments were conducted in strict accordance with experimental protocols approved by the Animal Ethics Committee at Shanghai Medical School, Fudan University. Eight-week-old female SCID mice were purchased from Slac Laboratory Animal Center (Shanghai, China) for injection with RCHO-resistant DLBCL cells. The methods of drug delivery based on the clinical usage for one cycle are indicated in Supplemental material. Tumor growth was monitored by bioluminescence at 50, 70 and 90 days after implantation using an In Vivo MS FX PRO system (Bruker, Billerica, MA). The surviving mice were euthanized and dissected at 120 days after xenografting, and no intraperitoneal tumors were found. Tumor tissues were immediately collected from the moribund mice after euthanatized by CO_2_. Additional information including serial-transplantation for detecting tumor-initiating capacity of RCHO-resistant cells is provided in the supplemental material.

### RNA Sequencing and Bioinformatic Analysis

Total RNA was extracted from LY8-ORI, LY8-R, LY8-CHO, LY8-RCHO, NU-DUL-1, NU-DUL-1-R, NU-DUL-1-CHO, and NU-DUL-1-RCHO cells with TRIzol reagent (Invitrogen, Grand Island, NY). The total RNA from each group from 3 different passages was pooled separately. RNA sequencing (RNA-seq) and bioinformatics analysis were conducted by Shanghai Novelbio Ltd. 19. Details are decribed in the supplemental material.

### Statistical Methods

The data are presented as mean ± SD unless otherwise specified. Significant differences between two groups were determined using two-tailed Student's t-test for unpaired data, and *P*<0.05 was considered statistically significant. For IHC staining scores of tissue microarrays, significance was determined by two-tailed paired t-test, and *P*<0.05 was considered statistically significant. For the total photon flux of animal models, significance was determined by a one-tailed Mann-Whitney test, and *P*<0.05 was considered statistically significant. We applied a Mantel-Cox test to compare the survival rates between the two groups of xenograft models, and *P*<0.05 was considered statistically significant. All statistical analyses were performed by GraphPad Prism 7 (La Jolla, CA).

## Results

### Resistance of stem-like resistant DLBCL cells was regulated by SOX2

**Figure 1:**
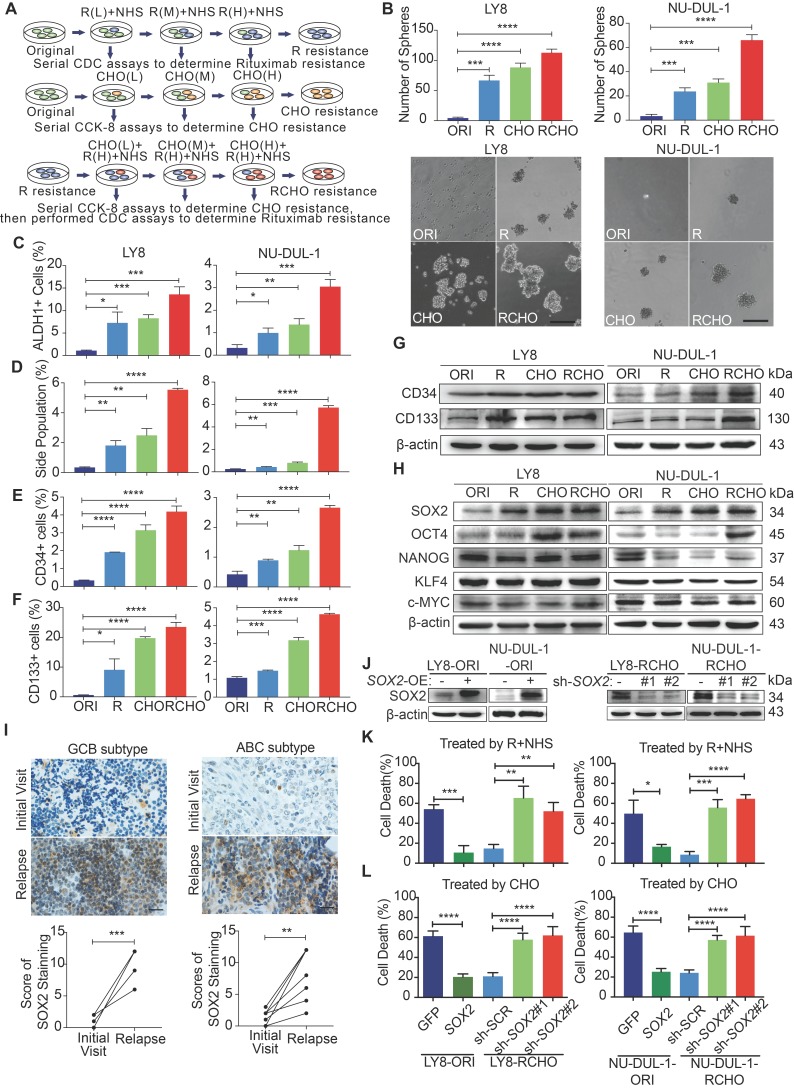
** Resistant DLBCL cells displayed stem-like features and their resistance was regulated by the SOX2 level.** (**A**) A schematic diagram of the generation of resistant DLBCL cells. L, M, and H represents the low, middle, and high dosages, respectively. (**B**-**G**) The proportion of CSCs in resistant DLBCL cells increased. The resistant cells exhibited enhanced sphere-forming capacity (B) and a greater percentage of ALDH1^+^ (C), side population fraction (D), CD34^+^ (E) and CD133^+^ cells (F) than original cells. Moreover, immunoblotting assay further revealed that CD34 and CD133 expressions increased, while PAX5 expression reduced in resistant cells (G). Scale bar: 100 μm. (**H**) Among the detected stemness-associated transcription factors, only SOX2 expression was gradually elevated along with the tendency toward resistance. (**I**) SOX2 expression significantly increased in the relapsed GCB (up, 6 pairs) and ABC (bottom, 6 pairs) subtype clinical tissues *vs* the paired tissues from the initial visit. Left: a representative image; Right: the quantitative score for SOX2 staining. Scale bar: 20 µm. (**J**) The SOX2 levels were confirmed by Immunoblotting assay after ectopic expressing SOX2 in the original cells and reducing SOX2 levels in RCHO-resistant cells. (**K**) CDC assays: ectopic SOX2 expression reduced the susceptibility of original cells to R-mediated CDC, whereas SOX2 silencing exhibited the opposite effect in RCHO-resistant cells. (**L**) CytoTox-Glo cytotoxicity assays: ectopic expression of SOX2 reduced the cytotoxic effect of CHO in original cells, whereas SOX2 silencing retrieved sensitivity to CHO in RCHO-resistant cells. The data are presented as mean ± SD; n=3. *P<0.05, **P<0.01, ***P<0.001, and ****P<0.0001.

Antibody-dependent cellular cytotoxicity (ADCC) and complement-dependent cytotoxicity (CDC) are the major mechanisms underlying R therapeutic effect 20, 21. Resistance to ADCC may be derived from intrinsic features of immune cells of individual patient, such as *FcGRIIIA* polymorphism and CD47 expression 22, 23; while resistance to CDC is mediated by a low expression level of CD20 and/or high expression levels of membrane-bound complement regulatory proteins (mCRPs), including CD46, CD55 and especially CD59 21, 24. CHO (cyclophosphamide/C, doxorubicin/H, vincristine/O) suppresses tumor cell duplication by inducing DNA damage or binding to tubulin, and prodrug C must be metabolically activated *in vivo* into 4-hydroxycyclophosphamide (4-HC) to exert effect. Therefore, the R-resistant cells were prepared by treatment with escalating rituximab concentrations in the presence of NHS, and the CHO-resistant cells were obtained by treatment with doxorubicin and vincristine at the clinical ratio of 50:1.4 by escalating the concentration, then treated with 2 µg/mL 4-HC. Using the same approach, we generated RCHO- resistant cells from the R- resistant cells (Figure 1A). To maintain RCHO resistance, the LY8-RCHO and NU-DUL-1-RCHO cells were treated with doxorubicin, vincristine and 4-HC every 21 days following treatment with rituximab and NHS. The resistance to CDC (Figure S1A) and to chemotherapy (Figure S1B) was validated, and cross-resistance to R-mediated CDC and CHO-mediated chemotherapy was observed to a certain degree. Furthermore, the resistant cells exhibited stem-like spheres with varying sizes compared with the original (ORI) cells, and the number of spheres increased gradually in order of R, CHO, and RCHO (Figure 1B). Although few studies have experimentally demonstrated the presence of CSCs in non-Hodgkin lymphoma, they are still proposed to exist 25, 26. Thus, we employed ALDH1 and side population in CSCs of various cancer types and CD34 and CD133 in CSCs of leukemia 27 to ascertain the stemness of resistant DLBCL cells. The results showed a significantly elevated proportion of ALDH1^+^, side population, CD34^+^ and CD133^+^ cells in the resistant cells (Figures 1C-F, and Figures S1C-F), in which the increase of CD34 and CD133 levels were further verified by immunoblotting (Figure 1G). Because cancer stem cell is a functional definition^28^, we used serial-transplantation assay to reveal that both RCHO-resistant LY8 and NU-DUL-1 cells displayed higher tumor-initiating capacity than original cells (Table 1 and Figure S2). Together, these results strongly demonstrated that CSCs were highly enriched in the resistant DLBCL cells.

To examine the molecular regulation of stemness in the resistant cells, we detected the expression of core stemness-associated transcription factors, SOX2, OCT4, NANOG, KLF4 and c-Myc. As shown in Figure 1H, OCT4 level obviously increased in CHO- and RCHO-resistant LY8 cells and in RCHO-resistant NU-DUL-1 cells compared with their related original cells; however, only SOX2 level gradually increased along with the tendency toward resistance. Moreover, in clinical specimens from DLBCL patients, we observed that SOX2 staining in both GCB and ABC subtypes was markedly increased in relapsed tissues compared with their paired tissues from the initial visit (Figure 1I and Figure S3A). Ectopic SOX2 expression (Figure 1J) considerably reduced cell death induced by R (Figure 1K) or CHO (Figure 1L) in original LY8 or NU-DUL-1 cells; in contrast, silencing SOX2 by shRNAs (Figure 1J) induced the opposite effect (Figures 1K and L) in RCHO-resistant LY8 or NU-DUL-1 cells. These results revealed that CSCs strongly enriched in resistant DLBCL cells, and elevated SOX2 was responsible for the development of drug resistance to R and CHO.

### PI3K/AKT1 signaling phosphorylated and thus stabilized SOX2 against ubiquitination-mediated degradation

**Figure 2:**
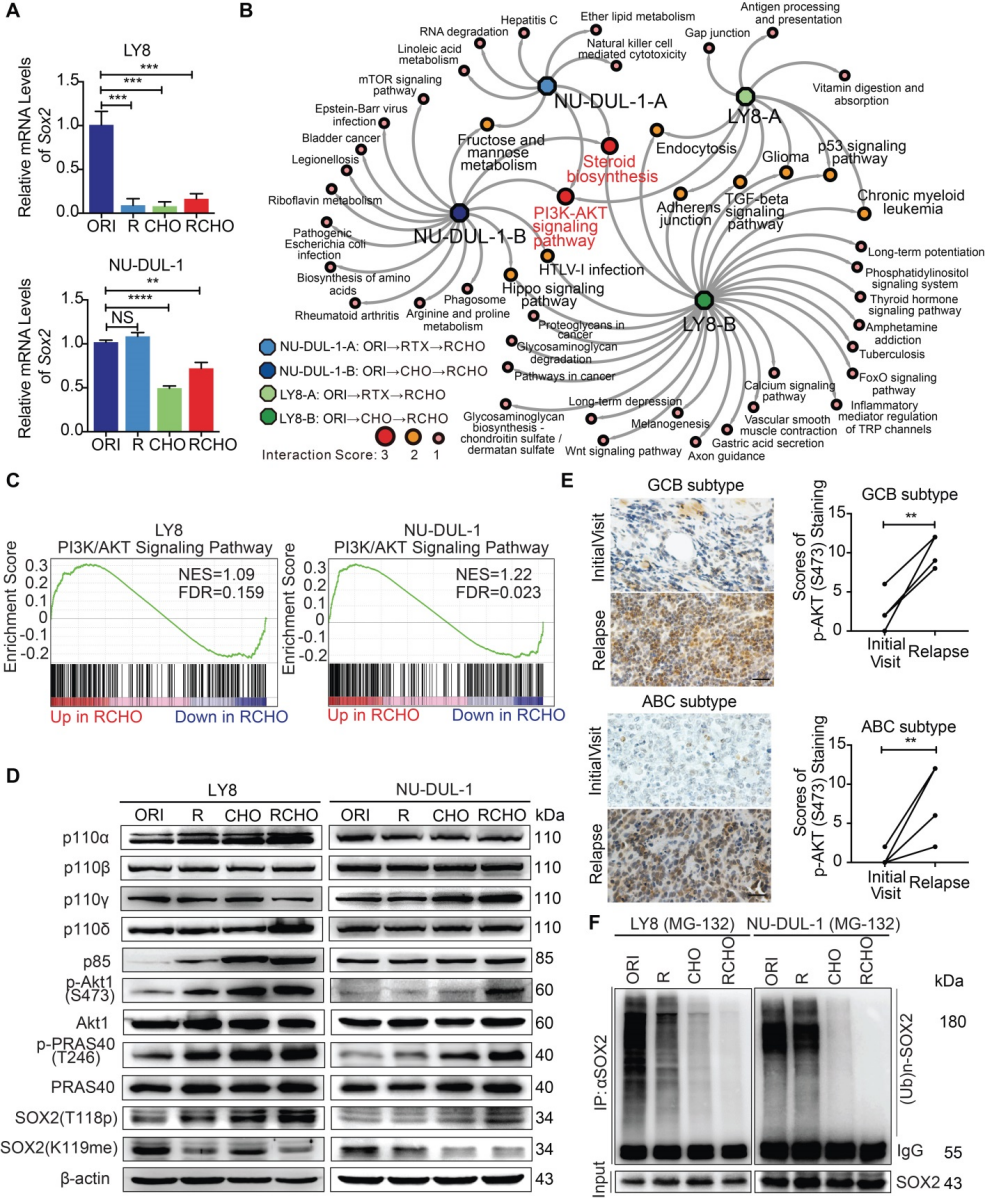
** PI3K-AKT1 phosphorylates and further stabilizes SOX2 against ubiquitination-mediated degradation in resistant DLBCL cells.** (**A**) The *SOX2* mRNA levels were significantly reduced in almost resistant DLBCL cells, as determined by qRT-PCR. (**B**) PI3K/AKT signaling together with steroid biosynthesis exhibited the highest interaction score in the network generated by Cytocape software using the RNA-seq data. (**C**) GSEA for the PI3K/AKT signature in RCHO-resistant LY8 (left) and NU-DUL-1 (right) cells. FDR<0.25 was considered significant. (**D**) PI3K/AKT1 signaling was activated, thus promoting the phosphorylation and reducing the methylation of SOX2. (**E**) PI3K/AKT1 signaling was markedly activated in relapsed GCB (up, 6 pairs) and ABC (bottom, 6 pairs) subtype clinical tissues *vs* the paired tissues from the initial visit. Left: a representative image; Right: the quantitative score for p-AKT1 (S473) staining. Scale bar: 20 μm. (**F**) Ubiquitination of SOX2 was reduced in resistant DLBCL cells after 10 μM MG-132 treatment for 8 hours. The data are presented as mean ± SD; n=3. **P<0.01, ***P<0.001, and ****P<0.0001.

We next investigated the mechanism responsible for SOX2 upregulation in resistant DLBCL cells. Unexpectedly, the *SOX2* mRNA level significantly declined in resistant cells, particularly in resistant LY8 cells (Figure 2A), indicating that transcriptional regulation did not contribute to SOX2 overexpression. Based on the RNA-seq data for the original and resistant cells, the interaction score from the KEGG pathway analysis showed that the PI3K/AKT signaling pathway and steroid biosynthesis were the most enriched pathways (Figure 2B and Supplementary Data 1). Furthermore, gene set enrichment analysis (GSEA) demonstrated that the gene signatures for the PI3K/AKT signaling pathway were significantly activated in both RCHO-resistant cell lines (Figure 2C and Supplementary Data 2). Experimentally, the result of flow cytometry also revealed that SOX2^+^ subpopulation exhibited higher AKT1 (S473) phosphorylation level than SOX2^-^ subpopulation in both RCHO-resistant LY8 and NU-DUL-1 cells (Figures S3A-C). Meanwhile, AKT1 (S473) phosphorylation was confirmed to remarkably increase in accordance with the resistance degree accompanied by the subsequently increased phosphorylation of its substrate PRAS40, thus enhancing SOX2 (T118) phosphorylation and subsequently suppressing SOX2 (K119) methylation in both cell lines. In addition, expression of the PI3K subunit p110α/δ and p85 in resistant LY8 cells and p110γ in resistant NU-DUL-1 cells were also elevated (Figure 2D). Consistently, for both GCB and ABC subtypes, AKT1 (S473) phosphorylation was also significantly increased in the relapsed biopsy tissues versus the paired patient tissues from the initial visit (Figure 2E and Figure S3D). The switch of SOX2 phosphorylation-methylation, which is regulated by PI3K/AKT1, was confirmed to regulate SOX2 stabilization via the ubiquitin system 11. Consistently, ubiquitinated-SOX2 was markedly reduced in resistant cells (Figure 2F). Further, ectopic expression of a constitutively-active form of myristoylated AKT1 in original LY8 and NU-DUL-1 cells enhanced the phosphorylation of AKT1 (S473) and PRAS40 (T246), and potently elevated SOX2 level, thus inducing the strong resistance to R and CHO treatment (Figures S3F-H). Therefore, the elevated SOX2 expression in resistant CSCs of GCB and ABC subtypes may result directly from the AKT1-regulated phosphorylation-methylation switch, further inhibiting SOX2 ubiquitination and degradation.

### Inhibition of PI3K/AKT1 but not of either upstream pathways effectively suppressed resistant cell survival during chemotherapy

**Figure 3:**
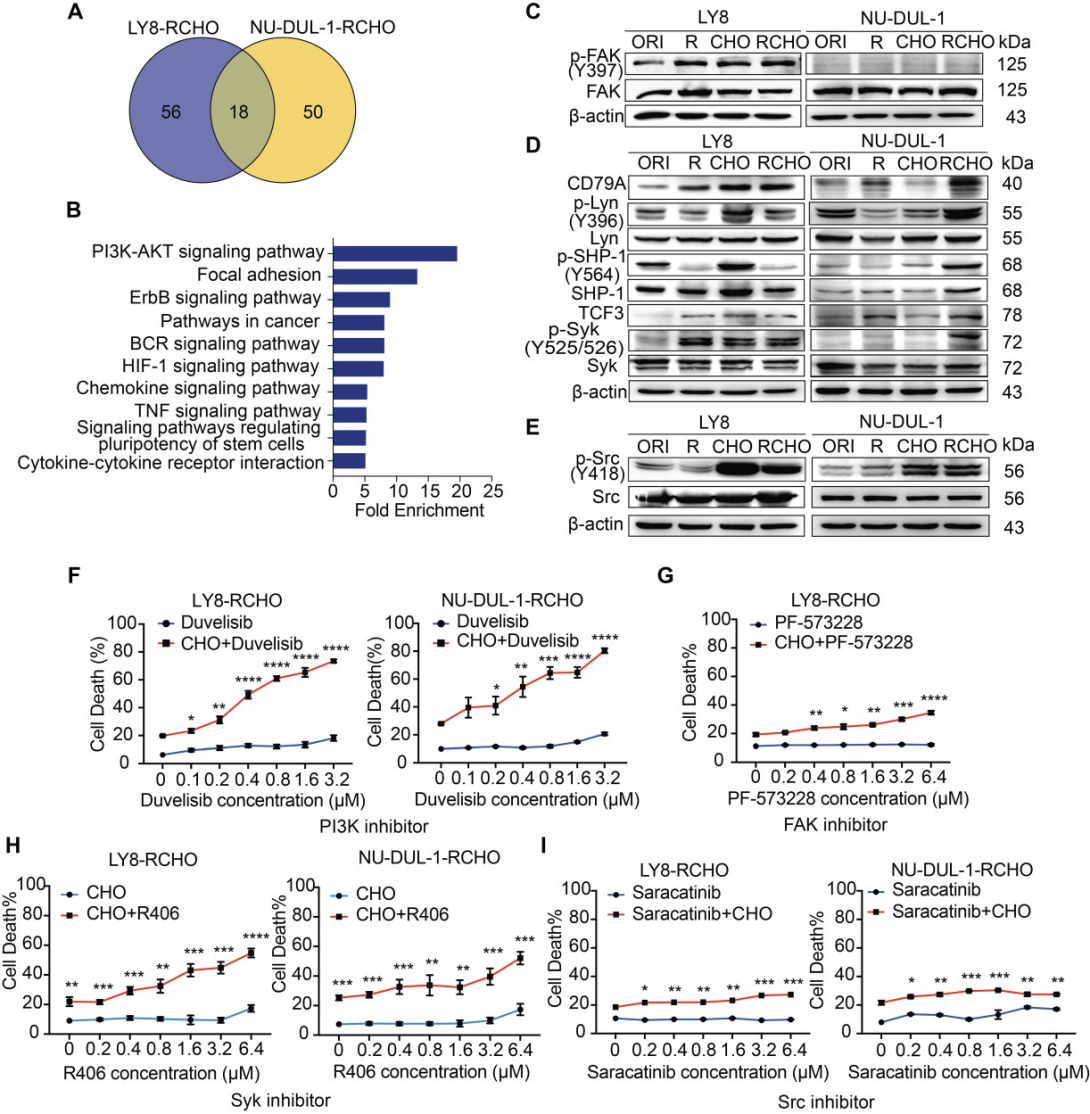
** Inhibition of PI3K/AKT1 but not of either upstream pathways effectively suppressed resistant cell survival during chemotherapy.** (**A**) Venn diagram illustrating the number of overlapping up-regulated genes in the PK3K/AKT1 pathway between RCHO-resistant LY8 and NU-DUL-1 cells. (**B**) The enriched top 10 signaling pathways based on the total 124 up-regulated genes in (A). (**C**) FAK was activated in resistant LY8 but not NU-DUL-1 cells. (**D**) Syk was activated in resistant DLBCL cells by BCR signaling pathway. (**E**) Src was activated in both CHO- and RCHO-resistant LY8 and NU-DUL-1 cells by chemokine signaling pathway. (**F**) CytoTox-Glo cytotoxicity assays: addition of the PI3K inhibitor duvelisib to CHO significantly reversed CHO resistance; however, duvelisib alone showed a negligible effect on direct induction of cell death. (**G**-**I**) Addition of FAK (G), Syk (H), or Src (I) inhibitors to CHO reversed resistance to CHO in the indicated RCHO-resistant DLBCL cells to a certain degree. Inhibitors alone exhibited a negligible cytotoxic effect on the induction of cell death. The cells were pretreated with duvelisib, PF-573228, R406, or saracatinib for 24 hours before addition of CHO to the medium. A CytoTox-Glo cytotoxicity assay was employed to measure the cytotoxic effect after treatment with CHO for 48 hours. The data are presented as mean ± SD; n=3. *P<0.05, **P<0.01, ***P<0.001 and ****P<0.0001 *vs* CHO alone treatment.

An understanding of the key regulators upstream of the PI3K/AKT pathway is helpful for identification of potential drug targets to reduce SOX2 expression. Thus, we determined the up-regulated genes in the PI3K/AKT pathway (Figure 2C and Supplementary Data 2) and classified them according to cell subtype (Figure 3A, Supplementary Data 3). Due to the limited number (18) of overlapping genes in the two cell subtypes, we used all 124 genes to perform the KEGG pathway analysis (Figure 3A). The top 10 pathways are listed in Figure 3B and Supplementary Data 4, and PI3K/AKT was convincingly ranked as number 1. PI3Kα/β isoforms are universally expressed, whereas PI3Kγ/δ are exclusively limited to hematopoietic cells, in which PI3Kδ plays a critical role in B cell development and function. Assuming that a number of potential upstream signalings may contribute to PI3K/AKT activation in concert (Figure 3B and Supplementary Data 4), we verified the reported activators, including BCR and other receptors to various integrins and cytokines/chemokines in lymphoma 29, 30. Consistently, we found that the integrin-regulated focal adhesion pathway was ranked number 2 (Figure 3B); however, downstream FAK was activated only in resistant LY8 cells (Figure 3C). The BCR signaling pathway was also involved in activating PI3K/AKT. Not only BCR/Lyn but also TCF3/SHP-1 signaling activated Syk in various resistant cells (Figure 3D). Moreover, we further investigated chemokine signaling pathway, which was suggested to activate the PI3K/AKT pathway (Figure 3B). The critical kinase downstream chemokine signaling pathway Src was activated in both CHO- and RCHO-resistant cells (Figure 3E). These results demonstrated that Focal adhesion, BCR and chemokine signaling pathways contributed to the activation of PI3K/AKT pathway in RCHO-resistant cells.

Next, we functionally tested the effect of inhibitors of the above signaling molecules on reversing resistance to chemotherapy (CHO) in resistant cells. Treatment with the PI3K inhibitor duvelisib alone induced marginal, if any, cytotoxicity; however, additive treatment with duvelisib and CHO dramatically enhanced the cytotoxic effect of CHO in both RCHO-resistant cell lines in a dose-dependent manner (Figure 3F). Similarly, treatment with a FAK, Syk, or Src inhibitor alone induced negligible cytotoxicity; however, combination treatment with CHO significantly enhanced the cytotoxic effect of CHO, but to a much smaller degree than the duvelisib combination (Figures 3G-I, Figure S4). The sensitizing effect of duvelisib resulted most likely from the fact that duvelisib in advance reduced the proportion of CSCs in RCHO-resistant cells determined by the sphere formation assay and ALDH1^+^ cell measurement (Figure S5). To summarize, the elevated PI3K/AKT1 signaling in resistant LY8 and NU-DUL-1 cells was induced by multiple factors, including focal adhesion, BCR and chemokine signaling, and inhibition of PI3K/AKT1 but not of either upstream pathways effectively suppressed resistant cell survival during chemotherapy, perhaps by reducing CSC proportion.

### GCB and ABC cells employed different SOX2-targeted signaling molecules to develop resistance

**Figure 4:**
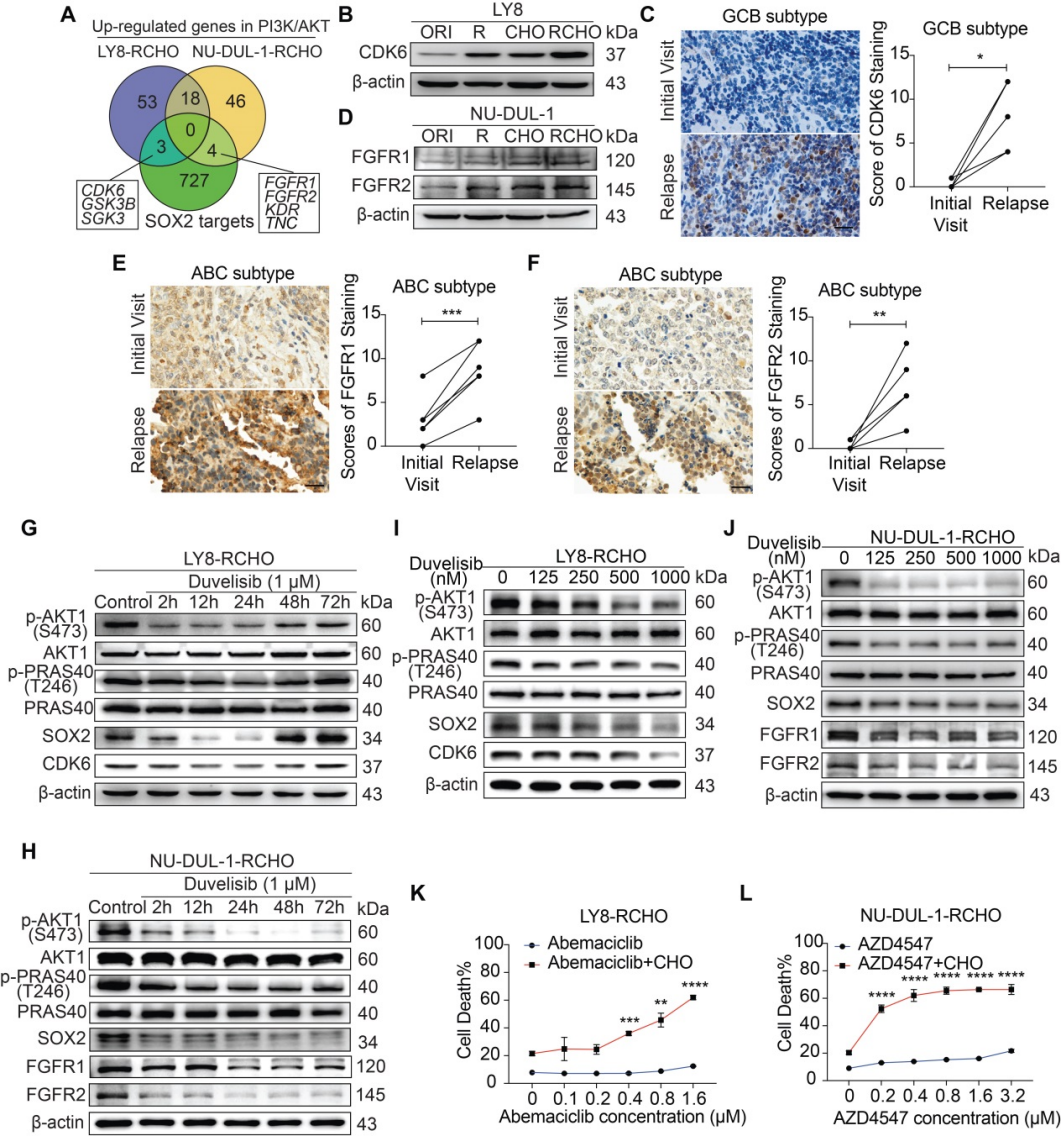
** Inhibition of CDK6 or FGFR1/2 restored sensitivity to CHO treatment.** (**A**) Venn diagram illustrating the number of overlapping up-regulated genes in the PK3K/AKT1 pathway and SOX2 targets. (**B**) CDK6 was overexpressed in resistant LY8 cells. (**C**) CDK6 was up-regulated in relapsed tissues compared with the paired tissues from the initial visit in 6 patients with the GCB subtype. (**D**) FGFR1 and FGFR2 were overexpressed in resistant NU-DUL-1 cells. (**E**-**F**) FGFR1 (E) and FGFR2 (F) was up-regulated in relapsed tissues compared with the paired tissue from the initial visit in 6 patients with the ABC subtype. Left: a representative image (C, E and F); Right: the quantitative scores for CDK6 (C), FGFR1 (E) and FGFR2 (F) staining. Scale bar: 20 μm.** (G**-**J)** Duvelisib reduced the expression of SOX2 and CDK6 (G and I) or FGFR1/2 (H and J) by suppressing AKT1 activity in RCHO-resistant LY8 (G and I) or NU-DUL-1 (H and J) cells, respectively, in a time- (G-H) and dose- (I-J) dependent manner. (**K**-**L**) Inhibition of CDK6 by abemaciclib in RCHO-resistant LY8 cells (K) or inhibition of FGFR1/2 by AZD4547 in RCHO-resistant NU-DUL-1 cells (L) restored sensitivity to CHO treatment. Abemaciclib or AZD4547 alone exhibited a negligible effect on induction of cell death. The cells were pretreated with abemaciclib or AZD4547 for 24 hours before addition of CHO to the medium. A CytoTox-Glo cytotoxicity assay was employed to measure the cytotoxic effect after treatment with CHO for 48 hours. The data are presented as mean ± SD; n=3. *P<0.05, **P<0.01, ***P<0.001, and ****P<0.0001.

Given the effect of PI3K/AKT inhibition or SOX2 insufficiency on reversing resistance to R and/or CHO treatment, we analyzed up-regulated genes in the PI3K/AKT pathway (Figure 2C and Supplementary Data 2) and SOX2-targeted genes (Molecular Signatures Database, MSigDB) to identify the critical targets of SOX2 in RCHO-resistant cells. Three genes, i.e., *CDK6*, *GSK3B*, and *SGK3*, were up-regulated in RCHO-resistant LY8 cells, while four genes, i.e., *FGFR1*, *FGFR2*, *KDR*, and *TNC*, were up-regulated in RCHO-resistant NU-DUL-1 cells (Figure 4A and Supplementary Data 5). Considering that CDK4/6 31 and FGFR 32 inhibitors are being tested in clinical trials for treatment of multi-type cancers, we subsequently investigated their expression and functions. CDK6 was highly expressed in resistant LY8 cells (Figure 4B) and relapsed GCB but not in ABC subtype DLBCL tissues (Figure 4C and Figures S6A-C). Consistently, FGFR1/2 were overexpressed in resistant NU-DUL-1 cells (Figure 4D) and relapsed ABC but not in GCB subtype DLBCL tissues (Figures 4E and F, and Figures S6D-I). Moreover, SOX2^+^ subpopulation expressed higher CDK6 or FGFR1/2 than SOX2^-^ subpopulation in RCHO-resistant LY8 or NU-DUL-1 cells, respectively (Figure S7), further indicating the close association between SOX2 and CDK6 or FGFR1/2. PI3K inhibition with duvelisib suppressed AKT1 and PRAS40 phosphorylation and induced SOX2 degradation, thus reducing CDK6 expression in LY8 RCHO-resistant cells (Figure 4G) or FGFR1/2 expression in NU-DUL-1 RCHO-resistant cells (Figure 4H) in a time dependent manner. Duvelisib effect on LY8 RCHO-resistant cells was more transient than that in NU-DUL-1 RCHO-resistant cells (Figures 4G and H), which resulted probably from the diverse metabolic features in different cell types. Duvelisib also displayed dose-effect relationship with above molecules (Figures 4I and J). Similar to the effect of PI3K inhibition, CDK6 inhibition with abemaciclib in RCHO-resistant LY8 cells or FGFR1/2 inhibition with AZD454 in RCHO-resistant NU-DUL-1 cells considerably enhanced the cytotoxicity of CHO, while these inhibitors alone displayed a negligible cytotoxic effect on the resistant cells (Figures 4K and L). These results indicated that inhibition of SOX2 targets (CDK6 in LY8-RCHO-resistant cells and FGFR1/2 in NU-DUL-1-RCHO-resistant cells) could at least partly reverse resistance to chemotherapy.

### PI3K/AKT Inhibition promoted CSCs differentiation by reducing SOX2

**Figure 5:**
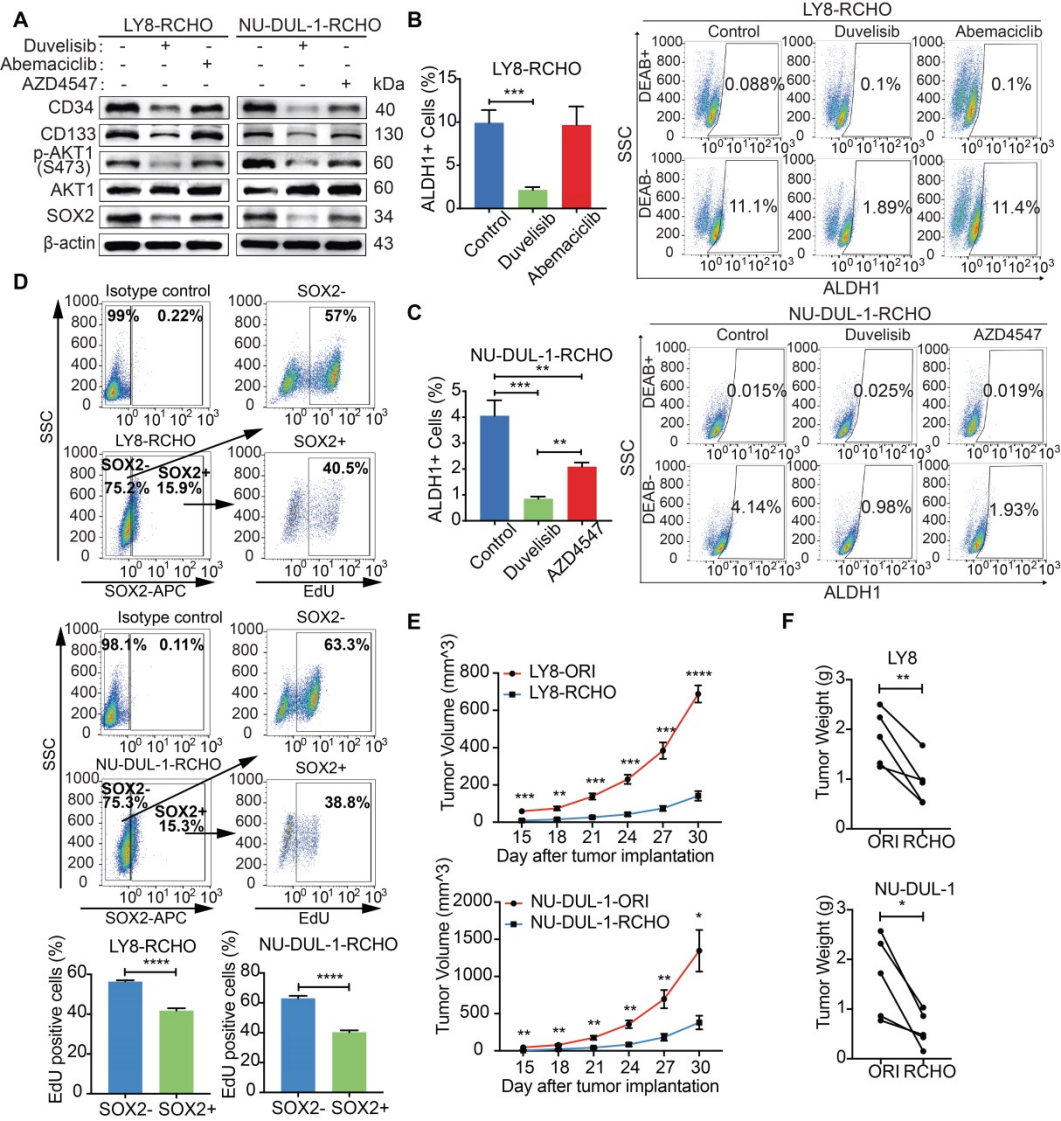
** PI3K/AKT inhibition promoted differentiation of resistant DLBCL cells by reducing SOX2 expression.** (**A**) Duvelisib clearly reduced the levels of CD34, CD133 in RCHO-resistant cells; abemaciclib exhibited no effect on their levels; while AZD4547 also reduced their levels, but to a greatly reduced degree compared with duvelisib. (**B**-**C**) Duvelisib dramatically reduced the ALDH1^+^ subpopulation in both RCHO-resistant LY8 (B) and NU-DUL-1 (C) cells. Abemaciclib had no effect on the ALDH1^+^ subpopulation in RCHO-resistant LY-8 cells (B), while AZD4547 significantly reduced this subpopulation in RCHO-resistant NU-DUL-1 cells, but to a greatly reduced extent compared with duvelisib (C). (**D**) SOX2^+^ subpopulation showed less EdU positive cells than SOX2^-^ population in RCHO-resistant LY8 (up) and NU-DUL-1 (down) cells detected by flow cytometry. Representative images (up) and quantitative results (bottom). The data are presented as mean ± SD; n=3. **P<0.01, ***P<0.001, and ****P<0.0001. (**E**-**F**) Both RCHO-resistant LY8 and NU-DUL-1 cells grew more slowly than the related original cells in the *in vivo* serial-transplantation experiment with 5x10^6^ tumor cells injection, in which both RCHO-resistant and original cells induced 100% tumor incidence (Table 1). Tumor growth rate (E) and paired tumor weight (F) at end-point of experiment. The data are presented as mean ± SEM; n=5. *P<0.05, **P<0.01, ***P<0.001, and ****P<0.0001.

We next investigated the role of PI3K/AKT/SOX2 axis in stemness maintenance. The expression levels of CD34, CD133 were clearly reduced after PI3K inhibition in both RCHO-resistant cell lines (Figure 5A), whereas CDK6 inhibition had no significant effect on the expression of the above molecules (Figure 5A). However, the expression levels of the above molecules also decreased after FGFR1/2 inhibition but to a smaller extent than those after PI3K inhibition (Figure 5A). In addition, PI3K inhibition significantly reduced the proportion of CSCs (marked as ALDH1^+^ cells) in both RCHO-resistant cell lines (Figures 5B and C); whereas CDK6 inhibition failed to change the proportion of CSCs in LY8 RCHO-resistant cells (Figure 5B). However, FGFR1/2 inhibition also significantly reduced the proportion of CSCs in NU-DUL-1 RCHO-resistant cells but to a much smaller degree than that with PI3K inhibition (Figure 5C), in agreement with a previous report showing that PI3K/AKT1 may be activated by FGFR signaling 33. Notably, FGFR1/2 were up-regulated and participated in PI3K/AKT1 signaling activation (Supplementary Data 3); thus, their inhibition reduced AKT1 phosphorylation and SOX2 stabilization (Figure 5A). These results revealed that PI3K/AKT1 inhibition directly by duvelisib or indirectly by AZD4547 promoted CSCs differentiation.

Differentiated tumor cells grow faster than CSCs. Indeed, SOX2^+^ subpopulation showed less EdU positive cells than SOX2^-^ subpopulation in RCHO-resistant LY8 and NU-DUL-1 cells (Figure 5D). Further, both RCHO-resistant cells grew more slowly than their related original cells in the *in vivo* serial-transplantation experiment with 5x10^6^ cells injection (Figures 5E and F), in which both RCHO-resistant and original cells induced 100% tumor incidence (Table 1 and Figure S2). PI3K/AKT inhibition effectively converted CSCs to differentiated cells, while CDK6 inhibition might directly suppress CSC growth through cell cycle targeting, and FGFR1/2 might have a dual effect on CSCs. All of these interventions eventually enhanced the CSC susceptibility to chemotherapy with varying efficacy.

### Pro-differentiation therapy against CSCs by combining a PI3K inhibitor with R-CHOP suppressed tumor growth of RCHO-resistant cells

**Figure 6:**
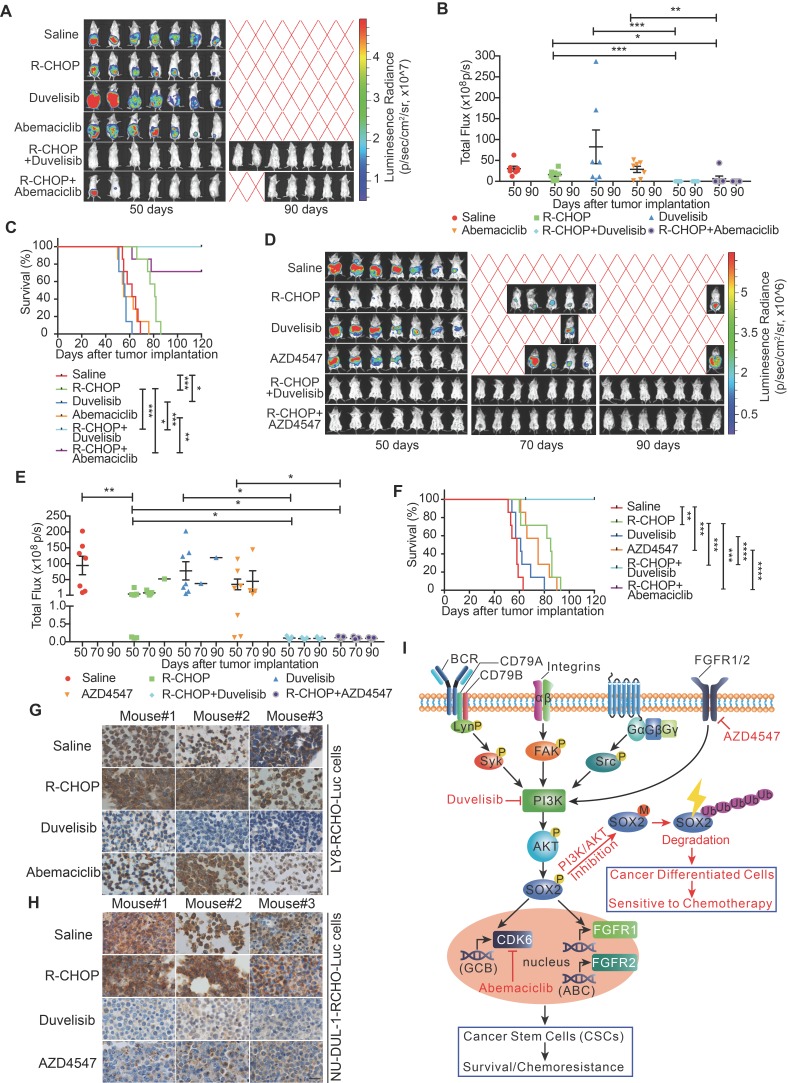
** The combination of PI3K-AKT1 or FGFR1/2 inhibitors but not of CDK6 inhibitor with R-CHOP suppressed the tumor growth of RCHO-resistant cells.** (**A**-**C**) Addition of abemaciclib and addition of duvelisib to R-CHO respectively, suppressed tumor growth of RCHO-resistant LY8 cells. (**D**-**F**) Addition of AZD4547 or duvelisib to R-CHOP effectively suppressed tumor growth of RCHO-resistant NU-DUL-1 cells. *In vivo* bioluminescence imaging (A and D). The tumor mass is represented by the quantified total photon flux (B and E). Kaplan-Meier survival curves (C and F). The red “X” in (A) and (D) represents mouse death. The data are presented as mean ± SEM (n=7). *P<0.05, **P<0.01, ***P<0.001, and ****P<0.0001. (**G**-**H**) SOX2 staining in LY8-RCHO-Luc (G) or NU-DUL-1-RCHO-Luc (H) cell-derived tumor tissues treated with the indicated drugs. Duvelisib and AZD4547, but not R-CHOP and abemaciclib, dramatically reduced the SOX2 expression level compared with the saline control. Scale bar, 20 μm. (**I**) Schematic showing the mechanisms by which pro-differentiation therapy against CSCs reverses drug resistance to R-CHOP in DLBCL.

RCHO-resistant LY8 and NU-DUL-1 cells transduced with luciferase-expressing plasmid were separately implanted into immuno-deficient mice, and various therapeutic regimens were administered. In mice bearing RCHO-resistant LY8 cells, R-CHOP treatment had a limited therapeutic effect on tumor growth without statistical significance compared with the saline control (Figures 6A and B) although it significantly prolonged the survival rate (Figure 6C). These results suggested that the* in vitro* resistance of LY8 cells to RCHO was recapitulated in mice to a high degree. Neither duvelisib nor abemaciclib alone suppressed tumor growth or prolonged the survival rate (Figures 6A-C); however, duvelisib accelerated tumor growth compared with saline, and although this difference was not statistically significant (Figures 6A and B), it led to a significantly shortened survival rate (Figure 6C). In contrast, when combined with R-CHOP, duvelisib or abemaciclib dramatically suppressed tumor growth and prolonged survival compared with R-CHOP or inhibitor alone (Figures 6A-C). More importantly, duvelisib combination therapy effectively suppressed tumorigenesis, and all the treated mice survived until the experimental end-point at day 120 compared with two mice receiving abemaciclib combination therapy that died on days 62 and 78 (Figures 6A-C). Similar but not identical results were obtained in mice bearing RCHO-resistant NU-DUL-1 cells. R-CHOP significantly suppressed tumor growth and prolonged survival (Figures 6D-F), likely due to the weaker stemness and reduced CSC proportions, resulting in weaker resistance than RCHO-resistant LY8 cells (Figures 1B-F). Duvelisib alone also had no therapeutic effect on tumor growth and survival, whereas AZD4547 alone significantly prolonged survival (Figures 6D-F). Importantly, when combined with R-CHOP, both duvelisib and AZD4547 effectively suppressed tumor growth and prolonged survival up to the experimental end-point of day 120 (Figures 6D-F). These different tumor-suppressing effects of duvelisib, abemaciclib and AZD4547 further support their potentially distinct resistance-reversing mechanisms; i.e., duvelisib converted CSCs to differentiated cells, abemaciclib inhibited CSC growth, and AZD4547 exhibited dual effects on CSCs. SOX2 staining of the implanted tumor tissues with different treatments supported this finding. Duvelisib or AZD4547, but not R-CHOP or abemaciclib, dramatically reduced SOX2 expression (Figures 6G and H). Therefore, the success of this combination therapy most likely resulted from CSCs conversion to differentiated cells via PI3K/AKT1/SOX2 axis inhibition.

## Discussion

A series of genetic events, which may have accumulated for years or decades, result in the activation or overexpression of genes facilitating tumor proliferation, and the silencing of genes inhibiting tumor growth, eventually leading to tumor initiation and progression 34, 35.Along with cancer progression, the differentiated phenotypes are gradually lost, and stem-like features are subsequently acquired by CSCs, resulting in metastasis and resistance to available therapies 36-38. However, the current attempts to reverse drug resistance by eradicating CSCs directly or slowing CSC growth indirectly have been far from success, including direct inhibition via CSC-dependent signaling (Wnt/β-catenin, Hedgehog, Notch, FAK, PTEN, Nanog, and JAK/STAT, among others) inhibitors, tumor microenvironment modulators, direct eradication via CD44-targeting or CD133-targeting immunotherapy, and differentiation therapy 39-42.

Herein, we provide a distinct strategy for targeting CSCs, termed pro-differentiation therapy. In RCHO-resistant DLBCL cells, BCR-mediated Syk, FAK, Src and FGFR1/2 signaling could activate PI3K/AKT1, which subsequently stabilized SOX2 by enhancing SOX2 phosphorylation and reducing SOX2 methylation. Eventually, the up-regulated SOX2 increased the survival and proportion of CSCs through CDK6 or FGFR1/2, depending on the resistant DLBCL cell subtype, thus inducing resistance. When combined with R-CHOP, PI3K/AKT1 signaling inhibitors effectively reversed drug resistance by accelerating SOX2 degradation, subsequently promoting CSC differentiation and the resultant hypersensitivity to chemotherapy (Figure 6I). Interestingly, using oncogenic dedifferentiation by machine learning in almost 12,000 samples of 33 tumor types, SOX2 has very recently been identified as a master stemness-associated transcription factor 43.

Given the critical role of CSCs in metastasis and drug resistance, we propose a novel strategy for coping with CSCs; i.e., identify the determinant signaling pathway for stemness maintenance and then guide CSCs to differentiate by interference with this pathway. The differentiated cells will eventually become sensitive to conventional therapies such as chemotherapy. In this case, a PI3K/AKT inhibitor regimen combined with R-CHOP merits assessment in clinical trials for resistant DLBCL patients. Even though this strategy is of translational importance, it is uncertain whether PI3K/AKT inhibitors could promote CSCs differentiation completely, thus whether this regimen would finally result in relapse still needs assessment in future clinical test.

## Conclusions

In the present study, we found that the CSC proportion was remarkably increased in R-CHOP resistant DLBCL cells. We further revealed that the PI3K-AKT-SOX2 axis plays a determinant role in the development of R-CHOP resistance in DLBCL cells by preventing ubiquitin-mediated SOX2 degradation, increasing SOX2 stability, thus maintaining the stemness of resistant DLBCL cells. Thus, a PI3K inhibitor duvelisib (in phase III clinical trial) converts CSCs of DLBCL to differentiated cells that are highly sensitive to R-CHOP regimen, which was termed pro-differentiation therapy against CSCs. Pro-differentiation therapy combining with R-CHOP could effectively reverse drug resistance and effectively suppress tumor growth of resistant DLBCL cells, thus curing DLBCL. Although PI3K/AKT inhibitors have been widely used to treat cancers, we provide a novel mechanism of them for promoting CSCs differentiation by destabilizing SOX2. Therefore, we hope this strategy may shed light on a real approach to CSCs-targeting therapy not only for DLBCL also for other types of cancers.

## Supplementary Material

Supplementary experimental procedures, tables, figures, and data.

## Figures and Tables

**Table 1:** RCHO-resistant DLBCL cells display higher tumor-initiating capability than original DLBCL cells.

Cell type	Cell dose	Tumor incidence	Tumor latency (weeks)
**LY8-ORI**	5 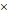 10^6^	5/5	5
	5 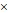 10^5^	0/5	-
	5 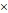 10^4^	0/5	-
	5 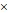 10^3^	0/5	-
	5 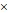 10^2^	0/5	-
**LY8-RCHO**	5 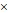 10^6^	5/5	5
	5 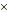 10^5^	5/5	5
	5 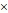 10^4^	4/5	7
	5 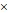 10^3^	3/5	8
	5 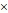 10^2^	2/5	10
**NU-DUL-1-ORI**	5 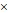 10^6^	5/5	4
	5 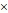 10^5^	1/5	5
	5 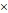 10^4^	0/5	-
	5 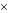 10^3^	0/5	-
	5 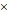 10^2^	0/5	-
**NU-DUL-1-RCHO**	5 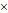 10^6^	5/5	4
	5 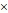 10^5^	5/5	5
	5 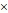 10^4^	3/5	6
	5 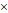 10^3^	3/5	7
	5 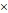 10^2^	2/5	10

## Abbreviations

DLBCL: diffuse large B cell lymphoma
R-CHOP: R=rituximab, C= cyclophosphamide, H= doxorubicin, O= vincristine, P= Prednisolone
CSC: cancer stem-like cell
GCB: germinal center B cell-like
ABC: activated B cell-like
BCR: B cell receptor
NHL: Non-Hodgkin's lymphoma
IHC: immunohistochemistry
ADCC: antibody-dependent cellular cytotoxicity
CDC: complement-dependent cytotoxicity
mCRPs: membrane-bound complement regulatory proteins
4-HC: 4-hydroxycyclophosphamide
NHS: normal human serum
GSEA: gene set enrichment analysis
