# Supplementary material for: PI3K/AKT inhibition reverses R-CHOP resistance by destabilizing SOX2 in diffuse large B cell lymphoma
Source: Theranostics. 2020 Feb 10;10(7):3151–63. doi: 10.7150/thno.41362 (PMC7053184; doi:10.7150/thno.41362)
Supplement: Supplementary file 1 — Supplementary experimental procedures, tables, figures, and data. [file thnov10p3151s1.pdf]

Inventory of Supplementary Information Items

ONLINE EXPERIMENTAL PROCEDURES

Supplemental References

Supplemental Figures and Legends

Supplementary data 1 for Figure 2B

Supplementary data 2 for Figure 2C

Supplementary data 3 for Figure 3A

Supplementary data 4 for Figure 3B

Supplementary data 5 for Figure 4A

ONLINE EXPERIMENTAL PROCEDURES

DLBCL tissue samples, cell lines and reagents

We examined the medical history of all DLBCL patients from 2008 to 2015 at Fudan University Shanghai Cancer Center and found a total of 12 patients who simultaneously had both paraffin-embedded tissue samples from the initial visit and from relapse. DLBCL cases were subgrouped into GCB (6 cases) or ABC (6 cases) molecular subtypes based on the Hans immunohistochemistry algorithm. The relapsed patients received R-CHOP therapy for at least 6 cycles. The first biopsy was performed for diagnosis at the initial visit, and the second biopsy was performed to detect the relapsed status or to confirm the primary diagnosis. The interval of the two biopsies differed from one another among the patients. These DLBCL samples were subsequently collected for IHC staining. The Ethics Committee at Fudan University Shanghai Cancer Center approved this study.

The human DLBCL cell lines GCB subtype OCI-LY8 and ABC subtype NU-DUL-1 were a kind gift from Professor Xiaoyan Zhou, Department of Pathology, Fudan University Shanghai Cancer Center (Shanghai, China). LY8 cells were maintained in Iscove's modified Dulbecco's medium supplemented with 10% fetal bovine serum and 1% penicillin/streptomycin. NU-DUL-1 cells were maintained in RPMI 1640 medium supplemented with 10% fetal bovine serum and 1% penicillin/streptomycin. The results of STR analysis for LY8 (OCI-Ly8) and NU-DUL-1 cell lines are showed in the

following table:

Results of cell line fingerprinting using STR.

Source	Sample Name	D5S818	D13S317	D7S820	D16S539	VWA	TH01	AM	TPOX	CSF1PO	Comment
Present study	OCI-LY8	10,13	11,11	11,12	9,11	17,19	6,9.3	X,X	9,11	11,12	MATCH
PMID: 23127183	OCI-LY8	10,13	11,11	11,12	9,11	17,19	6,9.3	X,X	9,11	11,12	
Present study	NU-DUL-1	12,14	8,13	11,13	11,13	16,17	9,9.3	X,X	8,8	11,12	MATCH
ATCC	NU-DUL-1	12,14	8,13	11,13	11,13	16,17	9,9.3	X,X	8,8	11,12	

As a complement source, normal human serum (NHS) was pooled from 10 healthy persons, aliquoted and stored at −80°C until use. Heat-inactivated human serum (IHS) as a negative control was prepared in a 65°C water bath for 30 min before use.

The anti-SOX2-T118p and anti-SOX2-K119me polyclonal antibodies were generated previously [1].

The PI3K inhibitor IPI-145 (duvelisib), CDK6 inhibitor abemaciclib, FGFR1/2 inhibitor AZD4547, FAK inhibitor PF-573228, Syk inhibitor R788, Src inhibitor saracatinib, Verapamil hydrochloride, Hoechst 33342 and prednisolone were purchased from MedChem Express (Monmouth Junction, NJ).

Generation and characterization of RCHO-resistant DLBCL cells

ADCC and CDC are the major mechanisms underlying R therapeutic effect [2, 3]. Resistance to ADCC may derive from intrinsic features of immune cells of individual patient, such as *FcGRIIIA* polymorphism and CD47 expression [4, 5]; while resistance to CDC is mediated by a low expression level of CD20 and/or high expression levels of mCRPs, including CD46, CD55 and especially CD59 [3, 6]. Therefore, we generated LY8 and NU-DUL-1 cells that were resistant to rituximab-mediated complement-dependent cytotoxicity (CDC), as previously described [7]. In brief, original LY8 (LY8-ORI) and NU-DUL-1 (NU-DUL-1-ORI) cells were treated with escalating rituximab (Roche, Basel, Switzerland) concentrations from 4 μg/mL to 32 μg/mL in the presence

of 20% NHS. The resistant cells were denoted LY8-R and NU-DUL-1-R. LY8-R and NU-DUL-1-R cells were treated with 32 μg/mL rituximab and 20% NHS every 21 days to maintain resistance. The cytolysis induced by CDC was assessed by detecting propidium iodide (PI) staining-positive cells with a fluorescence-activated cell sorting (FACS) assay.

CHO (cyclophosphamide/C, doxorubicin/H, vincristine/O) suppress tumor cell duplication by inducing DNA damage or binding to tubulin, and prodrug C must be metabolically activated *in vivo* into 4-HC to exert effect. Thus, chemo-resistant LY8 and NU-DUL-1 cells were generated as previously described[8]. Briefly, LY8-ORI and NU-DUL-1-ORI cells were treated with doxorubicin (Selleck Chemicals, Houston, TX) and vincristine (Selleck Chemicals, Houston, TX) at the clinical ratio of 50:1.4 by escalating the concentration. The maximum resistant dosage for LY8 cells was 125 ng/mL doxorubicin and 3.5 ng/mL vincristine, whereas it was 25 ng/mL doxorubicin and 0.7 ng/mL vincristine for NU-DUL-1 cells. These cells were then treated with 2 μg/mL 4-hydroperoxycyclophosphamide (4-HC) (Santa Cruz Biotechnology, Santa Cruz, CA) every 21 days for 3 cycles. The obtained CHO-resistant cells were termed LY8-CHO and NU-DUL-1-CHO, respectively. The CHO-resistant cells were treated with doxorubicin, vincristine and 4-HC every 21 days to maintain CHO resistance.

Using a similar approach, we treated the LY8-R and NU-DUL-1-R cells to generate LY8-RCHO-resistant cells and NU-DUL-1-RCHO-resistant cells. To maintain RCHO resistance, the LY8-RCHO and NU-DUL-1-RCHO cells were treated with doxorubicin, vincristine and 4-HC every 21 days following treatment with 32 μg/mL rituximab and 20% NHS. CHO and RCHO resistance were determined by serial CCK-8 assays after doxorubicin, vincristine and 4-HC treatment for 48 h.

Aldefluor Assay

ALDH1 is a selectable marker for multiple kinds of normal and cancer stem cells, including hematopoietic stem cells [9, 10]. Thus, we evaluated cancer stem-like cell numbers in hematopoietic malignancies by detecting ALDH1-positive cells. We employed an ALDEFLUOR™ kit (StemCell Technologies, Vancouver, BC, CA) to

detect populations with high ALDH1 activity as previously described. Briefly, 1×10^6 cells were suspended in 1 mL of assay buffer containing 1 μM ALDH1 substrate BAAA. The suspended cells were then equally divided into two aliquots, one as the negative control and the other as the test sample. To form the negative control, 50 mM DEAB, a specific ALDH inhibitor, was added to one aliquot. The cells were then incubated for 30 min at 37°C before flow cytometry analysis.

For concurrently detecting CD133 population and ALDH1 activity, the RCHO-resistant cells were incubated with Human TruStain FcX™ (Fc Receptor Blocking Solution) for 30 min at room temperature, then stained with anti-CD133 antibody for 30 min before the procedures of Aldefluor Assay.

Sphere Formation Assay

We conducted sphere formation assays as previously described [11]. In brief, cells were suspended in serum-free medium (DMEM/F12, 3:1 mixture) containing 0.4% BSA and 0.2× B27 lacking vitamin A (Life Technologies, Gaithersburg, MD) and supplemented with recombinant EGF (PeproTech, Rocky Hill, NJ) at 10 ng/mL, recombinant basic fibroblast growth factor (PeproTech, Rocky Hill, NJ) at 10 ng/mL and insulin (Sigma-Aldrich, St. Louis, MO) at 5 μg/mL. The cells were then seeded in ultra-low attachment 24-well plates at a density of 1×10^4 cells/mL. The medium was replaced every 7 days, and the spheres were counted and harvested at 14 days. The sphere cells were subcultured with the above medium at clonal density. Images were captured on the 14th day of cultivation after 3 passages.

CytoTox-Glo™ Cytotoxicity Assay

Cells were plated in 96-well plates at a density of 1×10^4 cells/100 μL/well. The cells were pretreated with IPI-145, abemaciclib, AZD4547, PF-573228, R788, or saracatinib for 24 h at escalating concentrations before addition of CHO to the medium. We used a CytoTox-Glo™ cytotoxicity assay kit (Promega, Madison, WI) to perform these assays according to the technical bulletin after treatment with CHO for 48 h. Briefly, 50 μL of CytoTox-Glo™ cytotoxicity assay reagent was added to all wells, mixed by orbital

shaking and then incubated for 15 min at room temperature. Experimental dead cell luminescence was measured with a Synergy HT microplate reader (BioTek, Biotek Winooski, MN), in which 50 μL of lysis reagent was added to all wells, mixed and incubated at room temperature for 15 min, followed by measurement of total luminescence. Cytotoxicity was calculated according to the following formula: Cytotoxicity (%) = Experimental dead cell luminescence/Total luminescence × 100%.

Immunohistochemistry

Tumor tissues derived from patients or animal models were fixed with 4% formalin, embedded in paraffin and sectioned. Paraffin sections were incubated with 3% hydrogen peroxide to block endogenous peroxidase for 15 min at 37°C and rinsed with 0.01 M PBS, followed by high-pressure antigen retrieval in EDTA buffer. The sections were then incubated with rabbit anti-SOX2 monoclonal antibody (1:200; Cell Signaling Technology, Danvers, MA) at 4°C overnight. After rinsing 3 times in PBS, the sections were incubated with peroxidase-conjugated AffiniPure goat anti-rabbit IgG H&L (1:200; Proteintech, Chicago, IL) at room temperature for 1 hour. Then, immunoreactivity was measured using a GTVision III immunohistochemical detection kit (GK500705; Gene Tech, Shanghai, China) according to the manufacturer's instructions. The immunostaining scores for SOX2, p-AKT, CDK6, FGFR1 and FGFR2 were assessed under a microscope by three independent individuals according to the following formula: score = 3 (strong positive) × percentage + 2 (moderate positive) × percentage + 1 (weak positive) × percentage + 0 (negative) × percentage [12].

Immunoblotting Assay

We performed immunoblotting assays according to the standard protocol, and the related antibodies are shown in the following table:

The commercial antibodies used in this study.

**Antibodies**	**Manufacturers**	**Applications in this study**	**Catalog Number**

CD34 [ICO-115]	abcam	WB (1:1,000)	ab187282
CD133	HuaBio Co., Ltd.	WB (1:1,000)	R121101
□-actin (C4)	Santa Cruz Biotechnology	WB (1:1,000)	sc-47778
Sox2 (D6D9) XP Rabbit mAb	Cell Signaling Technology	WB (1:1,000), IHC (1:200)	3579
OCT4 [EPR17929]	abcam	WB (1:1,000)	ab181557
Nanog [EPR2027(2)]	abcam	WB (1:1,000)	ab109250
KLF4 [EPR19590]	abcam	WB (1:1,000)	ab215036
c-Myc (D3N8F) Rabbit mAb	Cell Signaling Technology	WB (1:1,000)	13987
Phospho-Akt (Ser473) (D9E)	Cell Signaling Technology	IHC (1:100) WB (1:1,000)	4060
PI3 Kinase p110α (C73F8)	Cell Signaling Technology	WB (1:1,000)	4249
PI3 Kinase p110β (C33D4)	Cell Signaling Technology	WB (1:1,000)	3011
PI3 Kinase p110γ (D55D5)	Cell Signaling Technology	WB (1:1,000)	5405
PI3 Kinase p110 δ (D1Q7R)	Cell Signaling Technology	WB (1:1,000)	34050
PI3 Kinase p85 (19H8)	Cell Signaling Technology	WB (1:1,000)	4257
Akt1 (C73H10)	Cell Signaling Technology	WB (1:1,000)	2938
Ubiquitin (P4D1)	Cell Signaling Technology	WB (1:1,000)	3936

Phospho-FAK (Tyr397) (D20B1)	Cell Signaling Technology	WB (1:1,000)	8556
FAK (D2R2E)	Cell Signaling Technology	WB (1:1,000)	13009
Phospho-Akt1 (Ser473) (D7F10)	Cell Signaling Technology	WB (1:1,000)	9018
CD79a [EPR3619]	abcam	WB (1:1,000)	ab133483
Phospho-Syk (Tyr525/526) (C87C1)	Cell Signaling Technology	WB (1:1,000)	2710
Syk (N-19)	Santa Cruz Biotechnology	WB (1:500)	sc-1077
p-Src (Y418) [EP503Y]	abcam	WB (1:1,000)	ab40660
Src [EPR5496]	abcam	WB (1:1,000)	ab109381
CDK6 [EPR4515]	abcam	WB (1:1,000)<br>IHC (1:100)	ab124821
FGFR1	abcam	WB (1:1,000)<br>IHC (1:400)	ab10646
FGFR2 (C17)	Santa Cruz Biotechnology	WB (1:1,000)<br>IHC (1:200)	sc-122
APC anti-human CD133	BioLegend	FACS (5 µL/test)	372806
Human TruStain FcX^{TM} (Fc Receptor Blocking Solution)	BioLegend	FACS (5 µL/test)	422301
FITC Goat anti-mouse IgG (minimal x-reactivity)	BioLegend	FACS (1 µg/test)	405305
PE Donkey anti-rabbit IgG (minimal x-reactivity)	BioLegend	FACS (0.125 µg/test)	406421
APC Goat anti-mouse IgG (minimal x-reactivity)	BioLegend	FACS (0.125 µg/test)	405308

Phospho-PRAS40 (Thr246) (C77D7) Rabbit mAb	Cell Signaling Technology	WB (1:1,000)	2997
PRAS40 (D23C7) XP® Rabbit mAb	Cell Signaling Technology	WB (1:1,000)	2691
Sox2 (D9B8N) Rabbit mAb	Cell Signaling Technology	IP (1:100)<br>FACS (1:100)	23064
Sox2 (L1D6A2) Mouse mAb	Cell Signaling Technology	FACS (1:200)	4900
Goat anti-mouse IgG-HRP	Santa Cruz Biotechnology	WB (1:10,000)	sc-2005th
Goat anti-rabbit IgG-HRP	Santa Cruz Biotechnology	WB (1:10,000)	sc-2004
Peroxidase-conjugated affinipure goat anti-rabbit IgG H&L	Proteintech Group Inc.	IHC (1:200)	SA00001-2
Normal mouse IgG	Santa Cruz Biotechnology	2 µg/test	sc-2025

Quantitative Real-time PCR (qRT-PCR)

We performed qRT-PCR as previously described [11]. Briefly, total RNA from cells was extracted with TRIzol reagent (Invitrogen, Grand Island, NY) and then transcribed into cDNA using a Reverse Transcription System (Promega, Madison, WI). The input cDNA was standardized and amplified for 40 cycles with SYBR Green Master Mix (Invitrogen, Grand Island, NY) and gene-specific primers on a Roche LightCycler 480 system (Roche, Basel, Switzerland). We used the *ACTB* gene encoding β-actin as the endogenous control, and the samples were analyzed in triplicate. The primers for qRT-PCR are listed in the following table:

The sequences of primers for qRT-PCR.

**Primers**	**5' to 3'**

SOX2 qRT-PCR forward primer	GCCGAGTGGAAACTTTTGTCG
SOX2 qRT-PCR reverse primer	GGCAGCGTGTACTTATCCTTCT

FACS Analysis

After washing with PBS, cells were incubated with fluorescein-conjugated antibodies for 30 min and then rinsed and resuspended in PBS. Fluorochrome-conjugated isotype-specific IgGs served as controls. Flow cytometric analysis was performed on a Cytomics FC500 MPL instrument (Beckman Coulter, Brea, CA) and analyzed with FlowJo software (Ashland, OR). We performed cell sorting with a MoFlo XDP instrument (Beckman Coulter, Brea, CA) according to the relative fluorescence.

For concurrently detecting SOX2 and FGFR1/2, the RCHO-resistant cells were stained with anti-FGFR1 or anti-FGFR2 antibody for 30 min before using the True-Nuclear™ Transcription Factor Buffer Set (BioLegend, San Diego, CA) for fixation and permeabilization. Then the cells were stained with anti-SOX2 antibody for 30 min and the related secondary antibodies conjugated with different fluorescence.

For concurrently detecting SOX2 and p-AKT1 (S473), CDK6, the RCHO-resistant cells were fixed and permeabilized using the True-Nuclear™ Transcription Factor Buffer Set (BioLegend, San Diego, CA). Then the cells were stained with anti-SOX2 and anti-p-AKT1 (S473) or anti-CDK6 antibodies for 30 min, subsequently with related secondary antibodies conjugated with different fluorescence.

Side Population Assay

The side population (SP) of the original and resistant DLBCL cells were identified using the DNA-binding dye Hoechst 33342 (MedChem Express, Monmouth Junction, NJ) and a MoFlo XDP instrument (Beckman Coulter, Brea, CA) based on the protocol described by Goodell et al [13]. Briefly, a total of 5 x 10^7 cells were harvested by centrifugation, and re-suspended with pre-warmed DMEM medium containing 2% FBS. Then the cells were stained with Hoechst 33342 in a final concentration of 5 μg/mL in the 37°C water bath in the dark, either alone or in the present of 100 μM Verapamil

hydrochloride (MedChem Express, Monmouth Junction, NJ). After 2 h incubation, the cells were centrifugated and re-suspended with pre-cold HBSS buffer containing 2% FBS and 10mM Hepes. Finally, 1 μg/mL propidium iodine (PI) (Sigma-Aldrich, St. Louis, MO) was added for the discrimination of dead cells. The cells were maintained on the ice until analysis.

EdU Cell Proliferation Assay

For EdU assays on SOX2-positive cells, we used the True-Nuclear™ Transcription Factor Buffer Set (BioLegend, San Diego, CA) for fixation and permeabilization. The RCHO-resistant cells were subsequently stained with anti-SOX2 antibody conjugated with APC fluorescence for 30 min. Then EdU proliferation assay were conducted according to the manufacturer's instructions of the Cell-Light EdU Apollo488 In Vitro Flow Cytometry Kit (Guangzhou RiboBio Co., Ltd., Guangzhou, China). Next, we assessed EdU staining-positive cells on a Cytomics FC500 MPL instrument (Beckman Coulter, Brea, CA).

Plasmid Construction and Lentiviral Transduction

The coding DNA sequence (CDS) of human *SOX2* was obtained by PCR amplification from cDNA pools of A549 cells as previously described [11]. This sequence was cloned into the pCDH cDNA cloning and expression lentivector (cat# CD511B-1, System Biosciences, Palo Alto, CA 94303) via EcoRI and BamHI endonuclease sites for stable *SOX2* overexpression in OCI-LY8 and NU-DUL-1 cells. The CDS of the *Firefly Luciferase* gene was obtained by PCR amplification from the pGL3-Basic plasmid and inserted into the pCDH cDNA cloning and expression lentivector via EcoRI and BamHI endonuclease sites. The pLKO.3G cloning vector (plasmid #14748, Addgene, Cambridge, MA) was employed to construct scramble (SCR), *SOX2* shRNA plasmids. 293FT cells were co-transfected with the pCDH or pLKO.3G plasmid and pMD.2G and psPAX2 plasmids to generate *SOX2*, *firefly luciferase* overexpression or *SOX2* knockdown lentivirus, respectively. The lentivirus was subsequently added to OCI-LY8, NU-DUL-1, LY8-RCHO or NU-DUL-1-RCHO culture medium for 48 h of incubation.

All the cells transduced with lentivirus in this study were sorted by GFP with a MoFlo XDP instrument (Beckman Coulter, Brea, CA). Information regarding the primers and shRNA oligonucleotide sequences is shown in the following table:

The sequences of primers and shRNA for plasmid construction in this study.

Firefly Luciferase CDS forward primer	ATGGAAGACGCCAAAAACATAAAG
Firefly Luciferase CDS reverse primer	TTACACGGCGATCTTTCCGCCCTT
SOX2 shRNA#1 forward primer	AATTAGGAGCACCCGGATTATAAATCTCGAGATTT ATAATCCGGGTGCTCCTTTTTTTAT
SOX2 shRNA#1 reverse primer	AAAAAAAAGGAGCACCCGGATTATAAATCTCGAGA TTTATAATCC GGGTGCTCCT
SOX2 shRNA#2 forward primer	AATTGGTTGACACCGTTGGTAATTTCTCGAGAAAT ACCAACGGTGTCAACCTTTTTTTAT
SOX2 shRNA#2 reverse primer	AAAAAAAGGTTGACACCGTTGGTAATTTCTCGAGA AATTACCAACGGTGTCAACC
scramble shRNA forward primer	AATTCCTAAGGTTAAGTCGCCCTCGCTCGAGCGAG GGCGACTTAACCTTAGGTTTTTT
scramble shRNA reverse primer	AAAAAAACCTAAGGTTAAGTCGCCCTCGCTCGAGC GAGGGCGACTTAACCTTAGG

Xenograft Model

All the animal experiments were conducted in strict accordance with experimental protocols approved by the Animal Ethics Committee at Shanghai Medical School, Fudan University.

To compare the tumor-initiating capacity of RCHO-resistant and original cells, six-week-old male NOD-SCID mice were purchased from Slac Laboratory Animal Center (Shanghai, China). Then, two pairs of cells, LY8-ORI and LY8-RCHO cells, and NU-DUL-1-ORI and NU-DUL-1-RCHO cells, at the dose of $5x10^2$, $5x10^3$, $5x10^4$, $5x10^5$,

or $5x10^6$ cells were re-suspended in a PBS/Matrigel (Invitrogen, Grand Island, NY) mixture (1:1 volume). Further, each dose of RCHO-resistant or original cells were injected subcutaneously into bilateral backside, respectively, in same mouse, 5 mice in for each dose. The mice were monitored for tumor volume and incidence. The tumor volume was determined following a standard formula: Length×Width^2/2. The tumor-free mice were followed up until 80 days after implantation.

To test the drug efficacy on suppressing tumor growth, we transduced the LY8-RCHO and NU-DUL-1-RCHO cells with lentivirus overexpressing the *firefly luciferase* gene to generate stable cells expressing firefly luciferase, termed LY8-RCHO-Luc and NU-DUL-1-RCHO-Luc cells, respectively. Eight-week-old female SCID mice were purchased from Slac Laboratory Animal Center (Shanghai, China). LY8-RCHO-Luc and NU-DUL-1-RCHO-Luc cells were resuspended in PBS and then intraperitoneally injected at 1.5×10^7 cells per mouse. The mice were randomly divided into 6 groups (7 mice per group) after intraperitoneal injection with LY8-RCHO-Luc cells and administered saline, R-CHOP, duvelisib, abemaciclib, R-CHOP+duvelisib, or R-CHOP+abemaciclib. The mice bearing NU-DUL-1-RCHO-Luc cells were also randomly divided into 6 groups (7 mice per group), which were administered saline, R-CHOP, duvelisib, AZD4547, R-CHOP+duvelisib, or R-CHOP+AZD4547. The R-CHOP regimen was employed in clinical use for DLBCL therapy [14], and duvelisib, abemaciclib, and AZD4547 were tested in clinical trials (the related NCT numbers are NCT02576275, NCT01739309, and NCT01739309, respectively). For the first time of drug delivery, duvelisib, abemaciclib and AZD4547 were administrated 8 h before R-CHOP delivery. Tumor growth was monitored by bioluminescence at 50 and 90 days after implantation. For in vivo luminescence imaging, D-luciferin (Promega, Madison, WI) was injected intraperitoneally into the mice (150 mg/kg). After 10 min, the mice were anesthetized by intraperitoneal injection with pentobarbital (50 mg/kg), and then bioluminescence was examined using an In Vivo MS FX PRO system (Bruker, Billerica, MA). Luminescence images were captured with 30 s of exposure time, and the signal intensities of the tumors were measured using Bruker MI software. The survival time of each mouse was recorded. The surviving mice were euthanized by CO_2 and dissected

at 120 days after xenografting, and no intraperitoneal tumors were found. Tumor tissues were collected from the moribund mice euthanized by CO_2 and fixed with 4% formalin.

The methods of drug delivery based on the clinical usage for one cycle are indicated in the following table:

Methods of drug delivery in xenograft model.

**Drug**	**Dosage (mg/kg)**	**Administration Day (Days after tumor implantation)**	**Administration Route**
Rituximab (R)	118.4	8	intraperitoneal injection
Cyclophosphamide (C)	235.6	8	intraperitoneal injection
Doxorubicin (H)	16	8	intraperitoneal injection
Vincristine (O)	0.444	8	intraperitoneal injection
Prednisolone (P)	12.3	8, 9, 10, 11, 12	intraperitoneal injection
Duvelisib	5.18	8, 10, 12, 13, 15, 17, 19, 21, 23, 25, 27	gavage
Abemaciclib	41.1	8, 10, 12, 13, 15, 17, 19, 21, 23, 25, 27	gavage
AZD4547	32.8	8, 9, 10, 11, 12, 13, 14, 15, 16, 17, 18, 19, 20, 21	gavage

RNA Sequencing and Bioinformatic Analysis

Total RNA was extracted from LY8-ORI, LY8-R, LY8-CHO, LY8-RCHO, NU-DUL-1, NU-DUL-1-R, NU-DUL-1-CHO, and NU-DUL-1-RCHO cells with TRIzol reagent (Invitrogen, Grand Island, NY). The total RNA from each group from 3 different passages was pooled separately. RNA sequencing (RNA-seq) and bioinformatics analysis were conducted by Shanghai Novelbio Ltd. according to their established procedures [15]. We applied the DEseq algorithm to filter the differentially expressed genes after significance and false discovery rate (FDR) analyses under the following

criteria: (1) fold change >1.5 or <0.667; (2) FDR<0.05. Pathway analysis was used to identify significant pathways of the differential genes according to the KEGG database. Series-cluster analysis was performed to identify global trends and model profiles of expression according to the signal density of the ORI-R-RCHO (A series in Fig. 2B) and ORI-CHO-RCHO (B series in Fig. 2B) sequences. Fisher's exact test and the multiple comparison test were used to select significant pathways, and the threshold of significance was defined by *P*-value and FDR [16, 17]. We screened significantly enriched pathways with escalating trends in ORI-R-RCHO and ORI-CHO-RCHO sequences and then analyzed the interaction frequency of these pathways between these groups using Cytoscape software [18]. The interaction network illustrated enriched pathways with escalating trend, and the pathway interacts with more nodes of series is of higher score.

We performed gene set enrichment analysis (GSEA) to identify functions of differentially expressed genes based on RNA-seq using GSEA software from the Broad Institute (Massachusetts Institute of Technology) [19, 20]. The pre-ranked version of the software was used to identify significantly enriched pathways, and pathways enriched with FDR<0.25 were considered significant. The PI3K-AKT signaling pathway gene set used in this study consisted of 341 genes from the PI3K-AKT signaling pathway SuperPath in the PathCards pathway unification database (Version 4.6.0.37, Weizmann Institute of Science). The GSEA term KEGG_CHEMOKINE_SIGNALING_PATHWAY was also used to identify the chemokine signaling pathway.

The RNA-seq data reported in this study are accessible in the NCBI GEO database through GEO series accession numbers GSE112989 and GSE113001.

Statistical Methods

The data are presented as mean ± SD unless otherwise specified. Significant differences between two groups were determined using two-tailed Student's t-test for unpaired data, and $P<0.05$ was considered statistically significant. For IHC staining scores of tissue microarrays, significance was determined by two-tailed paired t-test, and $P<0.05$ was

considered statistically significant. For the total photon flux of animal models, significance was determined by a one-tailed Mann-Whitney test, and $P<0.05$ was considered statistically significant. We applied a Mantel-Cox test to compare the survival rates between the two groups of xenograft models, and $P<0.05$ was considered statistically significant. All statistical analyses were performed by GraphPad Prism 7 (La Jolla, CA).

References

1. Fang L, Zhang L, Wei W, Jin X, Wang P, Tong Y, et al. A methylation-phosphorylation switch determines Sox2 stability and function in ESC maintenance or differentiation. Molecular cell. 2014; 55: 537-51.
2. Smith MR. Rituximab (monoclonal anti-CD20 antibody): mechanisms of action and resistance. Oncogene. 2003; 22: 7359-68.
3. Zhou X, Hu W, Qin X. The role of complement in the mechanism of action of rituximab for B-cell lymphoma: implications for therapy. Oncologist. 2008; 13: 954-66.
4. Cartron G, Dacheux L, Salles G, Solal-Celigny P, Bardos P, Colombat P, et al. Therapeutic activity of humanized anti-CD20 monoclonal antibody and polymorphism in IgG Fc receptor FcgammaRIIIa gene. Blood. 2002; 99: 754-8.
5. Russ A, Hua AB, Montfort WR, Rahman B, Riaz IB, Khalid MU, et al. Blocking "don't eat me" signal of CD47-SIRPalpha in hematological malignancies, an in-depth review. Blood Rev. 2018.
6. Fishelson Z. Obstacles to cancer immunotherapy: expression of membrane complement regulatory proteins (mCRPs) in tumors. Molecular Immunology. 2003; 40: 109-23.
7. Hu W, Ge X, You T, Xu T, Zhang J, Wu G, et al. Human CD59 inhibitor sensitizes rituximab-resistant lymphoma cells to complement-mediated cytolysis. Cancer Res. 2011; 71: 2298-307.
8. Maxwell SA, Cherry EM, Bayless KJ. Akt, 14-3-3zeta, and vimentin mediate a drug-resistant invasive phenotype in diffuse large B-cell lymphoma. Leukemia & lymphoma. 2011; 52: 849-64.
9. Ginestier C, Hur MH, Charafe-Jauffret E, Monville F, Dutcher J, Brown M, et al. ALDH1 is a marker of normal and malignant human mammary stem cells and a predictor of poor clinical outcome. Cell stem cell. 2007; 1: 555-67.
10. Chute JP, Muramoto GG, Whitesides J, Colvin M, Safi R, Chao NJ, et al. Inhibition of aldehyde dehydrogenase and retinoid signaling induces the expansion of human hematopoietic stem cells. Proc Natl Acad Sci U S A. 2006; 103: 11707-12.
11. Chen J, Ding P, Li L, Gu H, Zhang X, Zhang L, et al. CD59 Regulation by SOX2 Is Required for Epithelial Cancer Stem Cells to Evade Complement Surveillance. Stem Cell Reports. 2017; 8: 140-51.
12. Zhang X, Zhang L, Du Y, Zheng H, Zhang P, Sun Y, et al. A novel FOXM1 isoform,

FOXM1D, promotes epithelial-mesenchymal transition and metastasis through ROCKs activation in colorectal cancer. Oncogene. 2017; 36: 807-19.
13. Goodell MA, Brose K, Paradis G, Conner AS, Mulligan RC. Isolation and functional properties of murine hematopoietic stem cells that are replicating in vivo. J Exp Med. 1996; 183: 1797-806.
14. Cunningham D, Hawkes EA, Jack A, Qian W, Smith P, Mouncey P, et al. Rituximab plus cyclophosphamide, doxorubicin, vincristine, and prednisolone in patients with newly diagnosed diffuse large B-cell non-Hodgkin lymphoma: a phase 3 comparison of dose intensification with 14-day versus 21-day cycles. Lancet. 2013; 381: 1817-26.
15. Liu Z, Li X, Zhang JT, Cai YJ, Cheng TL, Cheng C, et al. Autism-like behaviours and germline transmission in transgenic monkeys overexpressing MeCP2. Nature. 2016; 530: 98-102.
16. Ramoni MF, Sebastiani P, Kohane IS. Cluster analysis of gene expression dynamics. Proc Natl Acad Sci U S A. 2002; 99: 9121-6.
17. Miller LD, Long PM, Wong L, Mukherjee S, McShane LM, Liu ET. Optimal gene expression analysis by microarrays. Cancer Cell. 2002; 2: 353-61.
18. Shannon P, Markiel A, Ozier O, Baliga NS, Wang JT, Ramage D, et al. Cytoscape: a software environment for integrated models of biomolecular interaction networks. Genome Res. 2003; 13: 2498-504.
19. Mootha VK, Lindgren CM, Eriksson KF, Subramanian A, Sihag S, Lehar J, et al. PGC-1alpha-responsive genes involved in oxidative phosphorylation are coordinately downregulated in human diabetes. Nat Genet. 2003; 34: 267-73.
20. Subramanian A, Tamayo P, Mootha VK, Mukherjee S, Ebert BL, Gillette MA, et al. Gene set enrichment analysis: a knowledge-based approach for interpreting genome-wide expression profiles. Proc Natl Acad Sci U S A. 2005; 102: 15545-50.

Supplemental Figures and Legends

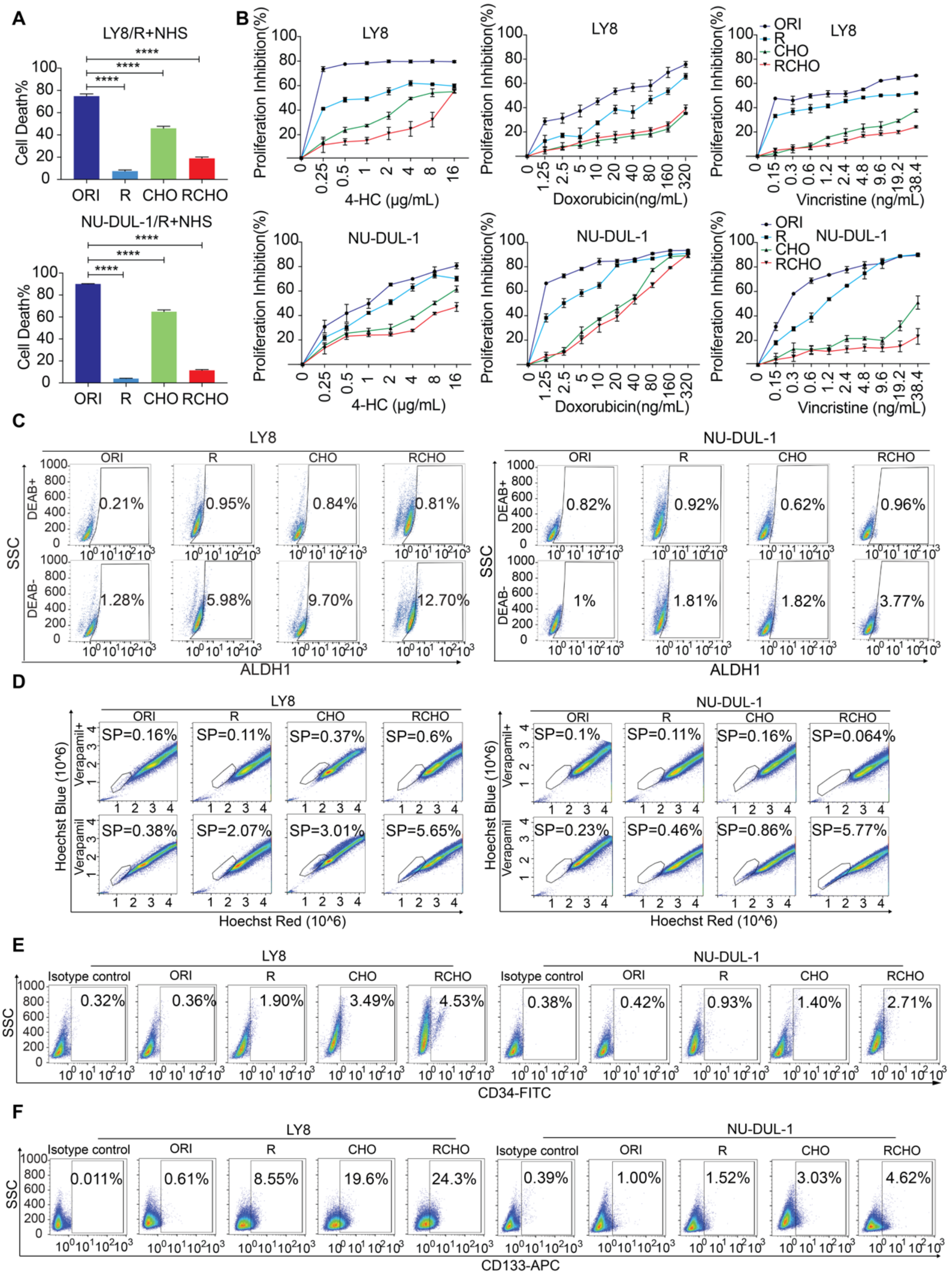

Figure S1. Resistance validation and stemness characterization of the resistant DLBCL cells/tissues. (**A and B**) Resistance to R-mediated CDC (A) or chemotherapy (B) was confirmed by a CDC assay (A) or CCK-8 assay (B), respectively, in which the cells were treated with 3.2 μg/mL rituximab and 20% NHS for 2 h (A) or by escalating

dosages of 4-HC, doxorubicin, or vincristine for 48 h (B). (**C**) Representative images of the Aldefluor assay. The percentage of ALDH1-positive cells in resistant DLBCL cells was gradually enhanced in order of R-, CHO- and RCHO-resistant cells compared with the original cells, indicating increased stemness of the resistant DLBCL cells. The related quantitative results are shown in Figure 1C by subtracting the DEAB controls. (**D**) Representative images of the side population assay. The percentage of side population fraction in resistant DLBCL cells was gradually increased in order of R-, CHO- and RCHO-resistant cells compared with the original cells. The related quantitative results are shown in Figure 1D by subtracting the Verapamil control. (**E and F**) Representative images of CD34 and CD133 detection by flow cytometry. Fluorochrome-conjugated isotype-specific IgGs were used to set the gate for CD34- or CD133-positive cells. The percentage of CD34- and CD133-positive cells in resistant DLBCL cells was gradually increased in order of R-, CHO- and RCHO-resistant cells compared with the original cells. The related quantitative results are shown in Figure 1E and F. The data are presented as mean ± SD; n=3. ****P<0.0001.

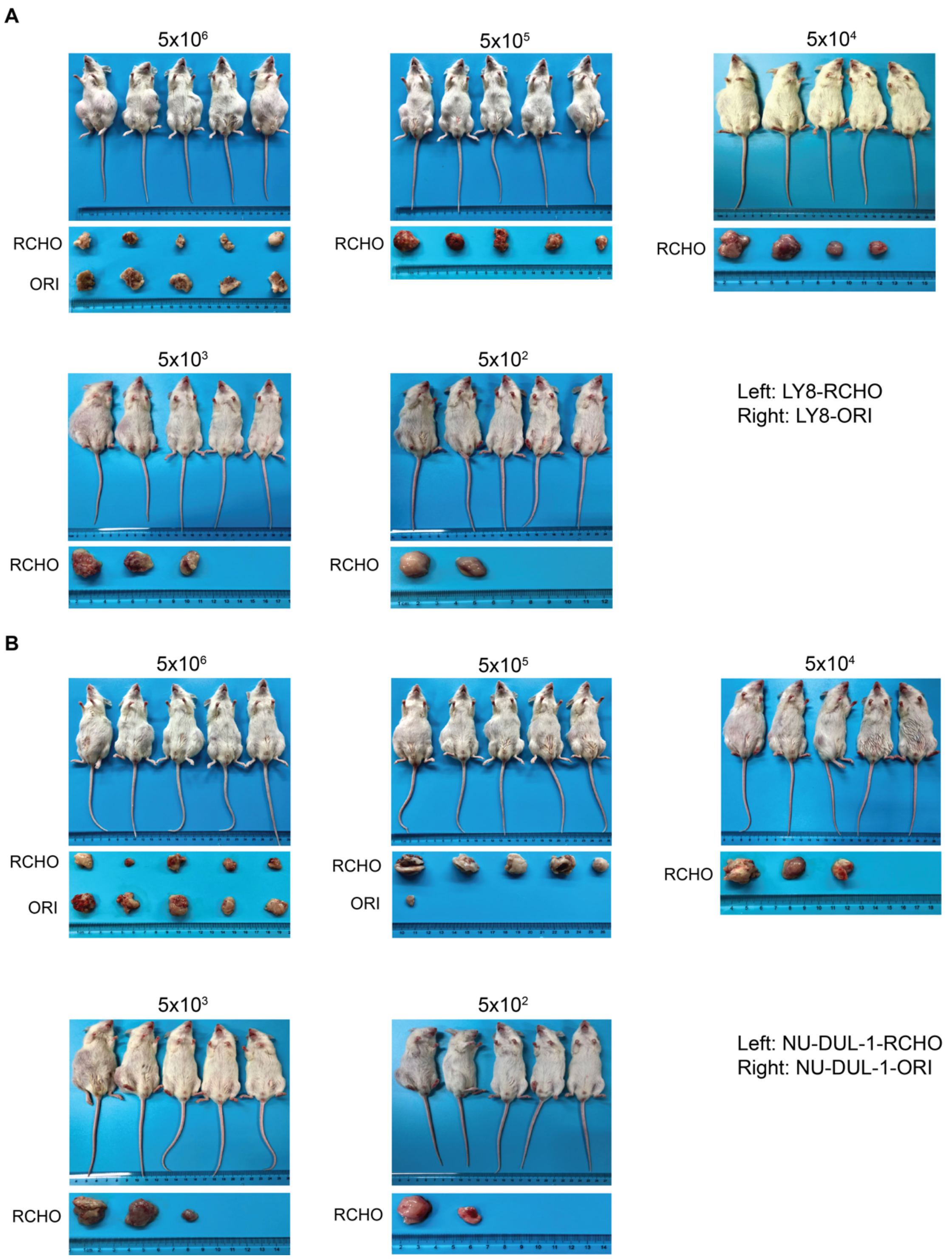

Figure S2. RCHO-resistant DLBCL cells displayed higher tumor-initiating capacity than original DLBCL cells. (**A-B**) The RCHO-resistant LY8 (A) and NU-DUL-1 (B) cells induced higher tumor incidence than the related original DLBCL cells. The quantitative results are concluded in Table 1 and Figures 5E and F.

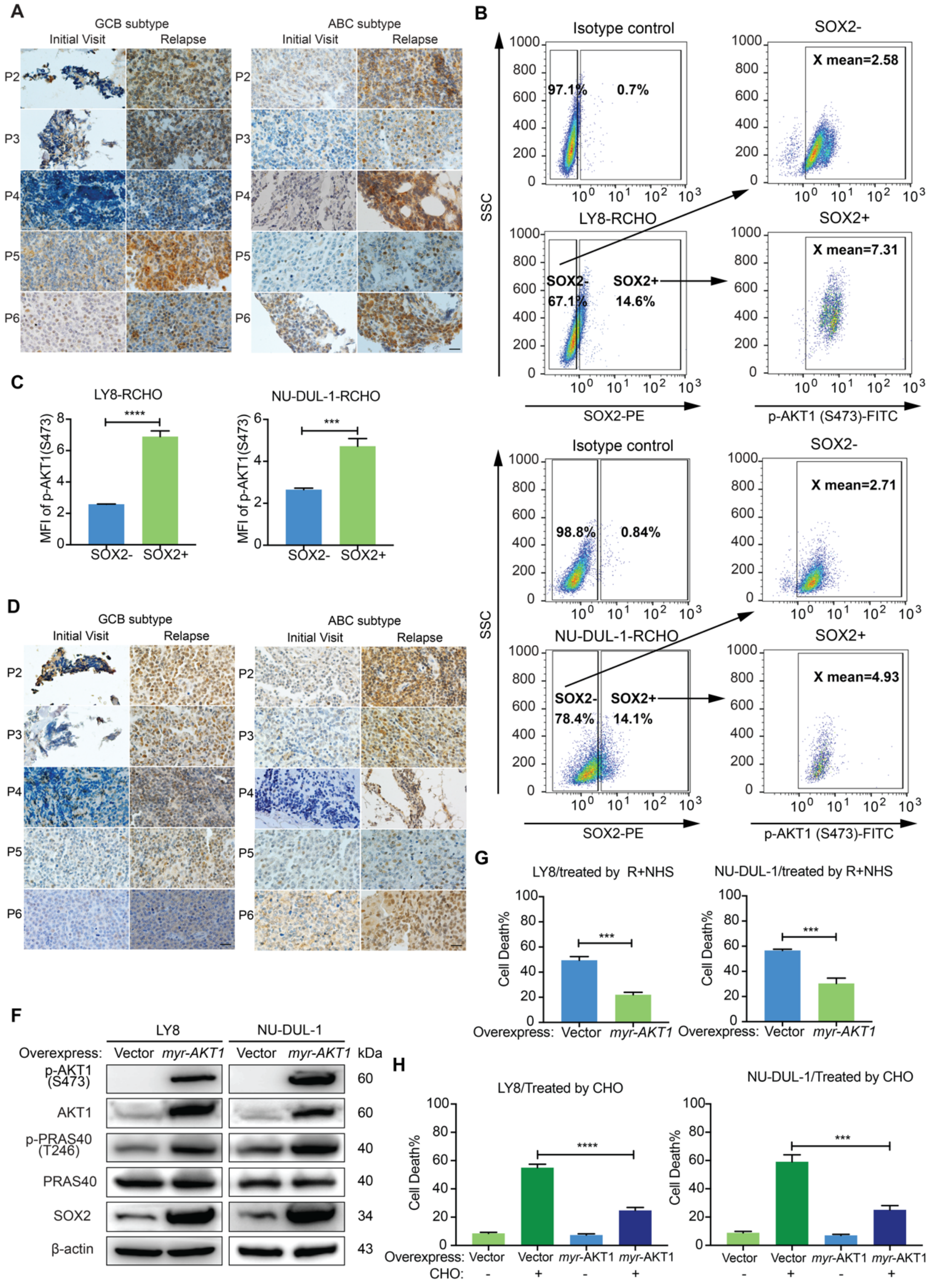

Figure S3. SOX2 levels is correlated with AKT1 activity. (**A**) SOX2 expression detected by IHC staining was markedly increased in relapsed 6 GCB (left panel) and 6 ABC (right panel) subtype DLBCL tissues compared with the paired patient tissues from the initial visit. Image from patient 1 and quantitative results for the GCB and ABC subtypes are shown in Figure 1I. P: patient. Scale bar: 20 µm. (**B-C**)

Representative images (B) for detecting p-AKT1 (S473) levels in $SOX2^-$ and $SOX2^+$ subpopulations of RCHO-resistant LY8 (up panel) and NU-DUL-1 (down panel) cells. The Fluorochrome-conjugated isotype-specific IgGs were used for gating the $SOX2^+$ cells and p-AKT1 (S473) positive cells. The quantitative results showed that $SOX2^+$ subpopulation exhibited higher p-AKT1 (S473) level than $SOX2^-$ subpopulation in RCHO-resistant LY8 (left panel) and NU-DUL-1 (right panel) cells. (**D**) AKT activation determined by IHC staining for p-AKT (S473) was significantly enhanced in relapsed 6 GCB (left panel) and 6 ABC (right panel) subtype DLBCL tissues compared with the paired patient tissues from the initial visit. Image from patient 1 and quantitative results for the GCB and ABC subtypes are shown in Figure 2E. P: patient. Scale bar: 20 μm. (**F**) Ectopic myr-AKT1 expression resulted in the constitutive AKT activation and elevated the levels of p-PRAS40 and SOX2. (**G**) CDC assays: ectopic myr-AKT1 expression reduced the susceptibility to R-mediated CDC. (**H**) CytoTox-Glo cytotoxicity assays: ectopic myr-AKT1 expression elevated the resistance to CHO treatment. The data are presented as mean ± SD; n=3. ***P<0.001, and ****P<0.0001.

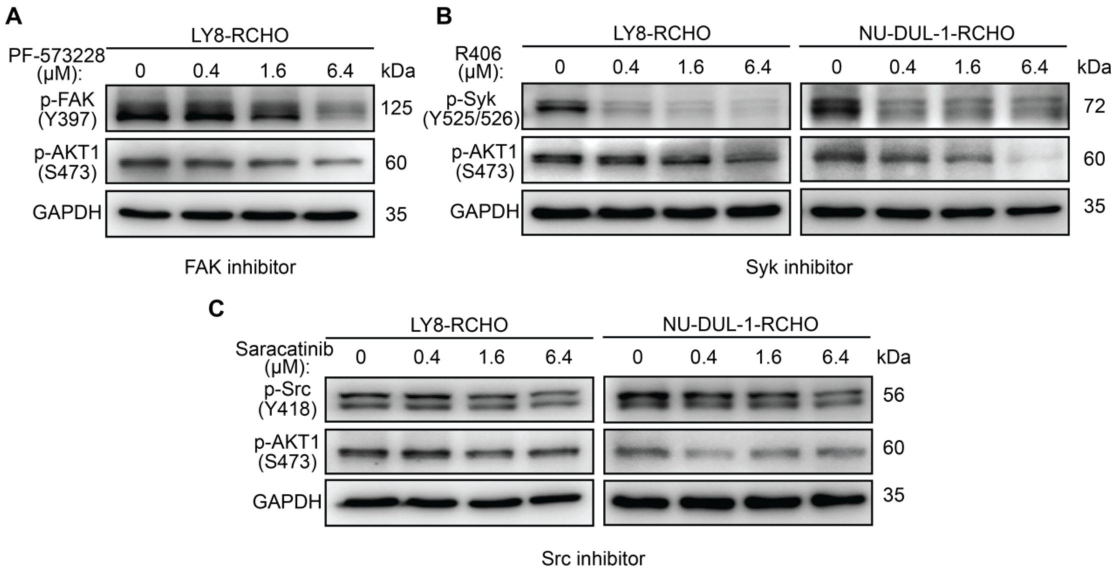

Figure S4. AKT activity after inhibition of upstream signals. (**A**) AKT activity is reduced after FAK inhibition by PF-573228 in LY8-RCHO cells. (**B**) Inhibition of Syk by R406 attenuated AKT activity in RCHO- resistant cells. (**C**) AKT activity is slightly reduced after Src inhibition by saracatinib in RCHO- resistant cells.

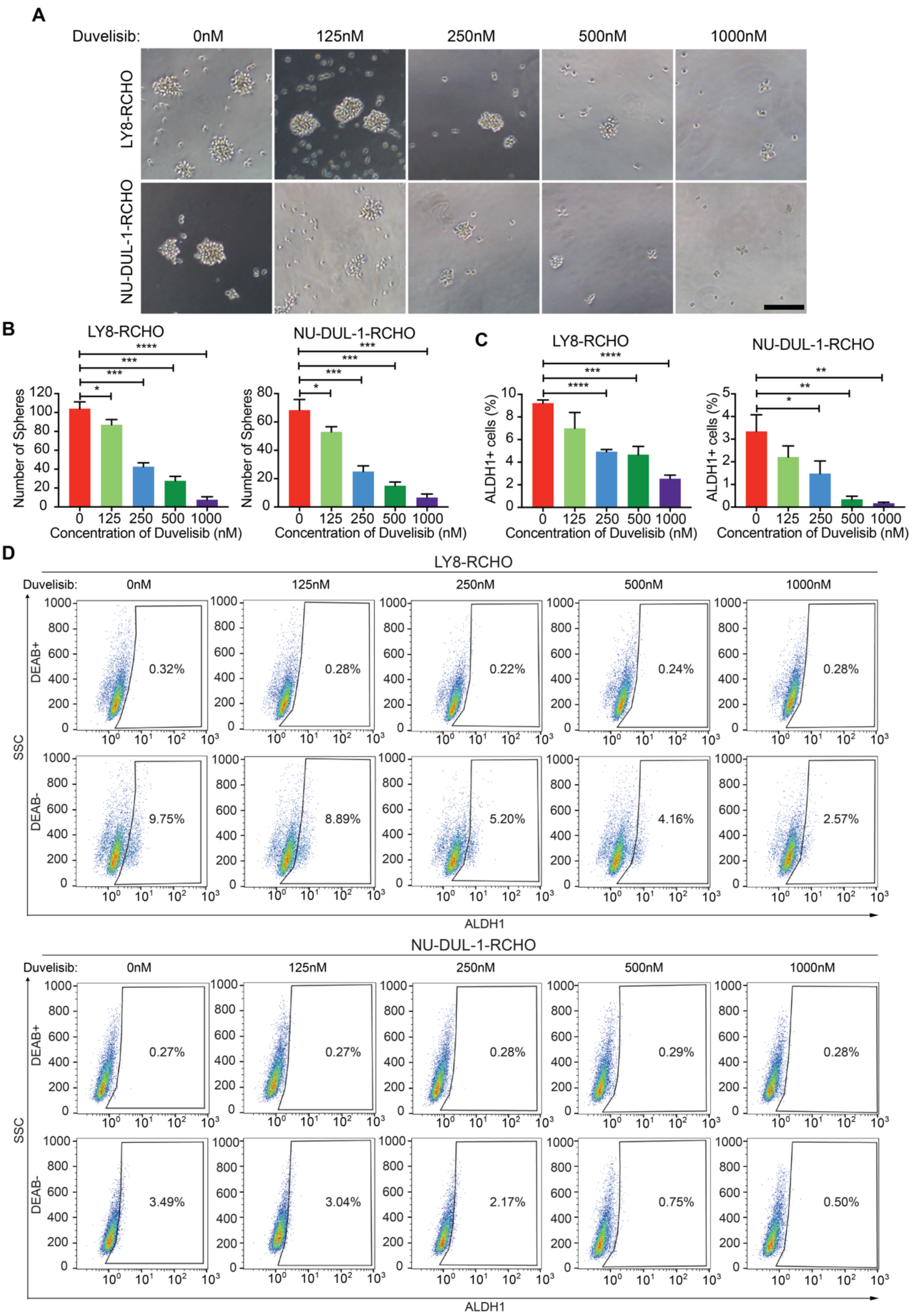

Figure S5. Duvelisib treatment reduced sphere-forming capacity of and ALDH1$^+$ subpopulation in RCHO-resistant cells. (**A-B**) Duvelisib treatment reduced the sphere-forming capacity of RCHO-resistant DLBCL cells. Representative images (A) and quantitative results (B). Scale bar: 100 μm. (**C-D**) Aldefluor assay: duvelisib

treatment reduced $ALDH1^+$ subpopulation. The RCHO-resistant DLBCL cells were treated by duvelisib for 24 h before the Aldefluor assay. The DEAB controls were used for gating the $ALDH1^+$ cells. Quantitative results (C) and representative images (D). The data are presented as mean ± SD; n=3. *P<0.05, **P<0.01, ***P<0.001 and ****P<0.0001.

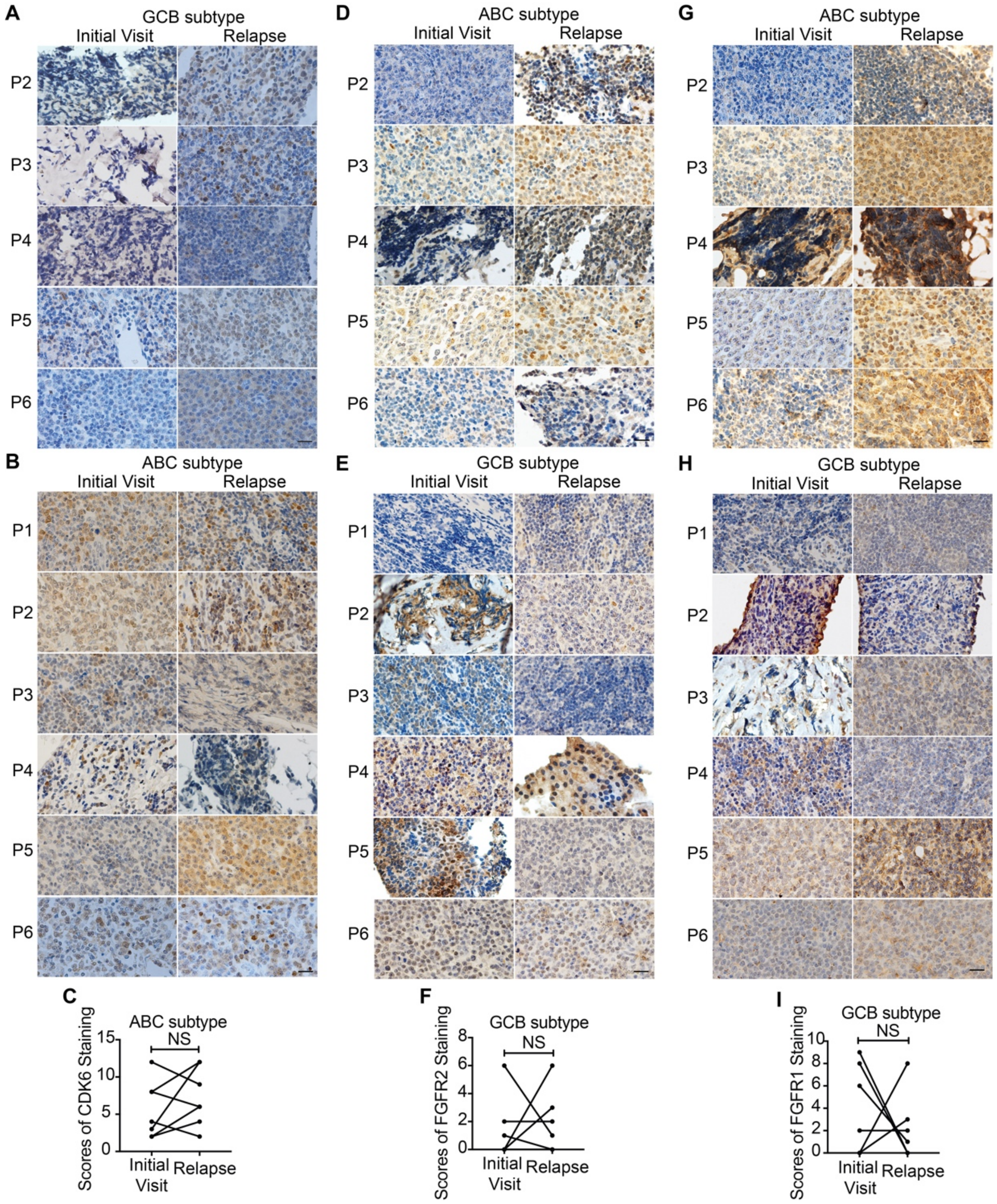

Figure S6. CDK6, FGFR1 and FGFR2 levels in clinical samples. (**A-C**) CDK6 expression detected by IHC staining significantly increased in relapsed 6 GCB (A) but not 6 ABC (B and C) subtype DLBCL tissues compared with the paired patient tissues

from the initial visit. The quantitative score for CDK6 staining of ABC subtype tissues is shown in C and was calculated from B. Image from patient 1 and quantitative results for the GCB subtype are shown in Figure 4C. P: patient. NS: no significance. Scale bar: 20 μm. (**D-F**) FGFR1 expression determined by IHC staining significantly increased in relapsed 6 ABC (D) but not 6 GCB (E and F) subtype DLBCL tissues compared with the paired patient tissues from the initial visit. The quantitative score for FGFR1 staining of GCB subtype tissues is shown in F and was calculated from E. Image from patient 1 and quantitative results for the ABC subtype are shown in Figure 4E. P: patient. NS: no significance. Scale bar: 20 μm. (**G-I**) FGFR2 expression detected by IHC staining was significantly increased in relapsed 6 ABC (G) but not 6 GCB (H and I) subtype DLBCL tissues compared with the paired patient tissues from the initial visit. The quantitative score for FGFR2 staining of GCB subtype tissues is shown in I and was calculated from H. Image from patient 1 and quantitative results for the ABC subtype are shown in Figure 4F. P: patient. NS: no significance. Scale bar: 20 μm.

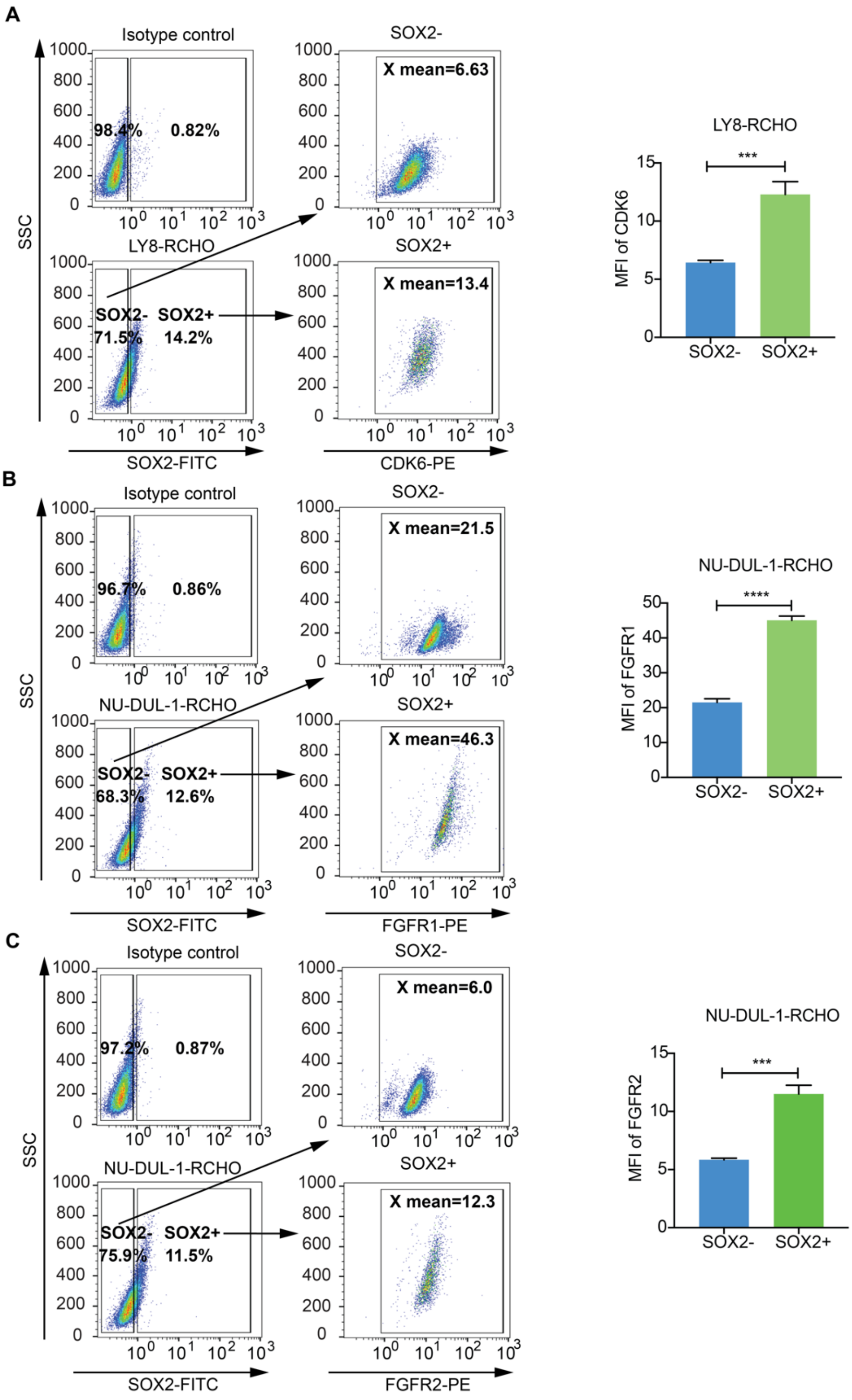

Figure S7. SOX2$^+$ subpupulation in RCHO-resistant cells showed higher CDK6 or FGFR1/2 level. (**A**) Representative images (left) for detecting CDK6 levels in SOX2$^-$ and SOX2$^+$ subpopulations of LY8-RCHO cells. The Fluorochrome-conjugated isotype-specific IgGs were used for gating the SOX2$^+$ cells and CDK6$^+$ cells. SOX2$^+$

subpopulation exhibits higher CDK6 level. The related quantitative results are shown in the right panel. (**B**-**C**) Representative images (left) for detecting FGFR1/2 levels in $SOX2^-$ and $SOX2^+$ subpopulations of NU-DUL-1-RCHO cells. The Fluorochrome-conjugated isotype-specific IgGs were used for gating the $SOX2^+$ cells and $FGFR1/2^+$ cells. $SOX2^+$ subpopulation exhibits higher FGFR1/2 level. The related quantitative results are shown in the right panel. The data are presented as mean ± SD; n=3. ***P<0.001, ****P<0.0001.

Supplementary Data 1 for Figure 2B

LY8-A

PathwayID	PathwayTerm	Symbol	P-Value	FDR	Enrichment
PATH:04144	Endocytosis	TGFBR1	0.010756	0.677813	2.49594575
PATH:04144	Endocytosis	SMAD6	0.010756	0.677813	2.49594575
PATH:04144	Endocytosis	RAB11FIP1	0.010756	0.677813	2.49594575
PATH:04144	Endocytosis	TFRC	0.010756	0.677813	2.49594575
PATH:04144	Endocytosis	MDM2	0.010756	0.677813	2.49594575
PATH:04144	Endocytosis	CHMP4C	0.010756	0.677813	2.49594575
PATH:04144	Endocytosis	HSPA1B	0.010756	0.677813	2.49594575
PATH:04144	Endocytosis	HSPA8	0.010756	0.677813	2.49594575
PATH:04144	Endocytosis	IGF1R	0.010756	0.677813	2.49594575
PATH:04144	Endocytosis	TGFB1	0.010756	0.677813	2.49594575
PATH:04115	p53 signaling pathway	SESN3	0.014425	0.677813	3.74391862
PATH:04115	p53 signaling pathway	ZMAT3	0.014425	0.677813	3.74391862
PATH:04115	p53 signaling pathway	MDM2	0.014425	0.677813	3.74391862
PATH:04115	p53 signaling pathway	RRM2B	0.014425	0.677813	3.74391862
PATH:04115	p53 signaling pathway	CDK6	0.014425	0.677813	3.74391862
PATH:04977	Vitamin digestion and absorption	APOB	0.015315	0.677813	6.36466165
PATH:04977	Vitamin digestion and absorption	SLC19A1	0.015315	0.677813	6.36466165
PATH:04977	Vitamin digestion and absorption	MMACHC	0.015315	0.677813	6.36466165
PATH:05220	Chronic myeloid leukemia	TGFBR1	0.018705	0.677813	3.48748584
PATH:05220	Chronic myeloid leukemia	MDM2	0.018705	0.677813	3.48748584
PATH:05220	Chronic myeloid leukemia	CDK6	0.018705	0.677813	3.48748584
PATH:05220	Chronic myeloid leukemia	RUNX1	0.018705	0.677813	3.48748584
PATH:05220	Chronic myeloid leukemia	TGFB1	0.018705	0.677813	3.48748584
PATH:04520	Adherens junction	WASF3	0.018705	0.677813	3.48748584
PATH:04520	Adherens junction	TGFBR1	0.018705	0.677813	3.48748584
PATH:04520	Adherens junction	LMO7	0.018705	0.677813	3.48748584
PATH:04520	Adherens junction	TJP1	0.018705	0.677813	3.48748584
PATH:04520	Adherens junction	IGF1R	0.018705	0.677813	3.48748584
PATH:04612	Antigen processing and presentation	CALR	0.021633	0.677813	3.34982192
PATH:04612	Antigen processing and presentation	TAPBP	0.021633	0.677813	3.34982192
PATH:04612	Antigen processing and presentation	HSPA5	0.021633	0.677813	3.34982192
PATH:04612	Antigen processing and presentation	HSPA1B	0.021633	0.677813	3.34982192
PATH:04612	Antigen processing and presentation	HSPA8	0.021633	0.677813	3.34982192
PATH:04151	PI3K-Akt signaling pathway	EFNA1	0.028662	0.677813	1.9075643
PATH:04151	PI3K-Akt signaling pathway	SGK1	0.028662	0.677813	1.9075643
PATH:04151	PI3K-Akt signaling pathway	MDM2	0.028662	0.677813	1.9075643
PATH:04151	PI3K-Akt signaling pathway	CDK6	0.028662	0.677813	1.9075643
PATH:04151	PI3K-Akt signaling pathway	JAK3	0.028662	0.677813	1.9075643
PATH:04151	PI3K-Akt signaling pathway	IL7	0.028662	0.677813	1.9075643
PATH:04151	PI3K-Akt signaling pathway	PDGFD	0.028662	0.677813	1.9075643
PATH:04151	PI3K-Akt signaling pathway	PGF	0.028662	0.677813	1.9075643
PATH:04151	PI3K-Akt signaling pathway	GNB4	0.028662	0.677813	1.9075643
PATH:04151	PI3K-Akt signaling pathway	LAMC3	0.028662	0.677813	1.9075643
PATH:04151	PI3K-Akt signaling pathway	PPP2R5E	0.028662	0.677813	1.9075643
PATH:04151	PI3K-Akt signaling pathway	IGF1R	0.028662	0.677813	1.9075643
PATH:04151	PI3K-Akt signaling pathway	EFNA5	0.028662	0.677813	1.9075643
PATH:04350	TGF-beta signaling pathway	TGFBR1	0.033462	0.677813	2.96030775
PATH:04350	TGF-beta signaling pathway	SMAD6	0.033462	0.677813	2.96030775
PATH:04350	TGF-beta signaling pathway	FST	0.033462	0.677813	2.96030775

PATH:04350 TGF-beta signaling pathway	LTBP1	0.033462	0.677813	2.96030775
PATH:04350 TGF-beta signaling pathway	TGFB1	0.033462	0.677813	2.96030775
PATH:04540 Gap junction	PRKCB	0.039121	0.677813	2.82873851
PATH:04540 Gap junction	TJP1	0.039121	0.677813	2.82873851
PATH:04540 Gap junction	GNA11	0.039121	0.677813	2.82873851
PATH:04540 Gap junction	TUBB2B	0.039121	0.677813	2.82873851
PATH:04540 Gap junction	PDGFD	0.039121	0.677813	2.82873851
PATH:05214 Glioma	PRKCB	0.046217	0.677813	3.13337189
PATH:05214 Glioma	MDM2	0.046217	0.677813	3.13337189
PATH:05214 Glioma	CDK6	0.046217	0.677813	3.13337189
PATH:05214 Glioma	IGF1R	0.046217	0.677813	3.13337189

LY8-B

PathwayID	PathwayTerm	Symbol	P-Value	FDR	Enrichment
PATH:04115	p53 signaling pathway	SESN3	0.00012442	0.02513219	5.33508403
PATH:04115	p53 signaling pathway	ZMAT3	0.00012442	0.02513219	5.33508403
PATH:04115	p53 signaling pathway	MDM2	0.00012442	0.02513219	5.33508403
PATH:04115	p53 signaling pathway	MDM4	0.00012442	0.02513219	5.33508403
PATH:04115	p53 signaling pathway	RRM2B	0.00012442	0.02513219	5.33508403
PATH:04115	p53 signaling pathway	BBC3	0.00012442	0.02513219	5.33508403
PATH:04115	p53 signaling pathway	CDK6	0.00012442	0.02513219	5.33508403
PATH:04115	p53 signaling pathway	CD82	0.00012442	0.02513219	5.33508403
PATH:04115	p53 signaling pathway	ATM	0.00012442	0.02513219	5.33508403
PATH:04390	Hippo signaling pathway	TGFBR1	0.00029676	0.02997256	3.42499222
PATH:04390	Hippo signaling pathway	SMAD1	0.00029676	0.02997256	3.42499222
PATH:04390	Hippo signaling pathway	DLG2	0.00029676	0.02997256	3.42499222
PATH:04390	Hippo signaling pathway	WNT10B	0.00029676	0.02997256	3.42499222
PATH:04390	Hippo signaling pathway	TCF7L2	0.00029676	0.02997256	3.42499222
PATH:04390	Hippo signaling pathway	MOB1B	0.00029676	0.02997256	3.42499222
PATH:04390	Hippo signaling pathway	BBC3	0.00029676	0.02997256	3.42499222
PATH:04390	Hippo signaling pathway	AJUBA	0.00029676	0.02997256	3.42499222
PATH:04390	Hippo signaling pathway	WNT3	0.00029676	0.02997256	3.42499222
PATH:04390	Hippo signaling pathway	RASSF6	0.00029676	0.02997256	3.42499222
PATH:04390	Hippo signaling pathway	BMP7	0.00029676	0.02997256	3.42499222
PATH:04390	Hippo signaling pathway	FZD1	0.00029676	0.02997256	3.42499222
PATH:04390	Hippo signaling pathway	TGFB1	0.00029676	0.02997256	3.42499222
PATH:05220	Chronic myeloid leukemia	TGFBR1	0.00089911	0.04730551	4.41748206
PATH:05220	Chronic myeloid leukemia	CBLC	0.00089911	0.04730551	4.41748206
PATH:05220	Chronic myeloid leukemia	MDM2	0.00089911	0.04730551	4.41748206
PATH:05220	Chronic myeloid leukemia	CTBP2	0.00089911	0.04730551	4.41748206
PATH:05220	Chronic myeloid leukemia	CDK6	0.00089911	0.04730551	4.41748206
PATH:05220	Chronic myeloid leukemia	RUNX1	0.00089911	0.04730551	4.41748206
PATH:05220	Chronic myeloid leukemia	CBL	0.00089911	0.04730551	4.41748206
PATH:05220	Chronic myeloid leukemia	TGFB1	0.00089911	0.04730551	4.41748206
PATH:04310	Wnt signaling pathway	WNT10B	0.00093674	0.04730551	3.16153128
PATH:04310	Wnt signaling pathway	NFAT5	0.00093674	0.04730551	3.16153128
PATH:04310	Wnt signaling pathway	TCF7L2	0.00093674	0.04730551	3.16153128
PATH:04310	Wnt signaling pathway	CTBP2	0.00093674	0.04730551	3.16153128
PATH:04310	Wnt signaling pathway	PRKCB	0.00093674	0.04730551	3.16153128
PATH:04310	Wnt signaling pathway	CHP1	0.00093674	0.04730551	3.16153128
PATH:04310	Wnt signaling pathway	WNT3	0.00093674	0.04730551	3.16153128
PATH:04310	Wnt signaling pathway	CREBBP	0.00093674	0.04730551	3.16153128
PATH:04310	Wnt signaling pathway	TBL1XR1	0.00093674	0.04730551	3.16153128
PATH:04310	Wnt signaling pathway	CAMK2B	0.00093674	0.04730551	3.16153128
PATH:04310	Wnt signaling pathway	FZD1	0.00093674	0.04730551	3.16153128
PATH:04310	Wnt signaling pathway	PPP2R5E	0.00093674	0.04730551	3.16153128
PATH:05200	Pathways in cancer	TGFBR1	0.00149388	0.06035285	2.32085137
PATH:05200	Pathways in cancer	CBLC	0.00149388	0.06035285	2.32085137
PATH:05200	Pathways in cancer	MDM2	0.00149388	0.06035285	2.32085137
PATH:05200	Pathways in cancer	WNT10B	0.00149388	0.06035285	2.32085137
PATH:05200	Pathways in cancer	TCF7L2	0.00149388	0.06035285	2.32085137
PATH:05200	Pathways in cancer	PGF	0.00149388	0.06035285	2.32085137
PATH:05200	Pathways in cancer	CTBP2	0.00149388	0.06035285	2.32085137

PATH:05200 Pathways in cancer	IGF1R	0.00149388	0.06035285	2.32085137
PATH:05200 Pathways in cancer	FOXO1	0.00149388	0.06035285	2.32085137
PATH:05200 Pathways in cancer	PRKCB	0.00149388	0.06035285	2.32085137
PATH:05200 Pathways in cancer	CCDC6	0.00149388	0.06035285	2.32085137
PATH:05200 Pathways in cancer	CDK6	0.00149388	0.06035285	2.32085137
PATH:05200 Pathways in cancer	WNT3	0.00149388	0.06035285	2.32085137
PATH:05200 Pathways in cancer	CREBBP	0.00149388	0.06035285	2.32085137
PATH:05200 Pathways in cancer	RUNX1	0.00149388	0.06035285	2.32085137
PATH:05200 Pathways in cancer	CBL	0.00149388	0.06035285	2.32085137
PATH:05200 Pathways in cancer	LAMC3	0.00149388	0.06035285	2.32085137
PATH:05200 Pathways in cancer	FZD1	0.00149388	0.06035285	2.32085137
PATH:05200 Pathways in cancer	TGFB1	0.00149388	0.06035285	2.32085137
PATH:04720 Long-term potentiation	GRIN2C	0.00294526	0.09915712	4.03095238
PATH:04720 Long-term potentiation	CALML6	0.00294526	0.09915712	4.03095238
PATH:04720 Long-term potentiation	PRKCB	0.00294526	0.09915712	4.03095238
PATH:04720 Long-term potentiation	CHP1	0.00294526	0.09915712	4.03095238
PATH:04720 Long-term potentiation	CREBBP	0.00294526	0.09915712	4.03095238
PATH:04720 Long-term potentiation	CAMK2B	0.00294526	0.09915712	4.03095238
PATH:04720 Long-term potentiation	ITPR2	0.00294526	0.09915712	4.03095238
PATH:04020 Calcium signaling pathway	ITPKB	0.00386727	0.1115985	2.62888199
PATH:04020 Calcium signaling pathway	GNA11	0.00386727	0.1115985	2.62888199
PATH:04020 Calcium signaling pathway	GRIN2C	0.00386727	0.1115985	2.62888199
PATH:04020 Calcium signaling pathway	TRPC1	0.00386727	0.1115985	2.62888199
PATH:04020 Calcium signaling pathway	CALML6	0.00386727	0.1115985	2.62888199
PATH:04020 Calcium signaling pathway	PRKCB	0.00386727	0.1115985	2.62888199
PATH:04020 Calcium signaling pathway	MYLK	0.00386727	0.1115985	2.62888199
PATH:04020 Calcium signaling pathway	CHP1	0.00386727	0.1115985	2.62888199
PATH:04020 Calcium signaling pathway	PLCD3	0.00386727	0.1115985	2.62888199
PATH:04020 Calcium signaling pathway	HRH1	0.00386727	0.1115985	2.62888199
PATH:04020 Calcium signaling pathway	CAMK2B	0.00386727	0.1115985	2.62888199
PATH:04020 Calcium signaling pathway	ITPR2	0.00386727	0.1115985	2.62888199
PATH:04916 Melanogenesis	WNT10B	0.00573845	0.12785961	3.19283357
PATH:04916 Melanogenesis	TCF7L2	0.00573845	0.12785961	3.19283357
PATH:04916 Melanogenesis	CALML6	0.00573845	0.12785961	3.19283357
PATH:04916 Melanogenesis	PRKCB	0.00573845	0.12785961	3.19283357
PATH:04916 Melanogenesis	WNT3	0.00573845	0.12785961	3.19283357
PATH:04916 Melanogenesis	CREBBP	0.00573845	0.12785961	3.19283357
PATH:04916 Melanogenesis	CAMK2B	0.00573845	0.12785961	3.19283357
PATH:04916 Melanogenesis	FZD1	0.00573845	0.12785961	3.19283357
PATH:04070 Phosphatidylinositol signaling system	ITPKB	0.00613686	0.12785961	3.48353909
PATH:04070 Phosphatidylinositol signaling system	DGKH	0.00613686	0.12785961	3.48353909
PATH:04070 Phosphatidylinositol signaling system	INPP4B	0.00613686	0.12785961	3.48353909
PATH:04070 Phosphatidylinositol signaling system	CALML6	0.00613686	0.12785961	3.48353909
PATH:04070 Phosphatidylinositol signaling system	PRKCB	0.00613686	0.12785961	3.48353909
PATH:04070 Phosphatidylinositol signaling system	PLCD3	0.00613686	0.12785961	3.48353909
PATH:04070 Phosphatidylinositol signaling system	ITPR2	0.00613686	0.12785961	3.48353909
PATH:05205 Proteoglycans in cancer	CBLC	0.00698616	0.12785961	2.32899471
PATH:05205 Proteoglycans in cancer	ANK3	0.00698616	0.12785961	2.32899471
PATH:05205 Proteoglycans in cancer	MDM2	0.00698616	0.12785961	2.32899471
PATH:05205 Proteoglycans in cancer	WNT10B	0.00698616	0.12785961	2.32899471

PATH:05205 Proteoglycans in cancer	ESR1	0.00698616	0.12785961	2.32899471
PATH:05205 Proteoglycans in cancer	IGF1R	0.00698616	0.12785961	2.32899471
PATH:05205 Proteoglycans in cancer	PRKCB	0.00698616	0.12785961	2.32899471
PATH:05205 Proteoglycans in cancer	WNT3	0.00698616	0.12785961	2.32899471
PATH:05205 Proteoglycans in cancer	CBL	0.00698616	0.12785961	2.32899471
PATH:05205 Proteoglycans in cancer	CAMK2B	0.00698616	0.12785961	2.32899471
PATH:05205 Proteoglycans in cancer	FZD1	0.00698616	0.12785961	2.32899471
PATH:05205 Proteoglycans in cancer	TGFB1	0.00698616	0.12785961	2.32899471
PATH:05205 Proteoglycans in cancer	ITPR2	0.00698616	0.12785961	2.32899471
PATH:05214 Glioma	MDM2	0.00809319	0.12785961	3.72087912
PATH:05214 Glioma	IGF1R	0.00809319	0.12785961	3.72087912
PATH:05214 Glioma	CALML6	0.00809319	0.12785961	3.72087912
PATH:05214 Glioma	PRKCB	0.00809319	0.12785961	3.72087912
PATH:05214 Glioma	CDK6	0.00809319	0.12785961	3.72087912
PATH:05214 Glioma	CAMK2B	0.00809319	0.12785961	3.72087912
PATH:04144 Endocytosis	TGFBR1	0.00812174	0.12785961	2.37114846
PATH:04144 Endocytosis	CBLC	0.00812174	0.12785961	2.37114846
PATH:04144 Endocytosis	MDM2	0.00812174	0.12785961	2.37114846
PATH:04144 Endocytosis	CXCR2	0.00812174	0.12785961	2.37114846
PATH:04144 Endocytosis	IGF1R	0.00812174	0.12785961	2.37114846
PATH:04144 Endocytosis	MVB12B	0.00812174	0.12785961	2.37114846
PATH:04144 Endocytosis	SMAD6	0.00812174	0.12785961	2.37114846
PATH:04144 Endocytosis	DNAJC6	0.00812174	0.12785961	2.37114846
PATH:04144 Endocytosis	TFRC	0.00812174	0.12785961	2.37114846
PATH:04144 Endocytosis	HSPA1B	0.00812174	0.12785961	2.37114846
PATH:04144 Endocytosis	CBL	0.00812174	0.12785961	2.37114846
PATH:04144 Endocytosis	TGFB1	0.00812174	0.12785961	2.37114846
PATH:04350 TGF-beta signaling pathway	TGFBR1	0.00822859	0.12785961	3.28100775
PATH:04350 TGF-beta signaling pathway	FST	0.00822859	0.12785961	3.28100775
PATH:04350 TGF-beta signaling pathway	SMAD1	0.00822859	0.12785961	3.28100775
PATH:04350 TGF-beta signaling pathway	SMAD6	0.00822859	0.12785961	3.28100775
PATH:04350 TGF-beta signaling pathway	CREBBP	0.00822859	0.12785961	3.28100775
PATH:04350 TGF-beta signaling pathway	BMP7	0.00822859	0.12785961	3.28100775
PATH:04350 TGF-beta signaling pathway	TGFB1	0.00822859	0.12785961	3.28100775
PATH:04068 FoxO signaling pathway	TGFBR1	0.00900157	0.12987974	2.72771214
PATH:04068 FoxO signaling pathway	MDM2	0.00900157	0.12987974	2.72771214
PATH:04068 FoxO signaling pathway	IGF1R	0.00900157	0.12987974	2.72771214
PATH:04068 FoxO signaling pathway	FOXO1	0.00900157	0.12987974	2.72771214
PATH:04068 FoxO signaling pathway	BCL6	0.00900157	0.12987974	2.72771214
PATH:04068 FoxO signaling pathway	CREBBP	0.00900157	0.12987974	2.72771214
PATH:04068 FoxO signaling pathway	ATM	0.00900157	0.12987974	2.72771214
PATH:04068 FoxO signaling pathway	TGFB1	0.00900157	0.12987974	2.72771214
PATH:04068 FoxO signaling pathway	FOXO3	0.00900157	0.12987974	2.72771214
PATH:04520 Adherens junction	WASF3	0.01329318	0.17901486	3.31311155
PATH:04520 Adherens junction	TGFBR1	0.01329318	0.17901486	3.31311155
PATH:04520 Adherens junction	TJP1	0.01329318	0.17901486	3.31311155
PATH:04520 Adherens junction	TCF7L2	0.01329318	0.17901486	3.31311155
PATH:04520 Adherens junction	IGF1R	0.01329318	0.17901486	3.31311155
PATH:04520 Adherens junction	CREBBP	0.01329318	0.17901486	3.31311155
PATH:00531 Glycosaminoglycan degradation	HGSNAT	0.01590873	0.20084776	6.36466165

PATH:00531 Glycosaminoglycan degradation	IDUA	0.01590873	0.20084776	6.36466165
PATH:00531 Glycosaminoglycan degradation	HYAL3	0.01590873	0.20084776	6.36466165
PATH:00100 Steroid biosynthesis	CYP27B1	0.01796432	0.21345839	6.04642857
PATH:00100 Steroid biosynthesis	TM7SF2	0.01796432	0.21345839	6.04642857
PATH:00100 Steroid biosynthesis	FAXDC2	0.01796432	0.21345839	6.04642857
PATH:05166 HTLV-I infection	TGFBR1	0.02167204	0.22721248	1.98493867
PATH:05166 HTLV-I infection	CRTC1	0.02167204	0.22721248	1.98493867
PATH:05166 HTLV-I infection	WNT10B	0.02167204	0.22721248	1.98493867
PATH:05166 HTLV-I infection	NFAT5	0.02167204	0.22721248	1.98493867
PATH:05166 HTLV-I infection	ICAM1	0.02167204	0.22721248	1.98493867
PATH:05166 HTLV-I infection	JAK3	0.02167204	0.22721248	1.98493867
PATH:05166 HTLV-I infection	CHP1	0.02167204	0.22721248	1.98493867
PATH:05166 HTLV-I infection	WNT3	0.02167204	0.22721248	1.98493867
PATH:05166 HTLV-I infection	TP53INP1	0.02167204	0.22721248	1.98493867
PATH:05166 HTLV-I infection	CREBBP	0.02167204	0.22721248	1.98493867
PATH:05166 HTLV-I infection	ATM	0.02167204	0.22721248	1.98493867
PATH:05166 HTLV-I infection	FZD1	0.02167204	0.22721248	1.98493867
PATH:05166 HTLV-I infection	TGFB1	0.02167204	0.22721248	1.98493867
PATH:04360 Axon guidance	EFNA1	0.02221893	0.22721248	2.46165031
PATH:04360 Axon guidance	SEMA3F	0.02221893	0.22721248	2.46165031
PATH:04360 Axon guidance	NFAT5	0.02221893	0.22721248	2.46165031
PATH:04360 Axon guidance	ABLIM2	0.02221893	0.22721248	2.46165031
PATH:04360 Axon guidance	CHP1	0.02221893	0.22721248	2.46165031
PATH:04360 Axon guidance	PLXNB1	0.02221893	0.22721248	2.46165031
PATH:04360 Axon guidance	SEMA4B	0.02221893	0.22721248	2.46165031
PATH:04360 Axon guidance	EFNA5	0.02221893	0.22721248	2.46165031
PATH:00532 Glycosaminoglycan biosynthesis - cho	CHST11	0.02249629	0.22721248	5.49675325
PATH:00532 Glycosaminoglycan biosynthesis - cho	B3GALT6	0.02249629	0.22721248	5.49675325
PATH:00532 Glycosaminoglycan biosynthesis - cho	CHSY1	0.02249629	0.22721248	5.49675325
PATH:04919 Thyroid hormone signaling pathway	MDM2	0.03650286	0.34141951	2.37114846
PATH:04919 Thyroid hormone signaling pathway	ESR1	0.03650286	0.34141951	2.37114846
PATH:04919 Thyroid hormone signaling pathway	FOXO1	0.03650286	0.34141951	2.37114846
PATH:04919 Thyroid hormone signaling pathway	PRKCB	0.03650286	0.34141951	2.37114846
PATH:04919 Thyroid hormone signaling pathway	PLCD3	0.03650286	0.34141951	2.37114846
PATH:04919 Thyroid hormone signaling pathway	CREBBP	0.03650286	0.34141951	2.37114846
PATH:04919 Thyroid hormone signaling pathway	MED13	0.03650286	0.34141951	2.37114846
PATH:05031 Amphetamine addiction	GRIN2C	0.0371843	0.34141951	2.8792517
PATH:05031 Amphetamine addiction	CALML6	0.0371843	0.34141951	2.8792517
PATH:05031 Amphetamine addiction	PRKCB	0.0371843	0.34141951	2.8792517
PATH:05031 Amphetamine addiction	CHP1	0.0371843	0.34141951	2.8792517
PATH:05031 Amphetamine addiction	CAMK2B	0.0371843	0.34141951	2.8792517
PATH:04270 Vascular smooth muscle contraction	GNA11	0.03919478	0.34268197	2.33195592
PATH:04270 Vascular smooth muscle contraction	CALML6	0.03919478	0.34268197	2.33195592
PATH:04270 Vascular smooth muscle contraction	PRKCB	0.03919478	0.34268197	2.33195592
PATH:04270 Vascular smooth muscle contraction	MYLK	0.03919478	0.34268197	2.33195592
PATH:04270 Vascular smooth muscle contraction	ACTA2	0.03919478	0.34268197	2.33195592
PATH:04270 Vascular smooth muscle contraction	PLA2G4C	0.03919478	0.34268197	2.33195592
PATH:04270 Vascular smooth muscle contraction	ITPR2	0.03919478	0.34268197	2.33195592
PATH:04730 Long-term depression	GNA11	0.04285085	0.34268197	2.76092629
PATH:04730 Long-term depression	IGF1R	0.04285085	0.34268197	2.76092629

PATH:04730 Long-term depression	PRKCB	0.04285085	0.34268197	2.76092629
PATH:04730 Long-term depression	PLA2G4C	0.04285085	0.34268197	2.76092629
PATH:04730 Long-term depression	ITPR2	0.04285085	0.34268197	2.76092629
PATH:04750 Inflammatory mediator regulation of T	CALML6	0.0433598	0.34268197	2.46793003
PATH:04750 Inflammatory mediator regulation of T	PRKCB	0.0433598	0.34268197	2.46793003
PATH:04750 Inflammatory mediator regulation of T	HRH1	0.0433598	0.34268197	2.46793003
PATH:04750 Inflammatory mediator regulation of T	PLA2G4C	0.0433598	0.34268197	2.46793003
PATH:04750 Inflammatory mediator regulation of T	CAMK2B	0.0433598	0.34268197	2.46793003
PATH:04750 Inflammatory mediator regulation of T	ITPR2	0.0433598	0.34268197	2.46793003
PATH:05152 Tuberculosis	CYP27B1	0.04410758	0.34268197	2.02673583
PATH:05152 Tuberculosis	IRAK2	0.04410758	0.34268197	2.02673583
PATH:05152 Tuberculosis	TLR9	0.04410758	0.34268197	2.02673583
PATH:05152 Tuberculosis	CALML6	0.04410758	0.34268197	2.02673583
PATH:05152 Tuberculosis	VDR	0.04410758	0.34268197	2.02673583
PATH:05152 Tuberculosis	CHP1	0.04410758	0.34268197	2.02673583
PATH:05152 Tuberculosis	CREBBP	0.04410758	0.34268197	2.02673583
PATH:05152 Tuberculosis	CAMK2B	0.04410758	0.34268197	2.02673583
PATH:05152 Tuberculosis	TGFB1	0.04410758	0.34268197	2.02673583
PATH:04971 Gastric acid secretion	CALML6	0.0468995	0.35087777	2.68730159
PATH:04971 Gastric acid secretion	PRKCB	0.0468995	0.35087777	2.68730159
PATH:04971 Gastric acid secretion	MYLK	0.0468995	0.35087777	2.68730159
PATH:04971 Gastric acid secretion	CAMK2B	0.0468995	0.35087777	2.68730159
PATH:04971 Gastric acid secretion	ITPR2	0.0468995	0.35087777	2.68730159

NU-DUL-1-A

PathwayID	PathwayTerm	Symbol	P-Value	FDR	Enrichment
PATH:00051	Fructose and mannose metabolism	GMPPA	0.00419283	0.42474531	9.11424731
PATH:00051	Fructose and mannose metabolism	KHK	0.00419283	0.42474531	9.11424731
PATH:00051	Fructose and mannose metabolism	AKR1B10	0.00419283	0.42474531	9.11424731
PATH:00565	Ether lipid metabolism	PAFAH1B3	0.00648466	0.42474531	7.81221198
PATH:00565	Ether lipid metabolism	PPAP2C	0.00648466	0.42474531	7.81221198
PATH:00565	Ether lipid metabolism	PLA2G16	0.00648466	0.42474531	7.81221198
PATH:00100	Steroid biosynthesis	FDFT1	0.01405854	0.61388956	10.9370968
PATH:00100	Steroid biosynthesis	CYP27B1	0.01405854	0.61388956	10.9370968
PATH:03018	RNA degradation	CNOT7	0.03300132	0.61600553	4.26120654
PATH:03018	RNA degradation	EXOSC4	0.03300132	0.61600553	4.26120654
PATH:03018	RNA degradation	LSM1	0.03300132	0.61600553	4.26120654
PATH:05160	Hepatitis C	TICAM1	0.03610805	0.61600553	3.19331293
PATH:05160	Hepatitis C	PPP2R2A	0.03610805	0.61600553	3.19331293
PATH:05160	Hepatitis C	PPP2CB	0.03610805	0.61600553	3.19331293
PATH:05160	Hepatitis C	CDKN1A	0.03610805	0.61600553	3.19331293
PATH:04650	Natural killer cell mediated cytotoxicity	HCST	0.03693824	0.61600553	3.17017298
PATH:04650	Natural killer cell mediated cytotoxicity	ICAM2	0.03693824	0.61600553	3.17017298
PATH:04650	Natural killer cell mediated cytotoxicity	TNFRSF10A	0.03693824	0.61600553	3.17017298
PATH:04650	Natural killer cell mediated cytotoxicity	PPP3CC	0.03693824	0.61600553	3.17017298
PATH:04151	PI3K-Akt signaling pathway	PPP2R2A	0.0378893	0.61600553	2.20633076
PATH:04151	PI3K-Akt signaling pathway	EIF4EBP1	0.0378893	0.61600553	2.20633076
PATH:04151	PI3K-Akt signaling pathway	FGFR1	0.0378893	0.61600553	2.20633076
PATH:04151	PI3K-Akt signaling pathway	PPP2CB	0.0378893	0.61600553	2.20633076
PATH:04151	PI3K-Akt signaling pathway	IL2RA	0.0378893	0.61600553	2.20633076
PATH:04151	PI3K-Akt signaling pathway	NR4A1	0.0378893	0.61600553	2.20633076
PATH:04151	PI3K-Akt signaling pathway	CDKN1A	0.0378893	0.61600553	2.20633076
PATH:00591	Linoleic acid metabolism	PLA2G16	0.03825944	0.61600553	6.43358634
PATH:00591	Linoleic acid metabolism	AKR1B10	0.03825944	0.61600553	6.43358634

NU-DUL-1-B

PathwayID	PathwayTerm	Symbol	P-Value	FDR	Enrichment
PATH:00100	Steroid biosynthesis	FDFT1	0.00055243	0.109382	10.1208955
PATH:00100	Steroid biosynthesis	MSMO1	0.00055243	0.109382	10.1208955
PATH:00100	Steroid biosynthesis	TM7SF2	0.00055243	0.109382	10.1208955
PATH:00100	Steroid biosynthesis	CYP27B1	0.00055243	0.109382	10.1208955
PATH:05323	Rheumatoid arthritis	FLT1	0.00204944	0.20289462	3.89265212
PATH:05323	Rheumatoid arthritis	VEGFB	0.00204944	0.20289462	3.89265212
PATH:05323	Rheumatoid arthritis	ACP5	0.00204944	0.20289462	3.89265212
PATH:05323	Rheumatoid arthritis	PGF	0.00204944	0.20289462	3.89265212
PATH:05323	Rheumatoid arthritis	ATP6V1B2	0.00204944	0.20289462	3.89265212
PATH:05323	Rheumatoid arthritis	HLA-DQB1	0.00204944	0.20289462	3.89265212
PATH:05323	Rheumatoid arthritis	TNFSF13	0.00204944	0.20289462	3.89265212
PATH:04151	PI3K-Akt signaling pathway	PPP2R2A	0.0033645	0.22205703	2.18751344
PATH:04151	PI3K-Akt signaling pathway	FLT1	0.0033645	0.22205703	2.18751344
PATH:04151	PI3K-Akt signaling pathway	VEGFB	0.0033645	0.22205703	2.18751344
PATH:04151	PI3K-Akt signaling pathway	EIF4EBP1	0.0033645	0.22205703	2.18751344
PATH:04151	PI3K-Akt signaling pathway	PPP2CB	0.0033645	0.22205703	2.18751344
PATH:04151	PI3K-Akt signaling pathway	COL4A3	0.0033645	0.22205703	2.18751344
PATH:04151	PI3K-Akt signaling pathway	RXRA	0.0033645	0.22205703	2.18751344
PATH:04151	PI3K-Akt signaling pathway	PGF	0.0033645	0.22205703	2.18751344
PATH:04151	PI3K-Akt signaling pathway	CDKN1A	0.0033645	0.22205703	2.18751344
PATH:04151	PI3K-Akt signaling pathway	EFNA3	0.0033645	0.22205703	2.18751344
PATH:04151	PI3K-Akt signaling pathway	PCK2	0.0033645	0.22205703	2.18751344
PATH:04151	PI3K-Akt signaling pathway	FN1	0.0033645	0.22205703	2.18751344
PATH:04151	PI3K-Akt signaling pathway	IL2RA	0.0033645	0.22205703	2.18751344
PATH:04151	PI3K-Akt signaling pathway	CREB3L1	0.0033645	0.22205703	2.18751344
PATH:04151	PI3K-Akt signaling pathway	DDIT4	0.0033645	0.22205703	2.18751344
PATH:01230	Biosynthesis of amino acids	ASL	0.0143649	0.55309566	3.46606011
PATH:01230	Biosynthesis of amino acids	ACO1	0.0143649	0.55309566	3.46606011
PATH:01230	Biosynthesis of amino acids	ALDOC	0.0143649	0.55309566	3.46606011
PATH:01230	Biosynthesis of amino acids	PYCR1	0.0143649	0.55309566	3.46606011
PATH:01230	Biosynthesis of amino acids	SDSL	0.0143649	0.55309566	3.46606011
PATH:05166	HTLV-I infection	WNT3	0.0150468	0.55309566	2.1085199
PATH:05166	HTLV-I infection	HLA-F	0.0150468	0.55309566	2.1085199
PATH:05166	HTLV-I infection	PPP3CC	0.0150468	0.55309566	2.1085199
PATH:05166	HTLV-I infection	RRAS	0.0150468	0.55309566	2.1085199
PATH:05166	HTLV-I infection	CDKN1A	0.0150468	0.55309566	2.1085199
PATH:05166	HTLV-I infection	HLA-DQB1	0.0150468	0.55309566	2.1085199
PATH:05166	HTLV-I infection	ANAPC11	0.0150468	0.55309566	2.1085199
PATH:05166	HTLV-I infection	WNT10A	0.0150468	0.55309566	2.1085199
PATH:05166	HTLV-I infection	IL2RA	0.0150468	0.55309566	2.1085199
PATH:05166	HTLV-I infection	NFKB2	0.0150468	0.55309566	2.1085199
PATH:05166	HTLV-I infection	FZD3	0.0150468	0.55309566	2.1085199
PATH:00740	Riboflavin metabolism	ACP5	0.02247132	0.55309566	8.4340796
PATH:00740	Riboflavin metabolism	BLVRB	0.02247132	0.55309566	8.4340796
PATH:05134	Legionellosis	PYCARD	0.02286737	0.55309566	3.68032564
PATH:05134	Legionellosis	HSPA1A	0.02286737	0.55309566	3.68032564
PATH:05134	Legionellosis	EEF1A2	0.02286737	0.55309566	3.68032564
PATH:05134	Legionellosis	NFKB2	0.02286737	0.55309566	3.68032564
PATH:05130	Pathogenic Escherichia coli infection	TUBB2A	0.02425991	0.55309566	3.61460554

PATH:05130 Pathogenic Escherichia coli infection	TUBB2B	0.02425991	0.55309566	3.61460554
PATH:05130 Pathogenic Escherichia coli infection	TUBB3	0.02425991	0.55309566	3.61460554
PATH:05130 Pathogenic Escherichia coli infection	KRT18	0.02425991	0.55309566	3.61460554
PATH:00330 Arginine and proline metabolism	ASL	0.02874111	0.55309566	3.43081204
PATH:00330 Arginine and proline metabolism	OAT	0.02874111	0.55309566	3.43081204
PATH:00330 Arginine and proline metabolism	PYCR1	0.02874111	0.55309566	3.43081204
PATH:00330 Arginine and proline metabolism	SAT2	0.02874111	0.55309566	3.43081204
PATH:04390 Hippo signaling pathway	PPP2R2A	0.03148146	0.55309566	2.31523754
PATH:04390 Hippo signaling pathway	WNT3	0.03148146	0.55309566	2.31523754
PATH:04390 Hippo signaling pathway	PPP2CB	0.03148146	0.55309566	2.31523754
PATH:04390 Hippo signaling pathway	SERPINE1	0.03148146	0.55309566	2.31523754
PATH:04390 Hippo signaling pathway	LLGL2	0.03148146	0.55309566	2.31523754
PATH:04390 Hippo signaling pathway	WNT10A	0.03148146	0.55309566	2.31523754
PATH:04390 Hippo signaling pathway	FZD3	0.03148146	0.55309566	2.31523754
PATH:04145 Phagosome	TUBB2A	0.03245633	0.55309566	2.30020353
PATH:04145 Phagosome	RILP	0.03245633	0.55309566	2.30020353
PATH:04145 Phagosome	HLA-F	0.03245633	0.55309566	2.30020353
PATH:04145 Phagosome	TUBB2B	0.03245633	0.55309566	2.30020353
PATH:04145 Phagosome	ATP6V1B2	0.03245633	0.55309566	2.30020353
PATH:04145 Phagosome	HLA-DQB1	0.03245633	0.55309566	2.30020353
PATH:04145 Phagosome	TUBB3	0.03245633	0.55309566	2.30020353
PATH:00051 Fructose and mannose metabolism	GMPPA	0.03352095	0.55309566	4.2170398
PATH:00051 Fructose and mannose metabolism	KHK	0.03352095	0.55309566	4.2170398
PATH:00051 Fructose and mannose metabolism	ALDOC	0.03352095	0.55309566	4.2170398
PATH:04150 mTOR signaling pathway	VEGFB	0.0372375	0.56715579	3.16277985
PATH:04150 mTOR signaling pathway	EIF4EBP1	0.0372375	0.56715579	3.16277985
PATH:04150 mTOR signaling pathway	PGF	0.0372375	0.56715579	3.16277985
PATH:04150 mTOR signaling pathway	DDIT4	0.0372375	0.56715579	3.16277985
PATH:05169 Epstein-Barr virus infection	HLA-F	0.04512629	0.6382147	2.01410856
PATH:05169 Epstein-Barr virus infection	HSPA1A	0.04512629	0.6382147	2.01410856
PATH:05169 Epstein-Barr virus infection	CDKN1A	0.04512629	0.6382147	2.01410856
PATH:05169 Epstein-Barr virus infection	GTF2E2	0.04512629	0.6382147	2.01410856
PATH:05169 Epstein-Barr virus infection	POLR2G	0.04512629	0.6382147	2.01410856
PATH:05169 Epstein-Barr virus infection	HLA-DQB1	0.04512629	0.6382147	2.01410856
PATH:05169 Epstein-Barr virus infection	POLR3D	0.04512629	0.6382147	2.01410856
PATH:05169 Epstein-Barr virus infection	NFKB2	0.04512629	0.6382147	2.01410856
PATH:05219 Bladder cancer	VEGFB	0.04951929	0.65365469	3.61460554
PATH:05219 Bladder cancer	PGF	0.04951929	0.65365469	3.61460554
PATH:05219 Bladder cancer	CDKN1A	0.04951929	0.65365469	3.61460554

Supplemental data 2 for Figure 2C

NU-DUL-1-RCHO_GSEA in PI3K/AKT pathway

NAME	PROBE	GENE SYMB	GENE_TITLE	RANK IN GE	RANK METR	RUNNING E	CORE ENRIC
row_0	PGF	null	null	14	3.75999999	0.02840132	Yes
row_1	CREB3L1	null	null	108	2	0.03925308	Yes
row_2	GNB3	null	null	148	1.88999999	0.05193948	Yes
row_3	IL2RA	null	null	180	1.77999997	0.06417253	Yes
row_4	CDKN1A	null	null	190	1.75	0.07726768	Yes
row_5	FLT1	null	null	197	1.72000003	0.09027987	Yes
row_6	FN1	null	null	250	1.62	0.10023021	Yes
row_7	EPHA2	null	null	251	1.61000001	0.11268958	Yes
row_8	FGFR1	null	null	310	1.47000003	0.12118067	Yes
row_9	EFNA1	null	null	331	1.42999995	0.13125229	Yes
row_10	FGF8	null	null	352	1.39999998	0.14109175	Yes
row_11	LAMB1	null	null	387	1.34000003	0.14977054	Yes
row_12	EIF4EBP1	null	null	413	1.30999994	0.15866481	Yes
row_13	LAMB4	null	null	463	1.25	0.16590104	Yes
row_14	THBS1	null	null	484	1.22000003	0.17434752	Yes
row_15	PDGFB	null	null	559	1.13999999	0.179489	Yes
row_16	VTN	null	null	576	1.13	0.18743795	Yes
row_17	EPOR	null	null	594	1.10000002	0.19510502	Yes
row_18	LAMB3	null	null	606	1.08000004	0.20291573	Yes
row_19	COL9A3	null	null	658	1.03999996	0.20842732	Yes
row_20	ITGB4	null	null	661	1.03999996	0.21637614	Yes
row_21	KDR	null	null	704	1	0.22202584	Yes
row_22	COL1A2	null	null	749	0.97000003	0.22734392	Yes
row_23	DDIT4	null	null	750	0.97000003	0.23485048	Yes
row_24	OSM	null	null	772	0.95999998	0.24123517	Yes
row_25	PCK2	null	null	793	0.94	0.2475148	Yes
row_26	RXRA	null	null	838	0.92000002	0.25244594	Yes
row_27	TNC	null	null	885	0.88	0.25696802	Yes
row_28	COL6A1	null	null	889	0.87	0.2635515	Yes
row_29	EFNA3	null	null	891	0.87	0.2702345	Yes
row_30	EFNA4	null	null	892	0.87	0.2769672	Yes
row_31	PPP2CB	null	null	901	0.87	0.28330198	Yes
row_32	F2R	null	null	929	0.85000002	0.28853697	Yes
row_33	LAMB2	null	null	980	0.81999999	0.29239577	Yes
row_34	FGFR2	null	null	986	0.81	0.29841545	Yes
row_35	IKBKG	null	null	987	0.81	0.30468383	Yes
row_36	GH1	null	null	999	0.80000001	0.3103277	Yes
row_37	COL6A3	null	null	1046	0.76999998	0.31399855	Yes
row_38	SGK1	null	null	1080	0.76999998	0.31831598	Yes
row_39	LPAR2	null	null	1179	0.72000003	0.31901348	Yes
row_40	COL4A6	null	null	1253	0.68000001	0.3206449	Yes
row_41	COL1A1	null	null	1326	0.66000003	0.32217124	Yes
row_42	NGFR	null	null	1384	0.63999999	0.32428893	Yes
row_43	PPP2R2A	null	null	1387	0.63999999	0.32914224	Yes
row_44	VEGFB	null	null	1507	0.60000002	0.32786655	Yes
row_45	LPAR1	null	null	1575	0.57999998	0.32902253	Yes
row_46	OSMR	null	null	1690	0.55000001	0.32760862	Yes
row_47	PPP2R5B	null	null	1732	0.54000002	0.3297482	Yes
row_48	NR4A1	null	null	1763	0.52999997	0.3323576	Yes
row_49	COL2A1	null	null	1812	0.50999999	0.33391687	Yes

row_50	COL4A3	null	null	1813	0.50999999	0.33786365	Yes
row_51	FGFR4	null	null	1822	0.50999999	0.34141248	Yes
row_52	ANGPT2	null	null	1895	0.49000001	0.34162328	Yes
row_53	IL7R	null	null	1945	0.49000001	0.34297806	Yes
row_54	LAMA3	null	null	1950	0.49000001	0.3465711	Yes
row_55	PDGFRB	null	null	1977	0.49000001	0.34906983	Yes
row_56	BAD	null	null	2057	0.47	0.34877768	Yes
row_57	COL4A1	null	null	2064	0.47	0.35211647	Yes
row_58	HRAS	null	null	2203	0.44	0.34865755	Yes
row_59	PRKCA	null	null	2221	0.44	0.35121703	Yes
row_60	ITGA3	null	null	2257	0.43000001	0.35280383	Yes
row_61	ITGA7	null	null	2310	0.41999999	0.35346767	Yes
row_62	GNG2	null	null	2367	0.41	0.35385516	Yes
row_63	EGFR	null	null	2443	0.38999999	0.35314286	Yes
row_64	IFNAR2	null	null	2453	0.38999999	0.35571334	Yes
row_65	COL9A2	null	null	2513	0.38	0.35571945	Yes
row_66	EIF4E1B	null	null	2524	0.38	0.3581628	Yes
row_67	PDGFC	null	null	2572	0.38	0.35876578	Yes
row_68	PIK3CA	null	null	2655	0.37	0.35755053	No
row_69	LAMC2	null	null	2776	0.34999999	0.35429043	No
row_70	PKN1	null	null	2908	0.33000001	0.35032842	No
row_71	FLT4	null	null	2951	0.31999999	0.3507158	No
row_72	RPS6	null	null	2984	0.31999999	0.35160053	No
row_73	HSP90B1	null	null	3018	0.31	0.35235816	No
row_74	CCNE2	null	null	3058	0.30000001	0.35273996	No
row_75	CASP9	null	null	3252	0.27000001	0.3452298	No
row_76	IRS1	null	null	3266	0.27000001	0.34667268	No
row_77	CCND3	null	null	3322	0.25999999	0.3459491	No
row_78	ITGA5	null	null	3476	0.25	0.34027374	No
row_79	CREB3	null	null	3544	0.23999999	0.33879852	No
row_80	TNXB	null	null	3606	0.23999999	0.33762175	No
row_81	CCNE1	null	null	3628	0.23	0.33835715	No
row_82	CRTC2	null	null	3632	0.23	0.33998784	No
row_83	BCL2L1	null	null	3708	0.22	0.33795995	No
row_84	FGF11	null	null	3722	0.22	0.33901587	No
row_85	MAPK3	null	null	3731	0.22	0.3403205	No
row_86	RHEB	null	null	3757	0.22	0.34077954	No
row_87	PPP2R5A	null	null	3859	0.20999999	0.33738104	No
row_88	CREB3L4	null	null	3994	0.19	0.3321864	No
row_89	VEGFA	null	null	4065	0.19	0.33017504	No
row_90	MYB	null	null	4120	0.18000001	0.3288821	No
row_91	CDK2	null	null	4163	0.17	0.32810867	No
row_92	GNG5	null	null	4181	0.17	0.32857868	No
row_93	CCND1	null	null	4254	0.16	0.32623568	No
row_94	EIF4E2	null	null	4261	0.16	0.32717547	No
row_95	YWHAZ	null	null	4314	0.16	0.32582724	No
row_96	GNB2	null	null	4346	0.15000001	0.32544613	No
row_97	PPP2R1A	null	null	4375	0.15000001	0.32521427	No
row_98	IGF1	null	null	4455	0.14	0.32236832	No
row_99	PRKAA2	null	null	4486	0.14	0.32195956	No
row_100	G6PC3	null	null	4563	0.13	0.31918544	No

row_101	STK11	null	null	4615	0.13	0.31765482	No
row_102	YWHAB	null	null	4633	0.13	0.31781527	No
row_103	IL4R	null	null	4688	0.12	0.31605804	No
row_104	ATF4	null	null	4736	0.11	0.31457156	No
row_105	PHLPP2	null	null	4905	0.1	0.3069893	No
row_106	GNG7	null	null	5109	0.08	0.29751143	No
row_107	PPP2R1B	null	null	5141	0.08	0.2965886	No
row_108	RPS6KB2	null	null	5150	0.08	0.2968098	No
row_109	CDC37	null	null	5208	0.07	0.29451638	No
row_110	CREB3L2	null	null	5215	0.07	0.29475966	No
row_111	YWHAE	null	null	5311	0.07	0.2905762	No
row_112	CDK4	null	null	5453	0.05	0.28394994	No
row_113	CDK6	null	null	5454	0.05	0.2843369	No
row_114	MLST8	null	null	5495	0.05	0.28273427	No
row_115	CREB1	null	null	5582	0.04	0.27876627	No
row_116	MAP2K2	null	null	5704	0.03	0.27298003	No
row_117	PIK3R2	null	null	5723	0.03	0.2723169	No
row_118	GNB1	null	null	5802	0.02	0.26859203	No
row_119	HSP90AA1	null	null	5806	0.02	0.2685976	No
row_120	PRKAA1	null	null	5844	0.02	0.26691204	No
row_121	ATF2	null	null	5898	0.01	0.26435325	No
row_122	ANGPT1	null	null	6192	0	0.24977978	No
row_123	ANGPT4	null	null	6193	0	0.24977978	No
row_124	CCND2	null	null	6866	0	0.21635525	No
row_125	CHAD	null	null	6998	0	0.20983945	No
row_126	CHRM1	null	null	7011	0	0.20924258	No
row_127	CHRM2	null	null	7012	0	0.20924258	No
row_128	COL4A5	null	null	7149	0	0.2024781	No
row_129	COL6A5	null	null	7151	0	0.20242836	No
row_130	COL6A6	null	null	7152	0	0.20242836	No
row_131	COL9A1	null	null	7155	0	0.20232889	No
row_132	COMP	null	null	7160	0	0.20212993	No
row_133	CREB3L3	null	null	7197	0	0.20033933	No
row_134	CSF1R	null	null	7224	0	0.19904612	No
row_135	CSF3	null	null	7227	0	0.19894664	No
row_136	CSF3R	null	null	7228	0	0.19894664	No
row_137	CSH1	null	null	7229	0	0.19894664	No
row_138	CSH2	null	null	7230	0	0.19894664	No
row_139	EFNA2	null	null	7623	0	0.179449	No
row_140	EGF	null	null	7626	0	0.17934953	No
row_141	FASLG	null	null	7856	0	0.16795932	No
row_142	FGF1	null	null	7898	0	0.16592003	No
row_143	FGF10	null	null	7899	0	0.16592003	No
row_144	FGF12	null	null	7900	0	0.16592003	No
row_145	FGF13	null	null	7901	0	0.16592003	No
row_146	FGF14	null	null	7902	0	0.16592003	No
row_147	FGF16	null	null	7903	0	0.16592003	No
row_148	FGF17	null	null	7904	0	0.16592003	No
row_149	FGF18	null	null	7905	0	0.16592003	No
row_150	FGF19	null	null	7906	0	0.16592003	No
row_151	FGF2	null	null	7907	0	0.16592003	No

row_152	FGF20	null	null	7908	0	0.16592003	No
row_153	FGF21	null	null	7909	0	0.16592003	No
row_154	FGF22	null	null	7910	0	0.16592003	No
row_155	FGF23	null	null	7911	0	0.16592003	No
row_156	FGF3	null	null	7912	0	0.16592003	No
row_157	FGF4	null	null	7913	0	0.16592003	No
row_158	FGF5	null	null	7914	0	0.16592003	No
row_159	FGF9	null	null	7915	0	0.16592003	No
row_160	FGFR3	null	null	7918	0	0.16582055	No
row_161	G6PC	null	null	8022	0	0.16069745	No
row_162	G6PC2	null	null	8023	0	0.16069745	No
row_163	GH2	null	null	8120	0	0.15592252	No
row_164	GHR	null	null	8121	0	0.15592252	No
row_165	GNG12	null	null	8173	0	0.15338583	No
row_166	GNG13	null	null	8174	0	0.15338583	No
row_167	GNG3	null	null	8175	0	0.15338583	No
row_168	GNG4	null	null	8176	0	0.15338583	No
row_169	GNG8	null	null	8177	0	0.15338583	No
row_170	GNGT1	null	null	8178	0	0.15338583	No
row_171	GYS2	null	null	8345	0	0.14512919	No
row_172	IBSP	null	null	8555	0	0.13473375	No
row_173	IFNA1	null	null	8564	0	0.13433585	No
row_174	IFNA10	null	null	8565	0	0.13433585	No
row_175	IFNA13	null	null	8566	0	0.13433585	No
row_176	IFNA14	null	null	8567	0	0.13433585	No
row_177	IFNA16	null	null	8568	0	0.13433585	No
row_178	IFNA17	null	null	8569	0	0.13433585	No
row_179	IFNA2	null	null	8570	0	0.13433585	No
row_180	IFNA21	null	null	8571	0	0.13433585	No
row_181	IFNA4	null	null	8572	0	0.13433585	No
row_182	IFNA5	null	null	8573	0	0.13433585	No
row_183	IFNA6	null	null	8574	0	0.13433585	No
row_184	IFNA7	null	null	8575	0	0.13433585	No
row_185	IFNA8	null	null	8576	0	0.13433585	No
row_186	IFNB1	null	null	8577	0	0.13433585	No
row_187	IL2	null	null	8898	0	0.11841941	No
row_188	IL2RB	null	null	8910	0	0.11787228	No
row_189	IL3	null	null	8911	0	0.11787228	No
row_190	IL3RA	null	null	8921	0	0.11742463	No
row_191	IL4	null	null	8922	0	0.11742463	No
row_192	IL7	null	null	8925	0	0.11732515	No
row_193	INS	null	null	8932	0	0.11702672	No
row_194	ITGA1	null	null	8968	0	0.11528586	No
row_195	ITGA2	null	null	8969	0	0.11528586	No
row_196	ITGA2B	null	null	8970	0	0.11528586	No
row_197	ITGA8	null	null	8971	0	0.11528586	No
row_198	ITGA9	null	null	8972	0	0.11528586	No
row_199	ITGB6	null	null	8975	0	0.11518638	No
row_200	ITGB8	null	null	8976	0	0.11518638	No
row_201	KIT	null	null	9114	0	0.10837216	No
row_202	KITLG	null	null	9115	0	0.10837216	No

row_203	LAMA1	null	null	9299	0	0.09926994	No
row_204	LAMA2	null	null	9300	0	0.09926994	No
row_205	LOC1027234	null	null	9625	0	0.08315454	No
row_206	LPAR4	null	null	9866	0	0.07121722	No
row_207	LPAR6	null	null	9867	0	0.07121722	No
row_208	NGF	null	null	10286	0	0.05042637	No
row_209	PDGFA	null	null	10976	0	0.01615629	No
row_210	PDGFD	null	null	10977	0	0.01615629	No
row_211	PDGFRA	null	null	10978	0	0.01615629	No
row_212	PIK3R6	null	null	11028	0	0.01371908	No
row_213	PPP2R2B	null	null	11152	0	0.0076012	No
row_214	PPP2R2C	null	null	11153	0	0.0076012	No
row_215	PPP2R3A	null	null	11154	0	0.0076012	No
row_216	PRL	null	null	11205	0	0.00511426	No
row_217	PRLR	null	null	11208	0	0.00501478	No
row_218	RELN	null	null	11406	0	-0.0047838	No
row_219	SGK2	null	null	11708	0	-0.0197552	No
row_220	TEK	null	null	12305	0	-0.0493995	No
row_221	THBS2	null	null	12349	0	-0.0515383	No
row_222	TLR2	null	null	12366	0	-0.0523341	No
row_223	TNN	null	null	12489	0	-0.0584023	No
row_224	TNR	null	null	12495	0	-0.058651	No
row_225	VEGFC	null	null	12968	0	-0.0821277	No
row_226	VWF	null	null	13007	0	-0.0840178	No
row_227	AKT1	null	null	13213	-0.01	-0.0941369	No
row_228	IL2RG	null	null	13248	-0.01	-0.0957506	No
row_229	RAF1	null	null	13274	-0.01	-0.0969167	No
row_230	GRB2	null	null	13350	-0.02	-0.1004923	No
row_231	PPP2R5D	null	null	13381	-0.02	-0.1018297	No
row_232	INSR	null	null	13454	-0.03	-0.1051788	No
row_233	PIK3R3	null	null	13480	-0.03	-0.1061901	No
row_234	CD19	null	null	13541	-0.04	-0.1088649	No
row_235	GSK3B	null	null	13566	-0.04	-0.109749	No
row_236	YWHAQ	null	null	13635	-0.04	-0.1128217	No
row_237	BCL2L11	null	null	13650	-0.05	-0.1131311	No
row_238	MCL1	null	null	13700	-0.05	-0.1151814	No
row_239	TP53	null	null	13875	-0.06	-0.1233717	No
row_240	PPP2R5E	null	null	13963	-0.07	-0.1271572	No
row_241	GYS1	null	null	14040	-0.08	-0.1303183	No
row_242	COL4A2	null	null	14126	-0.09	-0.1338496	No
row_243	PPP2R2D	null	null	14190	-0.09	-0.1362867	No
row_244	TCL1A	null	null	14207	-0.09	-0.136386	No
row_245	ITGB7	null	null	14263	-0.1	-0.1383478	No
row_246	MAPK1	null	null	14269	-0.1	-0.1378226	No
row_247	GNB4	null	null	14364	-0.11	-0.1416468	No
row_248	HSP90AB1	null	null	14369	-0.11	-0.1409945	No
row_249	PIK3CG	null	null	14504	-0.12	-0.1467308	No
row_250	SOS2	null	null	14520	-0.12	-0.1465483	No
row_251	ATF6B	null	null	14551	-0.13	-0.1470344	No
row_252	NFKB1	null	null	14602	-0.13	-0.1485153	No
row_253	PTEN	null	null	14610	-0.13	-0.1478574	No

row_254	ITGB5	null	null	14719	-0.14	-0.1521458	No
row_255	PPP2CA	null	null	14765	-0.14	-0.1533006	No
row_256	RAC1	null	null	14774	-0.14	-0.1526151	No
row_257	KRAS	null	null	14973	-0.16	-0.1612252	No
row_258	MYC	null	null	14982	-0.16	-0.1603849	No
row_259	GNB5	null	null	15067	-0.17	-0.1632474	No
row_260	JAK3	null	null	15072	-0.17	-0.1621308	No
row_261	PPP2R5C	null	null	15092	-0.17	-0.1617602	No
row_262	RELA	null	null	15099	-0.17	-0.1607431	No
row_263	YWHAH	null	null	15130	-0.17	-0.1609197	No
row_264	SYK	null	null	15215	-0.18	-0.1637048	No
row_265	IFNAR1	null	null	15372	-0.2	-0.1699163	No
row_266	PHLPP1	null	null	15398	-0.2	-0.169612	No
row_267	RPS6KB1	null	null	15513	-0.21	-0.1736571	No
row_268	AKT2	null	null	15545	-0.22	-0.1734965	No
row_269	CSF1	null	null	15685	-0.23	-0.1786303	No
row_270	LAMC1	null	null	15705	-0.23	-0.1777954	No
row_271	LPAR5	null	null	15707	-0.23	-0.1760652	No
row_272	BRCA1	null	null	15767	-0.24	-0.1771425	No
row_273	FGF7	null	null	16044	-0.26	-0.1888584	No
row_274	ITGA6	null	null	16068	-0.26	-0.1879903	No
row_275	ITGB3	null	null	16069	-0.26	-0.1859782	No
row_276	PIK3R5	null	null	16115	-0.26	-0.1862044	No
row_277	EFNA5	null	null	16215	-0.27	-0.1890391	No
row_278	C8orf44-SGI	null	null	16288	-0.28	-0.1904534	No
row_279	PCK1	null	null	16333	-0.28	-0.1904751	No
row_280	GNGT2	null	null	16405	-0.29	-0.1917623	No
row_281	NRAS	null	null	16419	-0.29	-0.1901647	No
row_282	EIF4B	null	null	16477	-0.3	-0.1906782	No
row_283	PIK3CB	null	null	16601	-0.31	-0.1943971	No
row_284	PKN2	null	null	16602	-0.31	-0.1919981	No
row_285	SPP1	null	null	16622	-0.31	-0.1905441	No
row_286	TSC1	null	null	16629	-0.31	-0.1884435	No
row_287	PKN3	null	null	16764	-0.33	-0.1925547	No
row_288	TSC2	null	null	16871	-0.34	-0.1951959	No
row_289	PIK3AP1	null	null	16929	-0.35	-0.1953224	No
row_290	IKBKB	null	null	16991	-0.36	-0.1955706	No
row_291	RBL2	null	null	17020	-0.36	-0.1941773	No
row_292	JAK1	null	null	17065	-0.37	-0.1935025	No
row_293	CHUK	null	null	17151	-0.38	-0.1947896	No
row_294	LPAR3	null	null	17196	-0.38	-0.1940374	No
row_295	RPTOR	null	null	17336	-0.39	-0.197933	No
row_296	BCL2	null	null	17431	-0.41	-0.1994355	No
row_297	MTCP1	null	null	17696	-0.45	-0.2090842	No
row_298	ITGAV	null	null	17816	-0.47	-0.2113659	No
row_299	PIK3R1	null	null	17877	-0.48	-0.2106356	No
row_300	HGF	null	null	17953	-0.49	-0.2105741	No
row_301	ITGB1	null	null	17962	-0.49	-0.20718	No
row_302	YWHAG	null	null	18080	-0.5	-0.2091301	No
row_303	GNG11	null	null	18105	-0.51	-0.206377	No
row_304	GNG10	null	null	18210	-0.53	-0.2074483	No

row_305	THEM4	null	null	18231	-0.53	-0.2043416	No
row_306	EIF4E	null	null	18255	-0.54	-0.2013067	No
row_307	TLR4	null	null	18283	-0.54	-0.1984707	No
row_308	CDKN1B	null	null	18296	-0.55	-0.1948113	No
row_309	SOS1	null	null	18335	-0.55	-0.192445	No
row_310	CREB5	null	null	18407	-0.57	-0.1915654	No
row_311	PDPK1	null	null	18422	-0.57	-0.1878507	No
row_312	SGK3	null	null	18622	-0.6	-0.1931055	No
row_313	LAMA5	null	null	18814	-0.65	-0.1975754	No
row_314	PIK3CD	null	null	18949	-0.68	-0.1989781	No
row_315	COL4A4	null	null	19053	-0.71	-0.1986067	No
row_316	MDM2	null	null	19146	-0.74	-0.197456	No
row_317	FOXO3	null	null	19161	-0.75	-0.1923483	No
row_318	MAP2K1	null	null	19191	-0.76	-0.1879093	No
row_319	IL6	null	null	19222	-0.77	-0.1834426	No
row_320	THBS4	null	null	19263	-0.77	-0.1794733	No
row_321	PPP2R3C	null	null	19353	-0.82	-0.1775543	No
row_322	MTOR	null	null	19449	-0.85	-0.1757016	No
row_323	PTK2	null	null	19773	-1	-0.1840285	No
row_324	ITGA4	null	null	19797	-1.02	-0.177279	No
row_325	LAMA4	null	null	19926	-1.14	-0.1748234	No
row_326	JAK2	null	null	19946	-1.15	-0.1668689	No
row_327	COL6A2	null	null	19955	-1.16	-0.1582898	No
row_328	ITGA11	null	null	19974	-1.1799999	-0.1500534	No
row_329	FGF6	null	null	19990	-1.2	-0.141513	No
row_330	MET	null	null	20011	-1.22	-0.1330665	No
row_331	EPO	null	null	20042	-1.26	-0.1248079	No
row_332	IL6R	null	null	20098	-1.35	-0.1170962	No
row_333	ITGA10	null	null	20213	-1.58	-0.1105393	No
row_334	TCL1B	null	null	20237	-1.6799999	-0.0986822	No
row_335	NOS3	null	null	20257	-1.75	-0.0860844	No
row_336	THBS3	null	null	20291	-1.87	-0.0732544	No
row_337	LAMC3	null	null	20403	-2.73	-0.0576486	No
row_338	PPP2R3B	null	null	20432	-3.75	-0.030021	No
row_339	IGF1R	null	null	20437	-3.95	3.48E-04	No

LY8-RCHO_GSEA in PI3K/AKT pathway

NAME	PROBE	GENE SYMB	GENE_TITLE	RANK IN GE	RANK METR	RUNNING E	CORE ENRIC
row_0	RXRA	null	null	28	3.53999996	0.02059003	Yes
row_1	PGF	null	null	46	3.18000007	0.03950543	Yes
row_2	EFNA1	null	null	80	2.75	0.05492203	Yes
row_3	LAMC3	null	null	84	2.74000001	0.07181921	Yes
row_4	EFNA5	null	null	136	2.36999989	0.08394532	Yes
row_5	BAD	null	null	175	2.1400001	0.09530866	Yes
row_6	IL4	null	null	195	2.06999993	0.10721349	Yes
row_7	IL7	null	null	216	1.99000001	0.11856904	Yes
row_8	FGF22	null	null	247	1.88	0.12872577	Yes
row_9	GH1	null	null	300	1.76999998	0.13706656	Yes
row_10	GNB4	null	null	338	1.69000006	0.1456809	Yes
row_11	PDGFD	null	null	385	1.59000003	0.15321007	Yes
row_12	MDM2	null	null	429	1.52999997	0.16052015	Yes
row_13	CDK6	null	null	549	1.38999999	0.16305052	Yes
row_14	PPP2R5E	null	null	553	1.38999999	0.17154643	Yes
row_15	FOXO3	null	null	593	1.35000002	0.17794205	Yes
row_16	JAK3	null	null	605	1.34000003	0.18571539	Yes
row_17	LAMB1	null	null	673	1.26999998	0.19017318	Yes
row_18	PIK3R2	null	null	730	1.21000004	0.1948233	Yes
row_19	ITGB4	null	null	776	1.17999995	0.1998524	Yes
row_20	IFNB1	null	null	854	1.12	0.20286246	Yes
row_21	LPAR3	null	null	899	1.10000002	0.20744514	Yes
row_22	SGK1	null	null	904	1.10000002	0.21408492	Yes
row_23	IGF1R	null	null	960	1.07000005	0.2179152	Yes
row_24	COL9A1	null	null	1028	1.02999997	0.22087945	Yes
row_25	BCL2L11	null	null	1044	1.01999998	0.22645567	Yes
row_26	COL9A2	null	null	1071	1.00999999	0.23140396	Yes
row_27	FGF18	null	null	1085	1	0.23695856	Yes
row_28	IL7R	null	null	1086	1	0.24318174	Yes
row_29	PIK3CA	null	null	1109	0.99000001	0.24821126	Yes
row_30	IL2RA	null	null	1126	0.98000002	0.25348714	Yes
row_31	LAMB3	null	null	1215	0.94	0.25481132	Yes
row_32	ITGAV	null	null	1234	0.93000001	0.25967318	Yes
row_33	CCNE2	null	null	1399	0.86000001	0.25659105	Yes
row_34	MET	null	null	1528	0.81999999	0.25511137	Yes
row_35	GNG8	null	null	1619	0.79000002	0.25539923	Yes
row_36	PPP2R2A	null	null	1797	0.74000001	0.25090176	Yes
row_37	GSK3B	null	null	1819	0.73000002	0.2543647	Yes
row_38	NOS3	null	null	1828	0.73000002	0.2584962	Yes
row_39	ATF2	null	null	1844	0.72000003	0.26220548	Yes
row_40	PKN1	null	null	1939	0.69999999	0.26172754	Yes
row_41	YWHAZ	null	null	1985	0.69	0.2637073	Yes
row_42	COL9A3	null	null	2035	0.67000002	0.2653569	Yes
row_43	ITGA2	null	null	2076	0.66000003	0.2674071	Yes
row_44	PPP2CB	null	null	2087	0.66000003	0.27100012	Yes
row_45	MLST8	null	null	2117	0.64999998	0.2735538	Yes
row_46	CSF1	null	null	2152	0.63999999	0.27578807	Yes
row_47	LPAR5	null	null	2169	0.63999999	0.27894807	Yes
row_48	ITGA3	null	null	2209	0.63	0.28086302	Yes
row_49	ITGB5	null	null	2210	0.63	0.2847836	Yes

row_50	KRAS	null	null	2255	0.62	0.28637916	Yes
row_51	VEGFB	null	null	2341	0.61000001	0.28580397	Yes
row_52	BCL2	null	null	2349	0.60000002	0.2891779	Yes
row_53	SGK3	null	null	2415	0.58999997	0.2895068	Yes
row_54	CREB3L2	null	null	2428	0.57999998	0.2924991	Yes
row_55	EIF4B	null	null	2566	0.55000001	0.28887632	Yes
row_56	GNB5	null	null	2572	0.55000001	0.29204193	Yes
row_57	MAPK1	null	null	2580	0.55000001	0.29510468	Yes
row_58	IRS1	null	null	2713	0.51999998	0.29155236	Yes
row_59	PIK3R5	null	null	2720	0.51999998	0.29447985	Yes
row_60	C8orf44-SGI	null	null	2738	0.50999999	0.2967794	Yes
row_61	PDPK1	null	null	2765	0.50999999	0.29861608	Yes
row_62	EIF4EBP1	null	null	2797	0.5	0.30013344	Yes
row_63	SOS2	null	null	2916	0.47999999	0.29705215	Yes
row_64	FGF17	null	null	2950	0.47	0.29827994	Yes
row_65	G6PC3	null	null	2951	0.47	0.30120483	Yes
row_66	NRAS	null	null	2970	0.47	0.30320403	Yes
row_67	CREB3L3	null	null	3001	0.46000001	0.3045239	Yes
row_68	LAMC1	null	null	3081	0.44999999	0.30326155	Yes
row_69	GNB2	null	null	3128	0.44	0.3036341	Yes
row_70	PHLPP2	null	null	3140	0.44	0.3058066	Yes
row_71	PDGFA	null	null	3209	0.43000001	0.30498552	Yes
row_72	THBS3	null	null	3224	0.43000001	0.30694148	Yes
row_73	IL2RG	null	null	3255	0.41999999	0.3080124	Yes
row_74	SOS1	null	null	3427	0.38999999	0.3016454	No
row_75	IKBKB	null	null	3470	0.38	0.30185026	No
row_76	COL4A3	null	null	3522	0.37	0.30153006	No
row_77	PIK3CB	null	null	3550	0.37	0.3024441	No
row_78	TP53	null	null	3571	0.37	0.30371812	No
row_79	COL6A2	null	null	3595	0.36000001	0.30477563	No
row_80	CSF3R	null	null	3596	0.36000001	0.307016	No
row_81	YWHAG	null	null	3647	0.36000001	0.30668497	No
row_82	LAMA4	null	null	3746	0.34	0.30376098	No
row_83	MYC	null	null	3899	0.31999999	0.29793546	No
row_84	PIK3AP1	null	null	3910	0.31999999	0.2994126	No
row_85	F2R	null	null	3948	0.31	0.29943898	No
row_86	G6PC	null	null	3952	0.31	0.3012139	No
row_87	HSP90AA1	null	null	3955	0.31	0.3030402	No
row_88	CREB5	null	null	3999	0.30000001	0.30269578	No
row_89	RELA	null	null	4035	0.30000001	0.3027628	No
row_90	STK11	null	null	4045	0.30000001	0.3041669	No
row_91	RPS6KB1	null	null	4117	0.28999999	0.3023203	No
row_92	NFKB1	null	null	4179	0.28	0.30092573	No
row_93	PPP2R1B	null	null	4184	0.28	0.3024625	No
row_94	ANGPT2	null	null	4344	0.25	0.2958414	No
row_95	CREB1	null	null	4354	0.25	0.29693434	No
row_96	LAMA5	null	null	4368	0.25	0.29782158	No
row_97	MAP2K1	null	null	4444	0.23999999	0.2954581	No
row_98	PHLPP1	null	null	4457	0.23999999	0.29633453	No
row_99	PTK2	null	null	4531	0.23	0.29401168	No
row_100	JAK1	null	null	4736	0.2	0.28476518	No

row_101	PKN2	null	null	4749	0.2	0.2853927	No
row_102	MYB	null	null	4803	0.19	0.28384945	No
row_103	BRCA1	null	null	4837	0.18000001	0.28327253	No
row_104	IGF1	null	null	4851	0.18000001	0.28372413	No
row_105	AKT2	null	null	4897	0.17	0.28246787	No
row_106	CRTC2	null	null	4912	0.17	0.28280583	No
row_107	PIK3CG	null	null	5112	0.15000001	0.2735053	No
row_108	RPS6KB2	null	null	5118	0.15000001	0.27418163	No
row_109	TSC2	null	null	5283	0.13	0.26655662	No
row_110	CDC37	null	null	5295	0.12	0.2667377	No
row_111	GYS1	null	null	5362	0.11	0.26402804	No
row_112	TNXB	null	null	5462	0.1	0.25955907	No
row_113	CHUK	null	null	5494	0.09	0.25852492	No
row_114	SYK	null	null	5636	0.08	0.25177157	No
row_115	RPTOR	null	null	5721	0.07	0.2478873	No
row_116	ATF6B	null	null	5847	0.05	0.24177007	No
row_117	IL4R	null	null	5876	0.05	0.24064127	No
row_118	EFNA4	null	null	5970	0.04	0.23610747	No
row_119	COL1A1	null	null	6056	0.03	0.23192286	No
row_120	LAMB2	null	null	6080	0.03	0.23092674	No
row_121	SGK2	null	null	6107	0.03	0.22977632	No
row_122	ANGPT1	null	null	6514	0	0.20889692	No
row_123	ANGPT4	null	null	6515	0	0.20889692	No
row_124	CCND1	null	null	7153	0	0.17613786	No
row_125	CHAD	null	null	7283	0	0.16950376	No
row_126	CHRM1	null	null	7291	0	0.16914378	No
row_127	CHRM2	null	null	7292	0	0.16914378	No
row_128	COL1A2	null	null	7410	0	0.16312681	No
row_129	COL2A1	null	null	7414	0	0.16297252	No
row_130	COL4A1	null	null	7416	0	0.1629211	No
row_131	COL4A2	null	null	7417	0	0.1629211	No
row_132	COL4A5	null	null	7418	0	0.1629211	No
row_133	COL4A6	null	null	7419	0	0.1629211	No
row_134	COL6A1	null	null	7422	0	0.16281824	No
row_135	COL6A5	null	null	7423	0	0.16281824	No
row_136	COL6A6	null	null	7424	0	0.16281824	No
row_137	CSF1R	null	null	7491	0	0.15942405	No
row_138	CSF3	null	null	7494	0	0.1593212	No
row_139	CSH1	null	null	7495	0	0.1593212	No
row_140	CSH2	null	null	7496	0	0.1593212	No
row_141	EGF	null	null	7878	0	0.13972747	No
row_142	EGFR	null	null	7881	0	0.13962463	No
row_143	EPO	null	null	7937	0	0.13679613	No
row_144	FASLG	null	null	8093	0	0.12882493	No
row_145	FGF1	null	null	8135	0	0.12671642	No
row_146	FGF10	null	null	8136	0	0.12671642	No
row_147	FGF12	null	null	8137	0	0.12671642	No
row_148	FGF13	null	null	8138	0	0.12671642	No
row_149	FGF14	null	null	8139	0	0.12671642	No
row_150	FGF16	null	null	8140	0	0.12671642	No
row_151	FGF19	null	null	8141	0	0.12671642	No

row_152	FGF2	null	null	8142	0	0.12671642	No
row_153	FGF20	null	null	8143	0	0.12671642	No
row_154	FGF21	null	null	8144	0	0.12671642	No
row_155	FGF23	null	null	8145	0	0.12671642	No
row_156	FGF3	null	null	8146	0	0.12671642	No
row_157	FGF4	null	null	8147	0	0.12671642	No
row_158	FGF5	null	null	8148	0	0.12671642	No
row_159	FGF6	null	null	8149	0	0.12671642	No
row_160	FGF7	null	null	8150	0	0.12671642	No
row_161	FGF9	null	null	8151	0	0.12671642	No
row_162	FGFR2	null	null	8154	0	0.12661356	No
row_163	FGFR3	null	null	8155	0	0.12661356	No
row_164	G6PC2	null	null	8257	0	0.12141943	No
row_165	GH2	null	null	8357	0	0.11632814	No
row_166	GHR	null	null	8358	0	0.11632814	No
row_167	GNB3	null	null	8416	0	0.1133968	No
row_168	GNG13	null	null	8417	0	0.1133968	No
row_169	GNG3	null	null	8418	0	0.1133968	No
row_170	GNG4	null	null	8419	0	0.1133968	No
row_171	GNGT1	null	null	8420	0	0.1133968	No
row_172	IBSP	null	null	8779	0	0.0949859	No
row_173	IFNA1	null	null	8790	0	0.09447163	No
row_174	IFNA10	null	null	8791	0	0.09447163	No
row_175	IFNA13	null	null	8792	0	0.09447163	No
row_176	IFNA14	null	null	8793	0	0.09447163	No
row_177	IFNA16	null	null	8794	0	0.09447163	No
row_178	IFNA17	null	null	8795	0	0.09447163	No
row_179	IFNA2	null	null	8796	0	0.09447163	No
row_180	IFNA21	null	null	8797	0	0.09447163	No
row_181	IFNA4	null	null	8798	0	0.09447163	No
row_182	IFNA5	null	null	8799	0	0.09447163	No
row_183	IFNA6	null	null	8800	0	0.09447163	No
row_184	IFNA7	null	null	8801	0	0.09447163	No
row_185	IFNA8	null	null	8802	0	0.09447163	No
row_186	IL2	null	null	8849	0	0.09210598	No
row_187	IL2RB	null	null	8860	0	0.09159171	No
row_188	IL3	null	null	8861	0	0.09159171	No
row_189	IL3RA	null	null	8871	0	0.09112886	No
row_190	INS	null	null	8881	0	0.09066602	No
row_191	ITGA1	null	null	8917	0	0.08886607	No
row_192	ITGA6	null	null	8918	0	0.08886607	No
row_193	ITGA8	null	null	8919	0	0.08886607	No
row_194	ITGA9	null	null	8920	0	0.08886607	No
row_195	ITGB6	null	null	8921	0	0.08886607	No
row_196	ITGB8	null	null	8922	0	0.08886607	No
row_197	KDR	null	null	9016	0	0.08408335	No
row_198	KIT	null	null	9063	0	0.08171771	No
row_199	KITLG	null	null	9064	0	0.08171771	No
row_200	LAMA1	null	null	9254	0	0.07199799	No
row_201	LAMA2	null	null	9255	0	0.07199799	No
row_202	LAMC2	null	null	9256	0	0.07199799	No

row_203	LPAR1	null	null	9765	0	0.04587302	No
row_204	LPAR4	null	null	9766	0	0.04587302	No
row_205	LPAR6	null	null	9767	0	0.04587302	No
row_206	NGF	null	null	10175	0	0.02494218	No
row_207	OSMR	null	null	10743	0	-0.004217	No
row_208	PCK1	null	null	10856	0	-0.0099768	No
row_209	PDGFC	null	null	10875	0	-0.0109025	No
row_210	PDGFRA	null	null	10876	0	-0.0109025	No
row_211	PDGFRB	null	null	10877	0	-0.0109025	No
row_212	PIK3R6	null	null	10933	0	-0.013731	No
row_213	PPP2R2B	null	null	11067	0	-0.0205708	No
row_214	PPP2R2C	null	null	11068	0	-0.0205708	No
row_215	PPP2R3A	null	null	11069	0	-0.0205708	No
row_216	PPP2R3B	null	null	11070	0	-0.0205708	No
row_217	PRKAA2	null	null	11114	0	-0.0227822	No
row_218	PRL	null	null	11122	0	-0.0231422	No
row_219	PRLR	null	null	11125	0	-0.023245	No
row_220	RELN	null	null	11322	0	-0.0333247	No
row_221	TEK	null	null	12150	0	-0.0758549	No
row_222	THBS1	null	null	12191	0	-0.077912	No
row_223	THBS2	null	null	12192	0	-0.077912	No
row_224	TNC	null	null	12302	0	-0.0835176	No
row_225	TNN	null	null	12317	0	-0.0842376	No
row_226	TNR	null	null	12323	0	-0.0844947	No
row_227	VEGFC	null	null	12554	0	-0.0963229	No
row_228	VTN	null	null	12582	0	-0.0977115	No
row_229	VWF	null	null	12589	0	-0.09802	No
row_230	AKT1	null	null	12763	-0.01	-0.1068547	No
row_231	GNG10	null	null	12786	-0.01	-0.1079238	No
row_232	INSR	null	null	12789	-0.01	-0.1079645	No
row_233	YWHAH	null	null	12899	-0.02	-0.1134456	No
row_234	PIK3R1	null	null	12953	-0.03	-0.1159845	No
row_235	EFNA3	null	null	13002	-0.04	-0.1182041	No
row_236	EPOR	null	null	13086	-0.05	-0.1221614	No
row_237	IFNAR1	null	null	13092	-0.05	-0.1221073	No
row_238	PPP2R5A	null	null	13120	-0.05	-0.1231847	No
row_239	PTEN	null	null	13205	-0.06	-0.1271312	No
row_240	MAP2K2	null	null	13263	-0.07	-0.1296269	No
row_241	RAC1	null	null	13275	-0.07	-0.129757	No
row_242	RAF1	null	null	13276	-0.07	-0.1293214	No
row_243	PDGFB	null	null	13345	-0.08	-0.1323206	No
row_244	YWHAB	null	null	13609	-0.11	-0.1451614	No
row_245	BCL2L1	null	null	13627	-0.12	-0.1452888	No
row_246	CASP9	null	null	13793	-0.14	-0.1529031	No
row_247	ITGA5	null	null	13811	-0.14	-0.1529061	No
row_248	YWHAQ	null	null	13858	-0.14	-0.1544005	No
row_249	MTOR	null	null	13893	-0.15	-0.1552155	No
row_250	GNGT2	null	null	13954	-0.16	-0.1573055	No
row_251	ITGB7	null	null	13958	-0.16	-0.156464	No
row_252	JAK2	null	null	14361	-0.21	-0.1758309	No
row_253	PPP2R2D	null	null	14456	-0.22	-0.1792959	No

row_254	LAMB4	null	null	14510	-0.23	-0.1805902	No
row_255	LPAR2	null	null	14512	-0.23	-0.1792103	No
row_256	PRKAA1	null	null	14530	-0.23	-0.1786532	No
row_257	RBL2	null	null	14532	-0.23	-0.1772734	No
row_258	THEM4	null	null	14544	-0.23	-0.1764077	No
row_259	ITGA10	null	null	14599	-0.24	-0.1776912	No
row_260	PPP2R1A	null	null	14794	-0.26	-0.1860501	No
row_261	CDK4	null	null	14835	-0.27	-0.1864269	No
row_262	FN1	null	null	14936	-0.28	-0.1898271	No
row_263	PIK3CD	null	null	15098	-0.3	-0.1962399	No
row_264	FGF11	null	null	15155	-0.31	-0.1971907	No
row_265	CREB3	null	null	15278	-0.33	-0.2014111	No
row_266	CREB3L4	null	null	15372	-0.34	-0.204078	No
row_267	PKN3	null	null	15400	-0.34	-0.2033506	No
row_268	PPP2R5B	null	null	15402	-0.34	-0.2012862	No
row_269	GRB2	null	null	15462	-0.35	-0.2021423	No
row_270	CCND2	null	null	15510	-0.36	-0.202319	No
row_271	CDK2	null	null	15586	-0.37	-0.2038735	No
row_272	YWHAE	null	null	15714	-0.38	-0.2080399	No
row_273	SPP1	null	null	15778	-0.39	-0.2088528	No
row_274	LAMA3	null	null	15821	-0.4	-0.2085235	No
row_275	TSC1	null	null	15852	-0.4	-0.207577	No
row_276	COL4A4	null	null	15865	-0.41	-0.2056426	No
row_277	PRKCA	null	null	15950	-0.42	-0.2073488	No
row_278	FGFR4	null	null	16064	-0.44	-0.2104219	No
row_279	COMP	null	null	16193	-0.46	-0.2141419	No
row_280	ITGB3	null	null	16210	-0.46	-0.212102	No
row_281	EIF4E2	null	null	16260	-0.47	-0.2116971	No
row_282	PPP2R5D	null	null	16277	-0.47	-0.209595	No
row_283	HSP90AB1	null	null	16332	-0.48	-0.209385	No
row_284	IFNAR2	null	null	16334	-0.48	-0.2064493	No
row_285	CCNE1	null	null	16523	-0.51	-0.2129438	No
row_286	GNG12	null	null	16545	-0.51	-0.2108499	No
row_287	COL6A3	null	null	16598	-0.52	-0.2102881	No
row_288	EFNA2	null	null	16601	-0.52	-0.2071549	No
row_289	EIF4E	null	null	16645	-0.53	-0.206068	No
row_290	GYS2	null	null	16651	-0.53	-0.2030269	No
row_291	MCL1	null	null	16656	-0.53	-0.1999343	No
row_292	EPHA2	null	null	16745	-0.55	-0.2010371	No
row_293	CD19	null	null	16870	-0.57	-0.2038669	No
row_294	PPP2CA	null	null	16901	-0.57	-0.2018625	No
row_295	PPP2R3C	null	null	16944	-0.58	-0.200413	No
row_296	PPP2R5C	null	null	17057	-0.6	-0.202439	No
row_297	HSP90B1	null	null	17106	-0.61	-0.2011113	No
row_298	CDKN1A	null	null	17148	-0.62	-0.1993615	No
row_299	RPS6	null	null	17178	-0.62	-0.1969945	No
row_300	MAPK3	null	null	17218	-0.63	-0.1950796	No
row_301	CREB3L1	null	null	17307	-0.65	-0.1955601	No
row_302	CCND3	null	null	17606	-0.71	-0.2064669	No
row_303	ITGA11	null	null	17617	-0.71	-0.2025628	No
row_304	IL6	null	null	17654	-0.72	-0.1999334	No

row_305	ITGB1	null	null	18061	-0.83	-0.2156476	No
row_306	GNG11	null	null	18144	-0.86	-0.2145127	No
row_307	NR4A1	null	null	18157	-0.86	-0.2097779	No
row_308	EIF4E1B	null	null	18401	-0.95	-0.2163627	No
row_309	ATF4	null	null	18417	-0.96	-0.2111599	No
row_310	FLT4	null	null	18424	-0.96	-0.2054942	No
row_311	GNB1	null	null	18477	-0.98	-0.2020697	No
row_312	MTCP1	null	null	18485	-0.98	-0.196331	No
row_313	ITGA4	null	null	18617	-1.05	-0.1965337	No
row_314	VEGFA	null	null	18696	-1.08	-0.193824	No
row_315	OSM	null	null	18712	-1.09	-0.1878121	No
row_316	TLR4	null	null	18715	-1.09	-0.1811317	No
row_317	FLT1	null	null	18763	-1.12	-0.1765789	No
row_318	PCK2	null	null	18797	-1.13	-0.1712438	No
row_319	GNG7	null	null	18810	-1.14	-0.1647665	No
row_320	IL6R	null	null	18943	-1.22	-0.1639626	No
row_321	THBS4	null	null	18978	-1.24	-0.1579944	No
row_322	PIK3R3	null	null	19000	-1.26	-0.1512332	No
row_323	GNG5	null	null	19023	-1.28	-0.1443989	No
row_324	CDKN1B	null	null	19123	-1.36	-0.1410267	No
row_325	ITGA2B	null	null	19167	-1.41	-0.1344634	No
row_326	ITGA7	null	null	19175	-1.42	-0.1259865	No
row_327	RHEB	null	null	19176	-1.42	-0.1171496	No
row_328	TCL1A	null	null	19211	-1.47	-0.1097501	No
row_329	GNG2	null	null	19342	-1.66	-0.1061052	No
row_330	IKBKG	null	null	19351	-1.67	-0.0961239	No
row_331	NGFR	null	null	19369	-1.72	-0.0862944	No
row_332	HRAS	null	null	19468	-1.91	-0.079448	No
row_333	FGF8	null	null	19500	-2.01	-0.0685337	No
row_334	TLR2	null	null	19624	-2.48	-0.0594257	No
row_335	HGF	null	null	19625	-2.49	-0.0439301	No
row_336	DDIT4	null	null	19632	-2.53	-0.028494	No
row_337	TCL1B	null	null	19658	-2.6900001	-0.0130394	No
row_338	FGFR1	null	null	19705	-3.1199999	0.00401121	No

Supplementary Data 3 for Figure 3A

Genes	Group
CREB3L1	NU-DUL-1-RCHO
GNB3	NU-DUL-1-RCHO
CDKN1A	NU-DUL-1-RCHO
FLT1	NU-DUL-1-RCHO
FN1	NU-DUL-1-RCHO
EPHA2	NU-DUL-1-RCHO
FGFR1	NU-DUL-1-RCHO
FGF8	NU-DUL-1-RCHO
LAMB4	NU-DUL-1-RCHO
THBS1	NU-DUL-1-RCHO
PDGFB	NU-DUL-1-RCHO
VTN	NU-DUL-1-RCHO
EPOR	NU-DUL-1-RCHO
KDR	NU-DUL-1-RCHO
COL1A2	NU-DUL-1-RCHO
DDIT4	NU-DUL-1-RCHO
OSM	NU-DUL-1-RCHO
PCK2	NU-DUL-1-RCHO
TNC	NU-DUL-1-RCHO
COL6A1	NU-DUL-1-RCHO
EFNA3	NU-DUL-1-RCHO
EFNA4	NU-DUL-1-RCHO
F2R	NU-DUL-1-RCHO
LAMB2	NU-DUL-1-RCHO
FGFR2	NU-DUL-1-RCHO
IKBKG	NU-DUL-1-RCHO
COL6A3	NU-DUL-1-RCHO
LPAR2	NU-DUL-1-RCHO
COL4A6	NU-DUL-1-RCHO
COL1A1	NU-DUL-1-RCHO
NGFR	NU-DUL-1-RCHO
LPAR1	NU-DUL-1-RCHO
OSMR	NU-DUL-1-RCHO
PPP2R5B	NU-DUL-1-RCHO
NR4A1	NU-DUL-1-RCHO
COL2A1	NU-DUL-1-RCHO
COL4A3	NU-DUL-1-RCHO
FGFR4	NU-DUL-1-RCHO
ANGPT2	NU-DUL-1-RCHO
LAMA3	NU-DUL-1-RCHO
PDGFRB	NU-DUL-1-RCHO
COL4A1	NU-DUL-1-RCHO
HRAS	NU-DUL-1-RCHO
PRKCA	NU-DUL-1-RCHO
ITGA7	NU-DUL-1-RCHO
GNG2	NU-DUL-1-RCHO
EGFR	NU-DUL-1-RCHO
IFNAR2	NU-DUL-1-RCHO
EIF4E1B	NU-DUL-1-RCHO
PDGFC	NU-DUL-1-RCHO
PGF	BOTH
IL2RA	BOTH
EFNA1	BOTH
LAMB1	BOTH
EIF4EBP1	BOTH
LAMB3	BOTH
COL9A3	BOTH
ITGB4	BOTH
RXRA	BOTH
PPP2CB	BOTH
GH1	BOTH
SGK1	BOTH

PPP2R2A	BOTH
VEGFB	BOTH
IL7R	BOTH
BAD	BOTH
ITGA3	BOTH
COL9A2	BOTH
LAMC3	LY8-RCHO
EFNA5	LY8-RCHO
IL4	LY8-RCHO
IL7	LY8-RCHO
FGF22	LY8-RCHO
GNB4	LY8-RCHO
PDGFD	LY8-RCHO
MDM2	LY8-RCHO
CDK6	LY8-RCHO
PPP2R5E	LY8-RCHO
FOXO3	LY8-RCHO
JAK3	LY8-RCHO
PIK3R2	LY8-RCHO
IFNB1	LY8-RCHO
LPAR3	LY8-RCHO
IGF1R	LY8-RCHO
COL9A1	LY8-RCHO
BCL2L11	LY8-RCHO
FGF18	LY8-RCHO
PIK3CA	LY8-RCHO
ITGAV	LY8-RCHO
CCNE2	LY8-RCHO
MET	LY8-RCHO
GNG8	LY8-RCHO
GSK3B	LY8-RCHO
NOS3	LY8-RCHO
ATF2	LY8-RCHO
PKN1	LY8-RCHO
YWHAZ	LY8-RCHO
ITGA2	LY8-RCHO
MLST8	LY8-RCHO
CSF1	LY8-RCHO
LPAR5	LY8-RCHO
ITGB5	LY8-RCHO
KRAS	LY8-RCHO
BCL2	LY8-RCHO
SGK3	LY8-RCHO
CREB3L2	LY8-RCHO
EIF4B	LY8-RCHO
GNB5	LY8-RCHO
MAPK1	LY8-RCHO
IRS1	LY8-RCHO
PIK3R5	LY8-RCHO
C8orf44-SGI	LY8-RCHO
PDPK1	LY8-RCHO
SOS2	LY8-RCHO
FGF17	LY8-RCHO
G6PC3	LY8-RCHO
NRAS	LY8-RCHO
CREB3L3	LY8-RCHO
LAMC1	LY8-RCHO
GNB2	LY8-RCHO
PHLPP2	LY8-RCHO
PDGFA	LY8-RCHO
THBS3	LY8-RCHO
IL2RG	LY8-RCHO

Supplemental data 3 for Figure 3B

Category	Term	Count	%	PValue	Genes	List Total	Pop Hits	Pop Total	Fold Enrichr	Bonferroni	Benjamini	FDR
KEGG_PATHWAY	hsa04151:PI3K-Akt signaling pathway	121	97.5806452	2.72E-161	FGF18, HRAS, PPP2R5B, PDGI	124	345	6910	19.5444133	4.68E-159	4.68E-159	3.33E-158
KEGG_PATHWAY	hsa04510:Focal adhesion	49	39.516129	1.23E-42	HRAS, PDGFB, PDGFA, PGF, T	124	206	6910	13.2551676	2.11E-40	1.06E-40	1.50E-39
KEGG_PATHWAY	hsa04012:ErbB signaling pathway	14	11.2903226	3.20E-09	PRKCA, EGFR, HRAS, BAD, NI	124	87	6910	8.96737115	5.50E-07	2.39E-08	3.91E-06
KEGG_PATHWAY	hsa05200:Pathways in cancer	57	45.9677419	6.03E-38	FGF18, HRAS, FGF8, PDGFB, F	124	393	6910	8.08236887	1.04E-35	3.46E-36	7.37E-35
KEGG_PATHWAY	hsa04662:B cell receptor signaling pathway	10	8.06451613	3.09E-06	MAPK1, NRAS, HRAS, KRAS, (	124	69	6910	8.07620383	5.31E-04	1.08E-05	0.00377188
KEGG_PATHWAY	hsa04066:HIF-1 signaling pathway	14	11.2903226	1.42E-08	PRKCA, EGFR, FLT1, EIF4E1B,	124	98	6910	7.96082949	2.45E-06	8.75E-08	1.74E-05
KEGG_PATHWAY	hsa04062:Chemokine signaling pathway	18	14.516129	2.42E-08	HRAS, FOXO3, GNG8, NRAS,	124	186	6910	5.39281998	4.17E-06	1.23E-07	2.96E-05
KEGG_PATHWAY	hsa04668:TNF signaling pathway	10	8.06451613	1.03E-04	MAPK1, CSF1, IKBKG, CREB3L	124	106	6910	5.25715155	0.0175437	2.95E-04	0.12570944
KEGG_PATHWAY	hsa04550:Signaling pathways regulating pluripotency of stem cells	13	10.483871	6.27E-06	FGFR2, FGFR1, FGFR4, HRAS,	124	140	6910	5.17453917	0.00107826	2.07E-05	0.00766696
KEGG_PATHWAY	hsa04060:Cytokine-cytokine receptor interaction	21	16.9354839	2.94E-09	EGFR, IL4, FLT1, IL2RA, PDGFI	124	230	6910	5.08800842	5.05E-07	2.30E-08	3.59E-06
KEGG_PATHWAY	hsa05162:Measles	12	9.67741935	2.19E-05	IL4, CCNE2, IFNAR2, IL2RA, IF	124	133	6910	5.02789231	0.00375301	6.84E-05	0.02671896
KEGG_PATHWAY	hsa04650:Natural killer cell mediated cytotoxicity	11	8.87096774	5.67E-05	PRKCA, MAPK1, NRAS, IFNAI	124	122	6910	5.02445796	0.00971264	1.68E-04	0.06934043
KEGG_PATHWAY	hsa04916:Melanogenesis	9	7.25806452	3.80E-04	PRKCA, MAPK1, NRAS, HRAS	124	100	6910	5.01532258	0.06328955	0.00105398	0.46358075
KEGG_PATHWAY	hsa04360:Axon guidance	11	8.87096774	7.99E-05	MAPK1, NRAS, HRAS, KRAS, I	124	127	6910	4.82664465	0.01364727	2.33E-04	0.09761043
KEGG_PATHWAY	hsa05034:Alcoholism	15	12.0967742	2.65E-06	HRAS, ATF2, GNG8, NRAS, M.	124	177	6910	4.72252597	4.55E-04	9.68E-06	0.00323452
KEGG_PATHWAY	hsa05412:Arrhythmogenic right ventricular cardiomyopathy (ARVC)	6	4.83870968	0.00841038	ITGAV, ITGA7, ITGB4, ITGB5, I	124	71	6910	4.70922308	0.76606305	0.01845204	9.80918848
KEGG_PATHWAY	hsa04713:Circadian entrainment	8	6.4516129	0.00144317	PRKCA, GNG8, MAPK1, GNB2	124	95	6910	4.69269949	0.21995622	0.00364635	1.7499067
KEGG_PATHWAY	hsa04727:GABAergic synapse	7	5.64516129	0.00391429	PRKCA, GNG8, GNB2, GNB5,	124	85	6910	4.58918406	0.49062934	0.00919825	4.68108153
KEGG_PATHWAY	hsa05030:Cocaine addiction	4	3.22580645	0.05610101	CREB3L2, CREB3L1, CREB3L3,	124	49	6910	4.54904542	0.99995134	0.09544233	50.626763
KEGG_PATHWAY	hsa04723:Retrograde endocannabinoid signaling	8	6.4516129	0.0020577	PRKCA, GNG8, MAPK1, GNB2	124	101	6910	4.41392526	0.29832809	0.00497756	2.48647462
KEGG_PATHWAY	hsa04010:MAPK signaling pathway	20	16.1290323	9.45E-08	PRKCA, EGFR, FGFR2, FGFR1,	124	255	6910	4.37065149	1.63E-05	4.07E-07	1.16E-04
KEGG_PATHWAY	hsa04611:Platelet activation	10	8.06451613	4.82E-04	MAPK1, COL1A2, PIK3CA, ITG	124	130	6910	4.2866005	0.0796475	0.00129601	0.58812849
KEGG_PATHWAY	hsa05142:Chagas disease (American trypanosomiasis)	8	6.4516129	0.00243253	MAPK1, IFNB1, PPP2CB, IKBK	124	104	6910	4.2866005	0.3422333	0.00580123	2.93324839
KEGG_PATHWAY	hsa05032:Morphine addiction	7	5.64516129	0.00547316	PRKCA, GNG8, GNB2, GNB5,	124	91	6910	4.2866005	0.61091988	0.01234384	6.48864251
KEGG_PATHWAY	hsa04912:GnRH signaling pathway	7	5.64516129	0.00547316	PRKCA, EGFR, MAPK1, NRAS,	124	91	6910	4.2866005	0.61091988	0.01234384	6.48864251
KEGG_PATHWAY	hsa05410:Hypertrophic cardiomyopathy (HCM)	6	4.83870968	0.01236366	ITGAV, ITGA7, ITGB4, ITGB5, I	124	78	6910	4.2866005	0.88232216	0.02515214	14.1075635
KEGG_PATHWAY	hsa05031:Amphetamine addiction	5	4.03225806	0.02954577	PRKCA, CREB3L2, CREB3L1, C	124	66	6910	4.221652	0.99424945	0.05570458	30.6918784
KEGG_PATHWAY	hsa04720:Long-term potentiation	5	4.03225806	0.02954577	PRKCA, MAPK1, NRAS, HRAS	124	66	6910	4.221652	0.99424945	0.05570458	30.6918784
KEGG_PATHWAY	hsa04115:p53 signaling pathway	5	4.03225806	0.03100273	CCNE2, CDKN1A, MDM2, CDI	124	67	6910	4.15864227	0.99555902	0.05717927	31.9531543
KEGG_PATHWAY	hsa04925:Aldosterone synthesis and secretion	6	4.83870968	0.01438915	PRKCA, CREB3L2, CREB3L1, N	124	81	6910	4.12783751	0.91733121	0.02857126	16.2362282
KEGG_PATHWAY	hsa05414:Dilated cardiomyopathy	6	4.83870968	0.01662684	ITGAV, ITGA7, ITGB4, ITGB5, I	124	84	6910	3.98041475	0.94408143	0.03260443	18.5315065
KEGG_PATHWAY	hsa04918:Thyroid hormone synthesis	5	4.03225806	0.03562472	PRKCA, CREB3L2, CREB3L1, C	124	70	6910	3.98041475	0.9980487	0.06422022	35.8162326
KEGG_PATHWAY	hsa04920:Adipocytokine signaling pathway	5	4.03225806	0.03562472	RXRA, IKBKG, PCK2, IRS1, G6I	124	70	6910	3.98041475	0.9980487	0.06422022	35.8162326
KEGG_PATHWAY	hsa04923:Regulation of lipolysis in adipocytes	4	3.22580645	0.07729585	PIK3CA, PIK3R5, IRS1, PIK3R2	124	56	6910	3.98041475	0.99999902	0.12570554	62.5952719
KEGG_PATHWAY	hsa04520:Adherens junction	5	4.03225806	0.03724946	EGFR, MAPK1, IGF1R, FGFR1,	124	71	6910	3.92435257	0.99853995	0.06642062	37.1256274
KEGG_PATHWAY	hsa05206:MicroRNAs in cancer	20	16.1290323	5.41E-07	PRKCA, EGFR, HRAS, PDGFB,	124	285	6910	3.91058291	9.30E-05	2.16E-06	6.61E-04
KEGG_PATHWAY	hsa04724:Glutamatergic synapse	8	6.4516129	0.00407217	PRKCA, GNG8, MAPK1, GNB2	124	114	6910	3.91058291	0.50432937	0.00943954	4.86559792
KEGG_PATHWAY	hsa04921:Oxytocin signaling pathway	11	8.87096774	4.80E-04	PRKCA, EGFR, MAPK1, NRAS,	124	158	6910	3.87964475	0.07924963	0.00130972	0.58507477
KEGG_PATHWAY	hsa04914:Progesterone-mediated oocyte maturation	6	4.83870968	0.0190852	MAPK1, IGF1R, KRAS, PIK3CA	124	87	6910	3.84315907	0.96364354	0.036963	20.9864562
KEGG_PATHWAY	hsa04022:cGMP-PKG signaling pathway	11	8.87096774	7.08E-04	MAPK1, CREB3L2, PIK3CA, CR	124	166	6910	3.69267392	0.11466959	0.00184367	0.86184863
KEGG_PATHWAY	hsa04620:Toll-like receptor signaling pathway	7	5.64516129	0.01129994	MAPK1, IFNAR2, IFNB1, IKBK(	124	106	6910	3.68000609	0.85838653	0.02384269	12.9698774
KEGG_PATHWAY	hsa04114:Oocyte meiosis	7	5.64516129	0.01285129	CCNE2, MAPK1, IGF1R, YWHA	124	109	6910	3.57872152	0.89190537	0.02583395	14.6245249
KEGG_PATHWAY	hsa05100:Bacterial invasion of epithelial cells	5	4.03225806	0.04980403	MET, PIK3CA, PIK3R5, FN1, PI	124	78	6910	3.57216708	0.99984729	0.08660542	46.4462188
KEGG_PATHWAY	hsa05166:HTLV-I infection	16	12.9032258	4.38E-05	HRAS, IL2RA, PDGFB, PDGFA,	124	256	6910	3.4828629	0.00750003	1.32E-04	0.05348879
KEGG_PATHWAY	hsa04380:Osteoclast differentiation	8	6.4516129	0.00862527	MAPK1, IFNAR2, IFNB1, CSF1	124	131	6910	3.40310268	0.77462311	0.01868378	10.0478139
KEGG_PATHWAY	hsa04922:Glucagon signaling pathway	6	4.83870968	0.03126987	CREB3L2, CREB3L1, CREB3L3,	124	99	6910	3.3773216	0.99576472	0.05706315	32.1821206
KEGG_PATHWAY	hsa04666:Fc gamma R-mediated phagocytosis	5	4.03225806	0.06220241	PRKCA, MAPK1, PIK3CA, PIK3	124	84	6910	3.31701229	0.99998405	0.10457818	54.389583
KEGG_PATHWAY	hsa04911:Insulin secretion	5	4.03225806	0.06441382	PRKCA, CREB3L2, CREB3L1, C	124	85	6910	3.27798861	0.99998937	0.10719509	55.6870473
KEGG_PATHWAY	hsa05169:Epstein-Barr virus infection	11	8.87096774	0.00197769	CDKN1A, YWHAZ, BCL2, GSK	124	190	6910	3.2262309	0.28858519	0.00485247	2.39086182
KEGG_PATHWAY	hsa05164:Influenza A	9	7.25806452	0.01215444	PRKCA, MAPK1, IFNAR2, IFNI	124	174	6910	2.8823693	0.87795584	0.02502343	13.8848819
KEGG_PATHWAY	hsa04110:Cell cycle	6	4.83870968	0.06976036	CCNE2, CDKN1A, YWHAZ, GS	124	124	6910	2.69640999	0.99999603	0.11479821	58.6851778
KEGG_PATHWAY	hsa05202:Transcriptional misregulation in cancer	8	6.4516129	0.02989423	IGF1R, CDKN1A, FLT1, PDGFA	124	168	6910	2.65360983	0.99459391	0.05575079	30.9954694
KEGG_PATHWAY	hsa04144:Endocytosis	12	9.67741935	0.006055	FGFR2, EGFR, IGF1R, HRAS, F(	124	258	6910	2.59189797	0.64817654	0.01347496	7.15519234
KEGG_PATHWAY	hsa04932:Non-alcoholic fatty liver disease (NAFLD)	7	5.64516129	0.0521006	RXRA, GSK3B, PIK3CA, PIK3R!	124	151	6910	2.58331553	0.99989928	0.08963537	48.0071531
KEGG_PATHWAY	hsa04024:cAMP signaling pathway	9	7.25806452	0.02455285	MAPK1, CREB3L2, PIK3CA, CR	124	198	6910	2.5329912	0.98609907	0.0469069	26.2049166
KEGG_PATHWAY	hsa04530:Tight junction	6	4.83870968	0.09693591	PRKCA, NRAS, HRAS, KRAS, F	124	137	6910	2.44054627	0.99999998	0.15517777	71.2453951

Supplemental data 4 for Figure 4A

BENPORATH_SOX2_TARGETS

> genes upregulated and identified by ChIP on chip as SOX2 targets in human embryonic stem cells.

AASDH	CAPRIN1	FAM158A	ILF3	NDUFA2	PWP1	SPIRE1	WDFY2
ABCB7	CAPZA2	FAM168A	ING4	NDUFB5	RAB15	SPRED1	WDR36
ABCF2	CARKD	FAM60A	INHBA	NDUFB8	RAB17	SRSF4	WDR77
ABHD11	CASP9	FAM63B	INO80C	NEBL	RAB25	SRSF7	WDR81
ABLIM1	CBX3	FAM76B	INO80D	NEDD4L	RAB3GAP2	SSBP3	WHSC1L1
ABTB2	CBX5	FAM96A	IRX2	NEMF	RAB4B	SSR4	XAB2
ACIN1	CBY1	FAM98A	IWS1	NFE2L3	RAB5A	ST3GAL2	XRRA1
ACO2	CCDC93	FANCC	JARID2	NIP7	RAD23A	STAP2	ZC4H2
ACOT8	CCDC94	FANCF	JUB	NIT1	RAD54B	STAT3	ZCCHC14
ACOX1	CCND1	FARSA	JUP	NKIRAS1	RANBP10	STC1	ZCCHC3
ACTR1A	CDC14B	FBXL14	KANK1	NKTR	RASA1	STXBP2	ZCRB1
ADAR	CDC42BPA	FBXL19	KAT6A	NME7	RASGRF2	SUFU	ZEB2
ADD3	CDC45	FBXO11	KCNMB4	NMU	RASL11B	SUGP2	ZFP36L1
ADRBK2	CDC7	FBXO16	KCNN2	NOC3L	RBBP9	SULF1	ZFYVE19
AK3	CDH2	FBXW11	KDM3A	NOL6	RBM22	TAF12	ZIC1
AKIRIN1	CDH3	FEM1A	KDR	NOTCH1	RBM23	TAF15	ZIC2
AKIRIN2	CDK1	FEZ1	KIAA0247	NSUN3	RBM39	TAL1	ZIC3
AKT1S1	CDK12	FGF2	KIAA0319L	NUCKS1	RBPMS	TALDO1	ZIK1
ALCAM	CDK14	FGFR1	KIAA0368	NUDT4	RCOR3	TARBP2	ZNF140
ALDH7A1	CDK16	FGFR2	KIAA0391	NUFIP2	RDH10	TBC1D10B	ZNF217
ALPL	CDK17	FICD	KIAA1143	NUP160	RDH11	TBC1D17	ZNF281
AMOTL1	CDK6	FIP1L1	KIAA1279	NUSAP1	REST	TBL1XR1	ZNF286A
ANKHD1	CDYL	FOXJ2	KIF11	OAZ2	RFX1	TBP	ZNF300
ANKRD1	CEP41	FOXN3	KIF15	OBSL1	RGS10	TCF20	ZNF331
ANO8	CEP89	FOXO1	KIF20B	ODF2L	RIC8B	TCF7L1	ZNF335
ANP32A	CEP95	FOXP1	KIF26A	OGT	RIF1	TCF7L2	ZNF428
ANP32B	CER1	FRAT2	KIF2C	OIP5	RIPK1	TDGF1	ZNF516
AP2A1	CETN3	FTL	KLF5	OLFML3	RMI1	TEAD2	ZNF646
AP3B1	CFL1	FUS	KLHL5	ORC1	RNF14	TGIF2	ZNF664
APEX1	CISD1	FXYD5	KNTC1	ORC6	RNF219	THBS2	ZNF668
APH1A	CLIC4	FZD1	KPNA3	OSBPL1A	RNF24	THOP1	ZNF677
APP	CLN3	FZD10	LAMA4	OSGEP	RNF31	TIA1	ZNF701
AQP2	CLP1	FZD2	LAMTOR2	OXA1L	ROR1	TIAL1	ZNF770
ARF3	CNOT8	FZD3	LARGE	PAK1	RPL15	TIMM23B	ZSCAN2
ARF4	COL12A1	FZD7	LARP7	PARG	RPL30	TIMM8B	
ARFGEF1	COMMD3	G3BP1	LASP1	PCBP1	RPL36A	TIMM9	
ARHGAP1	COMMD7	GADD45G	LEFTY1	PCF11	RPL7	TIMP4	
ARHGAP11A	COPB1	GBF1	LEFTY2	PCNXL3	RPL9	TIPIN	
ARID1B	COPS7A	GCFC1	LHPP	PCSK5	RPLP1	TJP3	
ARID4B	CPT1A	GDAP1	LINC00337	PDCL	RPS18	TLE1	
ARID5B	CREB3L4	GGA1	LINGO1	PDPN	RPS26	TLE2	
ARIH1	CRIM1	GGPS1	LOXL2	PER2	RPS29	TLE3	
ARL4D	CSNK1E	GJA1	LPHN1	PGAP3	RPS3A	TMEM108	
ARMC6	CTGF	GNA13	LRAT	PGM2L1	RRN3	TMEM109	
ARRDC3	CTH	GNAI1	LRFN3	PHAX	RRS1	TMEM123	
ASNA1	CXCL5	GNG10	LRP2	PHF17	RSF1	TMEM160	
ATAD2	CYB5R2	GNPTAB	LRRN1	PHF23	RSRC2	TMEM170A	
ATF3	CYR61	GPC6	LSG1	PHF8	RTN2	TMEM55B	
ATF4	DARS2	GRHL2	LSM3	PIF1	SALL1	TMEM63A	

ATP5F1	DCAF11	GRID2	LSM4	PIH1D1	SAP30	TMSB10
ATP6V1G1	DCAKD	GRK6	LSMD1	PIK3R3	SASH1	TNC
ATPBD4	DDA1	GSK3A	LYPD1	PIN4	SAT1	TNFAIP2
AUH	DDX17	GSK3B	MAGED2	PIP5K1C	SAV1	TNRC6A
AVEN	DDX21	GSPT2	MAN2C1	PIPOX	SC5DL	TOP2A
AZI1	DDX39A	GTPBP3	MAP3K11	PITX2	SCAF1	TP73-AS1
B3GALT4	DDX5	GXYLT1	MAP3K12	PLEKHA3	SCAF4	TPM3
B4GALT6	DHDDS	H2AFJ	MAPRE2	PLEKHG3	SCG3	TRA2A
BAMBI	DHRS3	H2AFX	MAST1	PLIN2	SCNM1	TRIM16
BCAT1	DHX38	H3F3B	MAT2B	PNISR	SCNN1A	TRIM22
BCKDHA	DIAPH3	HAS2	MCTS1	PNMA1	SDCCAG8	TRIM24
BCL9	DIDO1	HBP1	MDC1	PNP	SDHD	TRIML2
BCLAF1	DKK1	HDAC2	MED12	POLDIP3	SELRC1	TRIT1
BDH2	DNAJC16	HDAC9	MED17	POLE3	SEMA6A	TRPC4AP
BMP2	DNAJC2	HDGFRP2	MED25	POLR3E	SENP2	TSC22D1
BMP7	DNM2	HECTD2	METTL21D	POLR3G	6-Sep	TSTD2
BTF3L4	DPAGT1	HELLS	MEX3C	POU2F1	SERPINA1	TTF2
BTG1	DPP3	HEPH	MGEA5	POU5F1	SET	TUBB
BUB1B	DPPA4	HESX1	MKKS	PPAP2A	SF3B5	TUBG1
BUB3	DPYSL2	HEXIM2	MLH1	PPAPDC1B	SFPQ	TXNDC12
C11orf67	DPYSL3	HHEX	MLLT10	PPM1B	SFRP1	TXNRD1
C11orf73	DSCC1	HIF1AN	MOBP	PPM1N	SFRP2	UBC
C12orf35	DTNA	HIST1H4C	MORF4L1	PPP1R10	SFXN1	UBE2C
C12orf60	DUSP12	HIST2H2AA3	MORF4L2	PPP1R11	SGK3	UBE2D3
C14orf119	DUSP6	HIST2H2BE	MOSPD3	PPP1R15A	SGMS1	UBE2T
C14orf159	DVL2	HIST3H2A	MPND	PPP1R2	SGTA	UBE2W
C15orf29	E2F3	HLTF	MRPL15	PPP2R1A	SH3GL3	UBP1
C16orf80	EAPP	HMBOX1	MRPL43	PPP2R1B	SKA2	UBQLN4
C19orf43	EEF2	HMG20A	MRPL47	PPP2R3A	SKIL	UBR1
C19orf54	EGLN3	HMOX1	MRPS11	PPP2R3C	SLC38A2	UBR5
C1orf174	EGR3	HN1	MRPS18B	PPP2R5C	SLC39A1	UFD1L
C1orf21	EIF2C2	HNRNPA1	MRPS23	PRCC	SLC3A2	UFM1
C1orf54	EIF3F	HNRNPA2B1	MRPS27	PRDM14	SLC44A1	UGT8
C1orf55	EIF4G2	HNRNPC	MRPS31	PRDX1	SLC7A11	UIMC1
C20orf194	ELAVL2	HNRNPK	MSC	PRKAR1A	SLC7A5	UQCR10
C20orf96	ENPP2	HNRNPL	MSL3	PRKCDBP	SLC7A5P1	URM1
C22orf13	ENSA	HNRNPUL1	MTF2	PRKRIP1	SLC7A5P2	USO1
C3orf75	EOMES	HSD17B12	MTM1	PRNP	SMAD3	USP25
C5orf4	EPHA1	HSP90AB1	MTSS1	PRPF19	SMARCAD1	USP44
C5orf62	EPM2AIP1	HSP90B1	MUDENG	PRPF38A	SMG7	USP49
C6orf130	ERBB2	HSPA13	MUS81	PRPSAP1	SNAPC1	USP7
C8orf42	ERRFI1	HUWE1	MYNN	PRR11	SNAPC3	VASH2
C9orf5	ETHE1	ICMT	MYO9A	PRSS8	SNRPA	VASP
CA2	EXOC5	ID1	NAA30	PSEN2	SNRPD3	VAT1
CA4	EXOSC5	IDH3G	NAALAD2	PSMB1	SNRPN	VCAN
CABLES1	EXOSC9	IER5L	NANOG	PSMC2	SNX1	VIM
CACHD1	EXPH5	IFI16	NBAS	PSMG2	SORT1	VPS35
CACNA1A	FAM100B	IGF2BP3	NBR1	PTN	SOX2	VPS52
CACNA2D1	FAM115A	IGFBP2	NCBP1	PTPN2	SP2	VRTN
CALM2	FAM124A	IK	NCDN	PTPN3	SPAG9	VWA5A
CALR	FAM134C	ILF2	NDUFA11	PUM1	SPCS2	WBP11
